# Implementation Science in the Development of a Care Pathway for Chronic Chagas Disease: An Experience from a Municipality in Minas Gerais

**DOI:** 10.1590/0037-8682-0381-2025

**Published:** 2026-02-09

**Authors:** Ana Carolina de Oliveira Gonçalves, Nayara Ragi Baldoni, Nayara Dornela Quintino, Wanessa Campos Vinhal, Bruno Henrique Faria Melo, Sarah Rocha Dessimoni, Taise Aparecida Rodrigues Ferreira Santos, Katiuscia Francisca Ferreira Oliveira, Carlos Henrique Valente Moreira, Laura Marques Azevedo, Ariela Mota Ferreira, Ester Cerdeira Sabino, Antônio Luiz Pinho Ribeiro, Claudia Di Lorenzo Oliveira, Maurilio de Souza Cazarim, Clareci Silva Cardoso

**Affiliations:** 1 Universidade Federal de São João del-Rei, Programa de Pós-Graduação em Ciências da Saúde, Divinópolis, MG. Brasil.; 2 Universidade de Itaúna, Itaúna, MG, Brasil.; 3 Secretaria de Estado de Saúde de Minas Gerais, Regional Divinópolis, Divinópolis. MG, Brasil.; 4 Secretaria Municipal de Saúde de Nova Serrana, Nova Serrana, MG, Brasil.; 5 Sutter Santa Rosa Family Medicine Residency, Santa Rosa, CA, USA.; 6 Universidade de São Paulo, Faculdade de Medicina, Instituto de Medicina Tropical, São Paulo, SP, Brasil.; 7 Universidade Estadual de Montes Claros, Programa de Pós-Graduação em Ciências da Saúde, Montes Claros, MG, Brasil.; 8 Universidade Federal de Minas Gerais, Departamento de Medicina Interna da Faculdade de Medicina e Serviço de Cardiologia do Hospital das Clínicas, Belo Horizonte, MG, Brasil.; 9 Universidade Federal de Juiz de Fora, Departamento de Ciências Farmacêuticas da Faculdade de Farmácia, Juiz de Fora, MG, Brasil.

**Keywords:** Chagas disease, Primary health care, Chronic care model

## Abstract

**Background:**

Chagas disease (CD) is a neglected endemic infectious disease. Primary health care (PHC) is responsible for delivering care to patients with CD in an integrated manner with other levels of the health system. We aimed to describe the use of implementation science (IS) as a tool for developing a care pathway for patients with CD from the perspective of the Brazilian Chronic Care Model (BCCM).

**Methods:**

This study was conducted in the large municipality of Minas Gerais, Brazil. A diagnostic phase was conducted to identify barriers and facilitators related to CD care. The findings from this stage were analyzed using IS frameworks. Subsequently, the BCCM was adapted as a logical model to guide the creation of a care pathway. Health managers and professionals from the municipalities were trained to implement the proposed actions. After implementation, process and outcome indicators were monitored over a 12-month period.

**Results:**

IS proved to be an effective strategy for applying BCCM to patients with CD in PHC settings. A total of 267 health professionals were trained. After the intervention, the following indicators increased: risk factor screening, serological testing, diagnosis, and antiparasitic treatment for CD.

**Conclusion:**

The development of a CD care pathway using IS tools integrated with the BCCM enabled the incorporation of processes into PHC and suggested that this model may be replicated in other contexts as well as for other chronic conditions requiring longitudinal care.

## INTRODUCTION

Chagas disease (CD) affects approximately 7 million people worldwide. It is endemic to 21 countries in the Americas, including Brazil, where it affects approximately 3.7 million people^1^. Presents with an acute phase characterized by nonspecific symptoms and a chronic phase marked by an asymptomatic period, known as the indeterminate form, which may account for 42.6% of cases. The symptomatic chronic phase may take years to manifest and can present with cardiac involvement (42.7%), digestive involvement (17.7%), or both (10.2%)^2^ ^-^ ^5^.

CD is the leading cause of death among neglected tropical diseases^6^ ^,^ ^7^, with a mortality rate of 3.14/100,000 population^8^. Its chronic form became a notifiable condition in Brazil in 2020, beingeffectively implemented only in 2023, following the end of the COVID-19 pandemic (2022) and subsequent efforts by the Ministry of Health^9^. Fewer than 10% of patients are diagnosed and <1% receive appropriate treatment^10^. The Clinical Protocol and Therapeutic Guidelines (PCDT) for CD were launched in 2018 by the Brazilian Ministry of Health with the aim of standardizing practices in a context where uncertainties regarding disease management persist, particularly within primary healthcare (PHC)^11^.

Patients treated with antiparasitic therapy have a reduced likelihood of developing CD-related complications, and have lower risks of cardiovascular and overall mortality^2^ ^,^ ^10^ ^,^ ^11^. Antiparasitic treatment is recommended by the PCDT using benznidazole (BZN), which is available free of charge through the Brazilian Public Health System (*Sistema Único de Saúde -* SUS)^11^. However, many physicians remain unfamiliar with the treatment of CD and report insufficient professional training, which leads to uncertainty regarding diagnosis, clinical management, and antiparasitic therapy^12^ ^,^ ^13^. Therefore, strategies aimed at organizing CD care within PHC are essential to ensure care for affected patients^11^ ^,^ ^12^.

PHC is the coordinating axis of care for chronic CD^11^ and the Brazilian Ministry of Health adopts the Brazilian Model of Care for Chronic Diseasesthe Brazilian Chronic Care Model (BCCM)^12^. This framework integrates the Chronic Care Model (CCM)^14^ and Risk Pyramid Model^15^ and social determinants of Health Model^16^. It was specifically designed for the Brazilian SUS, considering the structure of care networks, levels of support, patient self-care capacity, community engagement, and influence of the environment and lifestyle. This model calls for the development of a logical model that details both macro- and micro-level work processes. Although scientific evidence supports its effectiveness, its application in real-world contexts still faces operational challenges in the organization of health care delivery^17^.

Implementation Science (IS) provides methodological approaches that support the systematic adoption of research findings and other evidence-based practices in real-world contexts aimed at improving the quality and effectiveness of health services. IS has become particularly relevant in low- and middle-income countries because it promotes the uptake of cost-effective^18^ ^-^ ^20^ and sustainable^21^ interventions. Therefore, we aimed to describe the adaptation of BCCM for the care of patients with chronic CD within PHC using an IS approach.

## METHODS

### Study design

Derived from an IS study developed by Moreira et al.^22^ in three municipalities of Minas Gerais, this study focused on the municipality of Nova Serrana and provides a technical report on how the BCCM was adapted to organize PCDT guidelines in PHC settings.

### Study Population and Setting

The target population and study settings have previously been described in detail by Moreira et al.^22^. Nova Serrana had 105,552 inhabitants^23^ and was located in the central-west region of Minas Gerais. It receives a significant number of migrants from areas that are highly vulnerable to CD transmission, such as northern Minas Gerais and southern Bahia^23^ ^,^ ^24^. Since 2018, it has become municipality with full management responsibilities for health services. The local PHC network comprises 25 units and includes 24 family physicians, 26 nurses, 140 community health agents (CHAs), one cardiologist, and an additional 92 professionals, including managers and other health workers. These professionals were the target groups for the proposed implementation interventions.

### Intervention

**FIGURE 1:**
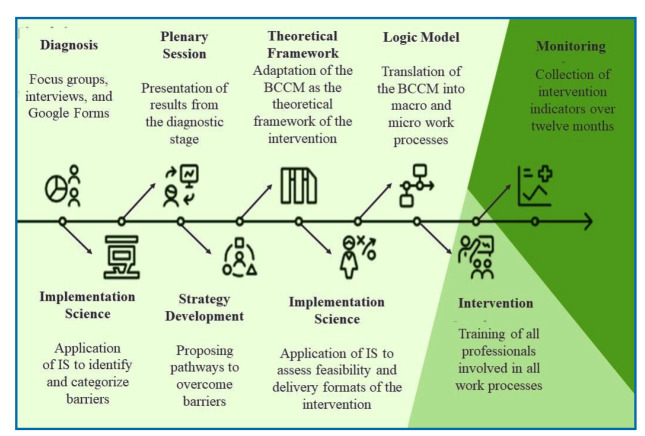
Depiction of the implementation process in the municipality and adaptation of the BCCM.

The study was carried out in three stages (Figure 1).

***Stage 1 - Preimplementation and design of the care pathway:*** In the diagnostic phase, key informant interviews were conducted with managers, nurses, pharmacists, nursing technicians, community health agents, and endemic disease control agents, along with focus groups involving all PHC physicians. Questionnaires were administered to all health professionals to assess the epidemiological and care contexts level of organization of CD care.

***The material was analyzed using IS frameworks:*** the Theoretical Domains Framework (TDF), to identify critical issues and strengths, and the Capability, Opportunity, and Motivation model (COM-B), to understand the factors influencing behavior change^22^.

The results were presented in a plenary session with health workers, and municipal and state managers. Shared strategies to overcome these barriers were developed using the theoretical framework of the BCCM^14^. Discussions were conducted in partnership with municipal and state health management and guided by the Brazilian Ministry of Health^11^ and State Health Secretariat guidelines.

The interventions were translated into a logical model of the CD care pathway, encompassing macro- and micro-level work processes, as outlined in the BCCM. Macroprocesses represented the overall structure of care, whereas microprocesses detailed the operational steps required at each stage.

***Stage 2 - Implementation:*** The feasibility of proposed interventions using the APEASE framework: Acceptability, Practicability, Effectiveness, Affordability, Side effects, and Equity, was assessed using the APEASE framework. In conjunction, the Behavior Change Wheel (BCW) and Behavior Change Techniques (BCTs) were applied to define strategies for promoting behavioral change and their modes of delivery^22^. materials were developed for health care teams^22^.

Training workshops targeting all involved health professionals were conducted by the research team in collaboration with state health management professionals. The implementation process lasted for 3 months, during which the research team provided support and made adaptations and adjustments to the model as needed^22^.

***Stage 3 - Monitoring*:** indicators were monitored over a 12-month period. The baseline (preimplementation) was established retrospectively 12-months prior to the commencement of the study, contemplating. The number of serological tests and etiological treatments.

The indicators were established for each macro-process and collected by the municipality using health information systems. Bimonthly meetings were held with the research team, municipal managers, and professionals from the Minas Gerais State Health Secretariat and Divinópolis Regional Office (SES-MG/Regional Divinópolis) to discuss potential adjustments. The indicators included: 1 - Screening risk factors (RF): this indicator was recorded through two distinct processes: a) Application of a standardized protocol to families during home visits by Community Health Agents (CHAs); and b) Routine electrocardiogram (ECG) performed in PHCs: questions regarding risk factors were incorporated into the Telehealth Network of Minas Gerais (RTMG) platform, which provides tele-electrocardiogram (Tele-ECG) services available to PHC facilities^11^. 2 - Serological testing and case notifications in the surveillance system: this indicator was monitored through the municipality’s control spreadsheet using data extracted from the Laboratory Environment Manager (GAL) of the Ezequiel Dias Foundation (FUNED), the reference laboratory for CD serology in Minas Gerais. Positive cases were confirmed by two reactive serological tests using different methods (Enzyme-linked Immunosorbent Assay, Chemiluminescence Assay, and Indirect Immunofluorescence, or Hemagglutination)^11^. Positive cases were reported as having chronic CD on the e-SUS Notifica platform, the official notification system of the SUS^25^. 3) Etiological treatment: Positive cases were referred for assessment of eligibility for BZN treatment in accordance with the Brazilian PCDT: a) patients aged <50 years with the indeterminate or digestive form; b) Patients with ECG abnormalities classified as mild cardiac form (left ventricular ejection fraction > 40%); c) patients aged >50 years were considered eligible subject to individual clinical evaluation. The following treatment indicators were monitored: number of treatments completed, treatment discontinuation owing to adverse events or nonadherence, and number of patients deemed ineligible for treatment^11^.

### Data analysis

Baseline and monitoring data were recorded using Microsoft Excel 2019. Descriptive analyses of frequency distributions and percentages were performed.

### Ethical considerations and funding

This study was approved by the Research Ethics Committee of the University of São Paulo (protocol no. 6.045.314). Funding was provided by the National Institutes of Health (NIH), within the scope of the SaMi-Trop cohort (Grant no. U01AI168383) and the Coordination for the Improvement of Higher Education Personnel (CAPES), Funding Code 001, Process no. 88881.927452/2023-01. The participating municipality signed a letter of agreement affirming its consent to the activities and commitment to provide the necessary support for implementation of the study.

## RESULTS

***Preimplementation and design of care pathway*:** Diagnostic data which identified six barriers: (1) Limited knowledge regarding treatment during the indeterminate phase. 2 - Limited experience and familiarity with BZN use 3 - Diagnostic resources were available, but patient flow was poorly understood, and delays in releasing serological results were reported. 4 - Lack of clinical suspicion of chronic CD compounded by competing demands of routine care. 5 - Low awareness of asymptomatic CD among the population 6 - A highly mobile population with a heterogeneous profile and limited health literacy regarding self-care and use of health services.

These barriers were presented to health professionals during a plenary session. The proposed strategies include training in entomological surveillance for all endemic disease control agents, strategies to increase the suspicion of chronic CD, development of a patient care pathway across the entire health network, and provision of diagnostic tests necessary to classify the clinical form of CD. Additional proposals included access to echocardiography creation of an identification card for patients using BZNt, designation of a physician as a technical reference in CD to provide decision-making support, and working in partnership with specialists from the RTMG and the SES-MG/Regional Divinópolis for clinical discussion of positive cases requiring individualized evaluation.

**FIGURE 2:**
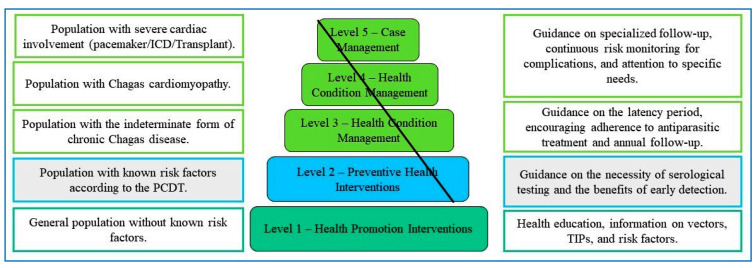
Chronic condition management model for Chagas disease.

Based on these proposals, the BCCM was adapted as a theoretical framework for intervention considering the population profile of the municipality, which includes a large number of migrants from hyperendemic regions, a lack of awareness of neglected diseases, and the complexity of cases progressing to severe forms of CD, establishing. of the levels of support, target population, and social determinants of CD (Figure 2).

**FIGURE 3:**
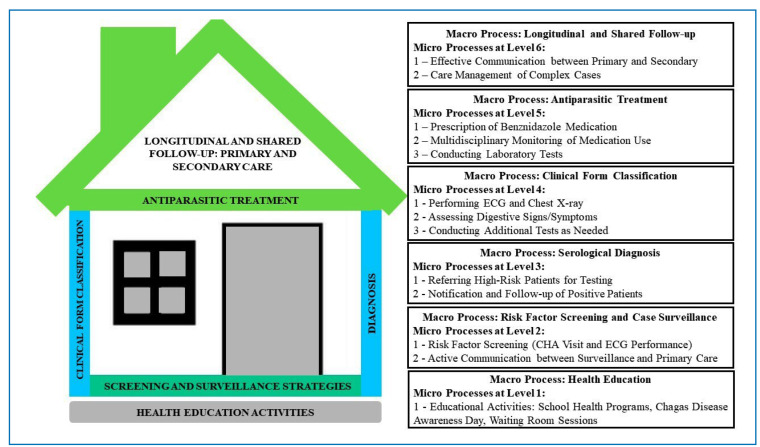
Logical model of the care pathway for chronic Chagas disease.

A logic model was developed comprising six macroprocesses: 1) Health education to raise community awareness around CD (etiological agent, transmission, risk factors, diagnosis, complications, and treatment). 2 -Screening: incorporation of RF investigation into routine PHC activities. 3 - Diagnosis - Timely identification of patients eligible for antiparasitic treatment. 4 - Clinical form classification of CD to direct patients toward appropriate treatment. 5 - Treatment: or management of CD-related complications. 6 - Longitudinal follow-up: ensuring continuous monitoring of patients, tracking disease progression, and enabling interventions aimed at preventing or delaying the onset of severe complications. Each macroprocess is subdivided into microprocesses, with all operational steps formalized in a guiding document (Figure 3). The foundation is health education activities, followed by an investigation of RFs aimed at addressing barriers related to the population’s limited awareness of CD and the lack of clinical suspicion regarding its chronic form. Subsequently, the stages of diagnosis and clinical classification were implemented. The microprocesses of treatment and longitudinal follow-up were designed to promote ownership of patient care by PHC teams in coordination with specialized care when required.

***Implementation:*** The intervention strategies including training, education, persuasion, and environmental restructuring. For environmental restructuring, all microprocesses of logic model are detailed in standard operating procedures (SOPs), operational workflows and educational materials for the population (Supplementary Material 1). These resources were designed to raise awareness of CD among the population and health professionals and, beyond organizational purposes, to serve as persuasive strategies for professionals regarding the need for appropriate care of these patients.

**FIGURE 4:**
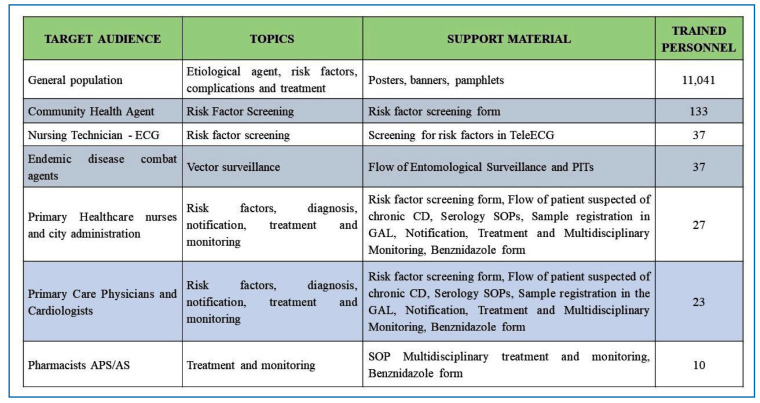
Target audience, topic, and support material of the training sessions conducted during the implementation phase.

To empower professionals and enhance their capacity for disease management, all individuals involved in the logic model received training to carry out the planned activities (Figure 4). Additionally, trained health professionals conducted health education activities for the general population and elementary school students, addressing topics such as vector identification, transmission routes, risk factors, diagnosis, treatment, and potential complications of CD (Supplementary Material 2).

Considering the possibility of workforce turnover and its potential impact on the sustainability of interventions, an admissions guide for healthcare professionals was developed. This guide introduced new staff to the local epidemiological situation of CD and emphasized the municipality’s care pathway for CD.

All supporting materials were developed by the study team under the supervision of the SES-MG, Divinópolis Regional Office, with additional technical approval from the Municipal Health Administration. The materials were made available on the official SES-MG website (http://vigilancia.saude.mg.gov.br/index.php/vigilancia-ambiental/) to allow other municipalities in the state to access and adapt them to their local contexts for structuring patient care for CD.

***Monitoring*:** All indicators were tracked by a health facility, which enabled the identification of silent units and targeting of specific interventions of each PHC unit. During bimonthly meetings to review the dashboard, the development of a CD educational leaflet was proposed, emphasizing the importance of early identification of indeterminate/chronic patients. This material was designed for use by CHAs to raise patient awareness regarding the need for serological testing when risk factors were identified during screening (Supplementary Material 3). A before-and-after comparison of the indicators is presented in Table 1. A marked increase was observed for all indicators. Serological testing increased by > 2,800%, patient diagnosis by > 1,700%, and antiparasitic treatment increased from 2 treated patients to 16, of whom 14 completed treatment and 2 discontinued before completion.

**TABLE 1:** Comparison of screening, serology, and treatment indicators in the periods before and after the intervention.

Indicators	Before		After		Diference	
	N	%	N	%	N	%
***Screening*** ^a^						
Number of families screened by CHA for CD RF	N/A	--	16,261	54	--	--
Number of individuals screened for CD RF by ECG	N/A	--	4,251	4	--	--
People with at least one CD RF	N/A	--	13,780	13	--	--
***Serology***						
Serology performed for CD^b^	51	0	1,525	11	1,474	2,890
Patients positive for CD^c^	3	6	55	4	52	1,733
***Treatment*** ^d^						
Patients treated with benznidazole	2	67	14	25	12	600
Patients who discontinued treatment due to adverse events	0	--	1	2	1	--
Patients who discontinued treatment due to nonadherence	0	--	1	2	1	--
Patients without criteria for specific treatment	1	33	9	16	8	800
Patients awaiting evaluation for specific treatment	0	--	30	55	30	--

**Legend: CHA:** community health agents **RF:** Risk Factor. **CD:** Chagas Disease. Twelve months before intervention. Twelve months after intervention. **N/A:** Indicator did not exist before intervention. References for percentage calculation: ^a^ municipal population; ^b^ Population at risk; ^c^ Population tested using serology; ^d^ Seropositive patients.

## DISCUSSION

This study used IS tools to develop care pathways for patients with CD. As a theoretical model, BCCM enables exploration of the complexities involved in the management of a neglected disease with a prolonged latency period. This required a reorganization of work processes, which had a positive impact on CD patient care; this methodology may also be replicated in the management of other chronic conditions or adapted for implementation in other municipalities.

Implementing scientific evidence within the context of the Brazilian SUS requires consideration of the complexity of a comprehensive and universal model of care^14^ ^,^ ^26^. The adaptation of BCCM through IS techniques allowed for a broader perspective of this model of care. The IS was able to synthesize and transform knowledge into practical actions^27^, which, through the BCCM, were structured into a logical model with defined work processes encompassing all aspects of care^14^. An additional strength is that this theoretical model has already been adopted in the context of SUS, specifically in healthcare networks, under the PlanificaSUS initiative^28^.

In PCDT, patients with chronic CD in the indeterminate and mild cardiac phases are included in the target population of PHC services^11^. In a study conducted by Santos et al.^29^ aiming to identify the social representations of neglected diseases, CD was not mentioned by health professionals as a condition of concern, contradicting the recommendations of both the World Health Organization (WHO) and the Brazilian Ministry of Health, which classify it as a priority among neglected diseases. Lack of professional knowledge, inadequate infrastructure, insufficient dialogue between PHC and specialized care, and limited awareness regarding pharmacological treatment have also been reported as challenges in the management of CD within PHC settings^30^. These findings reinforce the need to develop methodologies that systematize care and integrate CD into the PHC agenda.

A systematic review of the care of patients with CD^30^ presents strategies for more effective management, which corroborates the findings of this study. Screening and diagnosis, professional training, and community education were identified as essential, reinforcing the relevance of the macro-processes considered in the adaptation of BCCM. Accordingly, the results suggest that the BCCM facilitated the inclusion of CD as a condition of concern within PHC, as it integrates the three care models and translates complex situations into work processes aligned with the reality of PHC^14^ ^,^ ^15^.

From the perspective of organizing of SUS, the BCCM has been widely applied in different contexts, particularly for hypertension and diabetes^14^. However, it is important to highlight that the model is not limited to chronic diseases, but applies to any health condition requiring longitudinal care^28^, including persistent infectious diseases^31^. The CCM is internationally recognized and has been tested in different countries and primary care contexts. Evidence shows that its implementation has led to improvements in PHC, with components related to care organization, service delivery systems, and decision support consistently identified as fundamental^32^ ^,^ ^33^. In structuring the BCCM for CD, these components were incorporated, reinforcing the applicability of the model to other contexts.

Although the use of the BCCM is already a reality in Brazil, it entails profound changes involving the structure and organization of PHC. Reorganization of the work logic focused on a model of shared responsibility between service users and the health system, adopting a multiprofessional approach that addresses the condition from a systemic perspective^14^ ^,^ ^28^ ^,^ ^34^. In this regard, the use of IS as a guiding framework for the model stands out as a major in achieving results, given that process implementers are included in all stages of intervention design. Previous studies have corroborated the effectiveness of IS in strengthening healthcare systems in low- and middle-income countries, emphasizing its capacity to implement practices within PHC^19^ ^,^ ^21^.

Scientific evidence is often generated in highly controlled contexts; however, when applied in real-world, multiple contexts emerge, which can limit the applicability and clarity of indicators derived from “pure” evidence^35^. Thus, although PCDT^11^ compiles the best available evidence for the care of CD patients, its applicability is constrained by issues related to professional training, cultural factors, and health management^12^ ^,^ ^30^, as well as by the inherent invisibility of neglected diseases^29^. Therefore, IS proposes methodologies that integrate multiple methods and contexts, in addition to ensuring the participation of all stakeholders^21^ ^,^ ^36^, which can be essential for the long-term sustainability of interventions^37^.

Both IS and BCCM are strategies designed to regard multiplicity of contexts and foster the active participation of professionals involved in patient care, translating complex scenarios into systematic and routine procedures. Accordingly, the intervention presented herein may be applicable in other contexts, including municipalities with characteristics that differ from those of the study setting. Furthermore, implementation of the care pathway may encourage municipalities to organize work processes related to other health conditions, thereby strengthening PHC and improving the quality of services provided to patients.

Several challenges may contribute to the difficulty of CD diagnosis: the prolonged latency period, lack of clear standards for diagnostic investigation, and variability associated with environmental, geographic, social, and transmission-related factors hinder the establishment of a more accurate picture of the CD burden^38^. Thus, the substantial increase in the number of positive patients observed in this study is noteworthy. This finding indicates that the process of epidemiological vulnerability assessment proposed by the PCDT^11^ is an effective strategy for enhancing the timely identification of patients with the chronic form of the disease. The adoption of risk factor-screening strategies may yield significant advances in municipalities with similar epidemiological contexts.

The adoption of the proposed microprocesses addresses the main bottlenecks in the care of patients with chronic CD: clinical suspicion, diagnosis, and treatment^38^. The investigation of risk factors for CD during home visits by CHAs, along with ECGs, has enabled health teams to identify populations at risk within their territory, thereby allowing for targeted interventions to facilitate timely diagnosis.

However, this study presents aspects that warrant further discussion regarding future implementations. The intervention enabled an increase in case suspicion; however, it also highlighted that serological testing remains a challenge as coverage reached only 11% of the at-risk population. Nevertheless, when compared to the baseline, there was an increase of > 2,800%, demonstrating that although challenges persisted, the intervention led to substantial progress in expanding patient testing. Among the factors that might have influenced patient adherence to serological testing, an important issue was the need to travel to a laboratory for blood collection. The municipality has a labor context in which additional salary benefits are tied to the absence of missed workdays or hours. Adherence may also be affected by issues commonly associated with CD, such as the prolonged latency period and the social stigma surrounding the condition^39^ ^,^ ^40^.

A delay in the release of serological test results for chronic CD has also been identified as an important barrier. These tests were performed exclusively at the reference laboratory of the FUNED, which conducts serology for the entire state of Minas Gerais. Overcoming this barrier lies beyond municipal governance and is outside the scope of an intervention’s implementation capacity. Nonetheless, the establishment of additional reference laboratories with decentralized testing across regional hubs may represent a strategy to improve this work process and expedite the release of results.

Both critical points identified are related to serological testing. Previous studies have discussed the contribution of rapid diagnostic tests^41^ ^-^ ^43^ for CD; however, this methodology has not yet been implemented in the Brazilian Health System^11^. Therefore, the adoption of a rapid test as a screening tool could simplify the diagnostic process and reduce the workload of reference laboratories, potentially improving patient adherence and enhancing service organization, thereby addressing the limitations identified in this study.

## CONCLUSIONS

IS, combined with BCCM, proved capable of supporting the development of care models applicable to PHCs. The structuring of work processes for neglected chronic diseases based on this care model enabled the incorporation of practices by PHC, with CD integrated as a condition under PHC responsibility, and the shared management of more severe cases with specialized care. In the context of persistent challenges, initiatives aimed at organizing care for patients with neglected diseases have emerged as essential tools to strengthen public health services.

## Data Availability

Research data is only available upon request.
