# Supplementary material for: Implementation Science in the Development of a Care Pathway for Chronic Chagas Disease: An Experience from a Municipality in Minas Gerais
Source: Rev Soc Bras Med Trop. 2026 Feb 9;59:e0381-2025. doi: 10.1590/0037-8682-0381-2025 (PMC12892926; doi:10.1590/0037-8682-0381-2025)
Supplement: Supplementary material 1 [file 1678-9849-rsbmt-59-e0381-2025-md1.pdf]

PROCEDIMENTO OPERACIONAL PADRÃO – POP Nº5<br>REFERÊNCIA E CONTRARREFERÊNCIA PARA PACIENTES COM DOENÇA DE CHAGAS			
Data de Emissão<br>__/__/___	Data de Vigência<br>__/__/___ a __/__/___	Próxima Revisão<br>__/__/___	Versão nº 1

**Atividade:** Encaminhamento de paciente com Doença de Chagas para especialidades de cardiologia e/ou gastroenterologia.<br>**Executante:** Médicos da APS e Especialistas, Recepção da ESF, ACS, Setor e Regulação, Médico Regulador.
**Resultado esperado:** Padronizar os encaminhamentos entre a Atenção Primária e as Especialidades Médicas a fim melhorar a comunicação e o cuidado compartilhado e continuado aos pacientes.
**Materiais Necessários:** Caneta, ficha referência/contrarreferência

1. MÉDICOS DA APS

1.1 Preencher os campos da Ficha de Referência e Contrarreferência (Anexo A) com dados do cabeçalho completos.

1.2 Preencher a especialidade para a qual o paciente foi encaminhado. Se faz necessário preencher uma ficha para cada especialidade solicitada.

1.3 Preencher o campo ‘Dados Clínicos’ obrigatoriamente com os seguintes dados: resultado positivo para sorologia de Doença de Chagas, resultado do ECG, resultado do ECO e se realizou o tratamento antiparasitário.

1.4 Acrescentar outras informações que possam ser relevantes para o especialista.

1.5 Encaminhar o paciente à recepção da ESF para solicitação da consulta com o especialista.

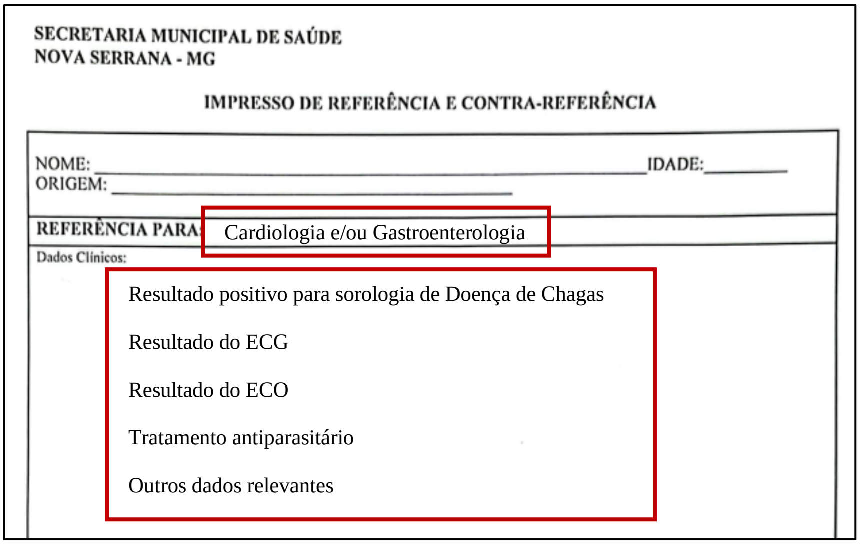
SECRETARIA MUNICIPAL DE SAÚDE
NOVA SERRANA - MG

IMPRESSO DE REFERÊNCIA E CONTRA-REFERÊNCIA

NOME: ____________________ IDADE: ________
ORIGEM: ____________________

REFERÊNCIA PARA: Cardiologia e/ou Gastroenterologia

Dados Clínicos:

Resultado positivo para sorologia de Doença de Chagas

Resultado do ECG

Resultado do ECO

Tratamento antiparasitário

Outros dados relevantes

2. RECEPÇÃO DA ESF

2.1 Solicitar cópias dos documentos: CI, CPF, cartão do SUS e comprovante de residência atual (mínimo 3 meses).

2.2 Conferir toda a documentação do paciente e anexar ao pedido.

2.3 Anexar cópias dos resultados de exames se tiver (laudo ECG e ECO).

2.4 Informar ao paciente os procedimentos de marcação.
2.5 Entregar ao paciente o protocolo da marcação.
2.6 Enviar o encaminhamento para regulação através da rota.

3. REGULAÇÃO

3.1 Conferir toda a documentação do paciente e devolver o pedido a ESF de origem se alguma inconformidade para regularização da mesma.
3.2 Cadastrar a solicitação no sistema CRESCER.
3.3 Direcionar o encaminhamento ao médico regulador.

4. MÉDICO REGULADOR

4.1 Avaliar o encaminhamento e em caso de inconformidades, devolvê-lo a ESF de origem para regularização.
4.2 Classificar o encaminhamento quanto a prioridade e direcioná-lo para a fila de agendamento.

5. REGULAÇÃO

5.1 Direcionar o encaminhamento a pasta de especialidade adequada (Cardiologia/Gastroenterologia)
5.2 Realizar o agendamento da consulta.
5.3. Anexar ao pedido a autorização de agendamento.
5.3. Encaminhar o pedido agendado via rota e comunicar a ESF de origem do paciente a data e local do agendamento.

6. RECEPÇÃO DA ESF

6.1 Receberá a rota e direcionará o agendamento ao ACS de referência do paciente.

7. ACS

7.1 Comunicar ao paciente o agendamento da consulta com o especialista, através de visitas ou contato telefônico, esgotando toda a possibilidade de aviso ao paciente.

8. MÉDICO ESPECIALISTA

8.1 Preencher a Ficha de Referência e Contrarreferência (Anexo A) obrigatoriamente com os seguintes dados: hipótese diagnóstica/diagnóstico, exames complementares solicitados, tratamento farmacológico prescrito com posologia, periodicidade de acompanhamento com especialista, se existe conduta específica para a interconsulta.
8.2 Avaliar se há alguma necessidade relacionada a equipe multiprofissional. Em caso positivo, descrever no encaminhamento para o direcionamento adequado da ESF.
8.3 Preencher nos pedidos de exames que o paciente se encontra em tratamento de Chagas.

EM CASO DE SOLICITAÇÃO DE RETORNO E/OU EXAMES COMPLEMENTARESO PROCESSO INICIA NOVAMENTE.

CONTRA-REFERÊNCIA:

Dados Clínicos, Hipótese Diagnóstica e Conduta

- Hipótese diagnóstica/diagnóstico
- Exames complementares solicitados
- Tratamento farmacológico prescrito com posologia
- Periodicidade de acompanhamento com especialista
- Conduta específica para a interconsulta
- Necessidade de acompanhamento multiprofissional

Anexo A – Modelo da Ficha de Referência e Contrarreferência

SECRETARIA MUNICIPAL DE SAÚDE
NOVA SERRANA - MG

IMPRESSO DE REFERÊNCIA E CONTRA-REFERÊNCIA

NOME: ______________________________ IDADE: ________
ORIGEM: ______________________________

REFERÊNCIA PARA:

Dados Clínicos:

CONTRA-REFERÊNCIA:

Dados Clínicos, Hipótese Diagnóstica e Conduta

PROCEDIMENTO OPERACIONAL PADRÃO – POP Nº 1<br>SOLICITAÇÃO DE SOROLOGIAS PARA SUSPEITA DE DOENÇA DE CHAGAS CRÔNICA			
Data de Emissão<br><br>__/__/___	Data de Vigência<br><br>__/__/___ a __/__/___	Próxima Revisão<br><br>__/__/___	Versão nº 1

**Atividade:** Solicitação de Sorologia para paciente com Suspeita de Doença de Chagas Crônica<br>**Executante:** Médico e/ou Enfermeiro, Recepção da ESF, Laboratório Municipal e Vigilância Epidemiológica.
**Resultado esperado:** Padronizar a solicitação de Sorologias para pacientes com suspeita de doença de Chagas Crônica no município de Nova Serrana
**Materiais Necessários:** Caneta, solicitação de exames padrão do município, ficha de Encaminhamento de Amostra da FUNED e carimbo do profissional.
**Referências:** Nota técnica nº 6/SES/SUBVS-SVE-DVAT-CZVFRB/2021

MÉDICOS E ENFERMEIROS

1. Receber comunicado de paciente apto ao rastreio sorológico para Doença de Chagas (DC) crônica a partir de investigação ou Agente Comunitário de Saúde (ACS) ou Técnico de TeleECG ou Médico suspeita durante a avaliação clínica ou Agente de Combate a Endemias (ACE).

2. Solicitar a sorologia para Doença de Chagas Crônica na solicitação padrão de exames laboratoriais (Anexo 1): sorologia IgG por dois testes sorológicos com métodos diferentes. Em caso de discordância realizar um terceiro teste. (ver Quadro 1).

Quadro 1. Teste diagnóstico para Doença de Chagas segundo a fase da doença.

FASE DA DOENÇA	TESTE DIAGNÓSTICO
**Crônica**	Combinação de dois testes baseados em princípios distintos ou com diferentes preparações antigênicas, que podem ser: ELISA, IFI, HAI, WB ou CLIA*.<br>- Teste rápido: caso seja negativo, o uso em testagem única, descarta a doença; teste positivo demanda confirmação diagnóstica com um dos testes acima.
**Em caso de sorologias discordantes**	Realizar um terceiro teste em diferente amostra de sangue, que pode ser: ELISA, IFI, HAI, WB ou CLIA

ELISA: ensaio de imunoabsorção enzimática; IFI: imunofluorescência indireta; HAI: hemaglutinação indireta; WB: Western blot; CLIA: quimiluminescência. *Pode ser utilizada a combinação de dois testes ELISA, desde que com preparações antigênicas diferentes.

3. Preencher a Ficha de Encaminhamento de Amostra da FUNED (Anexo 2): no campo ‘Dados clínicos do paciente’ assinalar outros e descrever ‘fator de risco conforme PCDT’.

4. Preencher no sistema ‘Crescer’ a solicitação de exames de acordo com os passos abaixo (figura 1):

4.1 Digitar na aba exames ‘Outros (SIA)’ o código do exame: 0202030776 - Pesquisa de anticorpos IGG *Antitrypanosoma cruzi*.

4.2 Selecionar uma opção (Sim / Não) nos campos “avaliado” e “solicitado”

4.3 Selecionar o CID B-57 doença de chagas (Médicos) ou CIAP-2 K-22 para (Enfermeiros).

4.4 Clicar na seta azul para acrescentar e em seguida finalizar o atendimento.

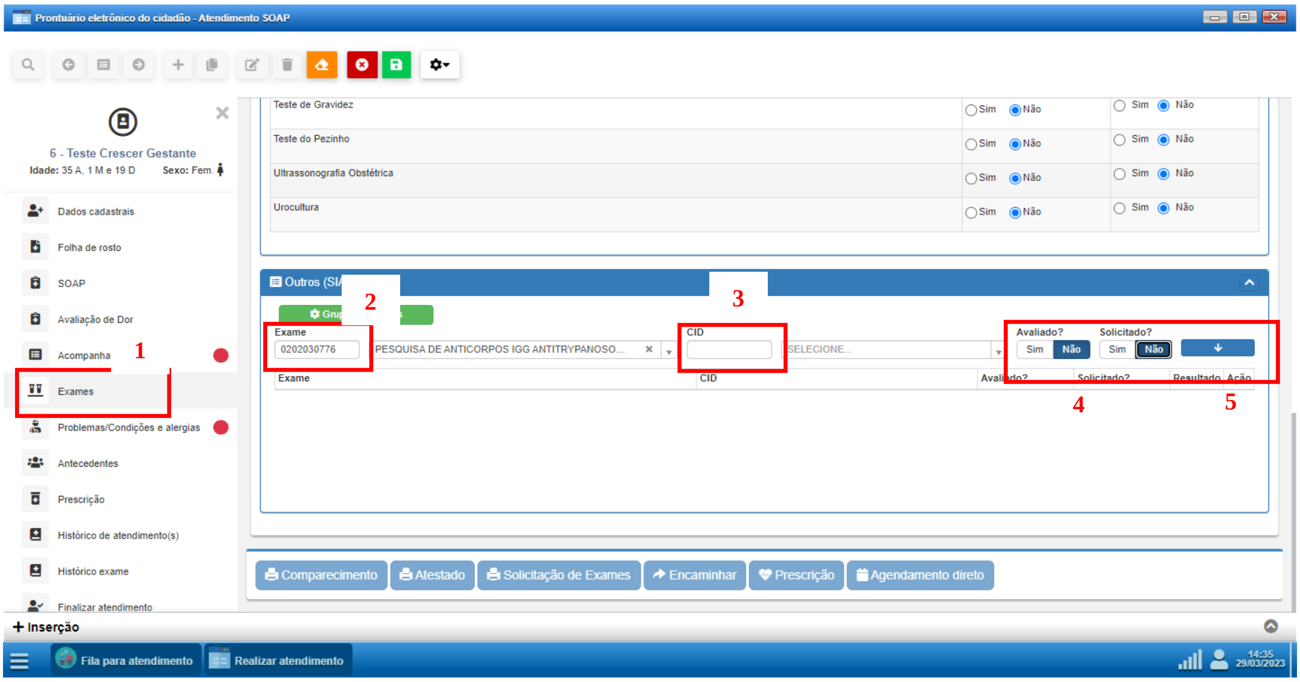

Figura 1 – Tela de solicitação de exames Sistema Crescer.

5. Orientar o paciente quanto ao tempo médio do resultado do exame e tranquilizá-lo informando que não há urgência quanto ao resultado

6. Encaminhar o paciente a recepção da unidade para agendamento do exame.

RECEPÇÃO DA ESF

1. Agendar a coleta do exame no sistema ‘Crescer’ para no mínimo dois dias após a data da marcação em função do fluxo da rota, de acordo com os passos abaixo:

1.1 Abrir o menu: Ambulatório / Solicitação / Agendamento direto de Serviço

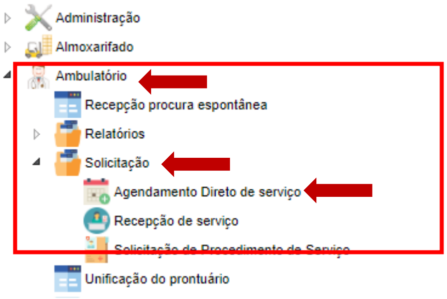

1.2 Escolher a opção agenda ‘Central’ e adicionar Unidade de Saúde ‘3 – Laboratório Central’ e serviço desejado ‘89 - Sorologia Chagas

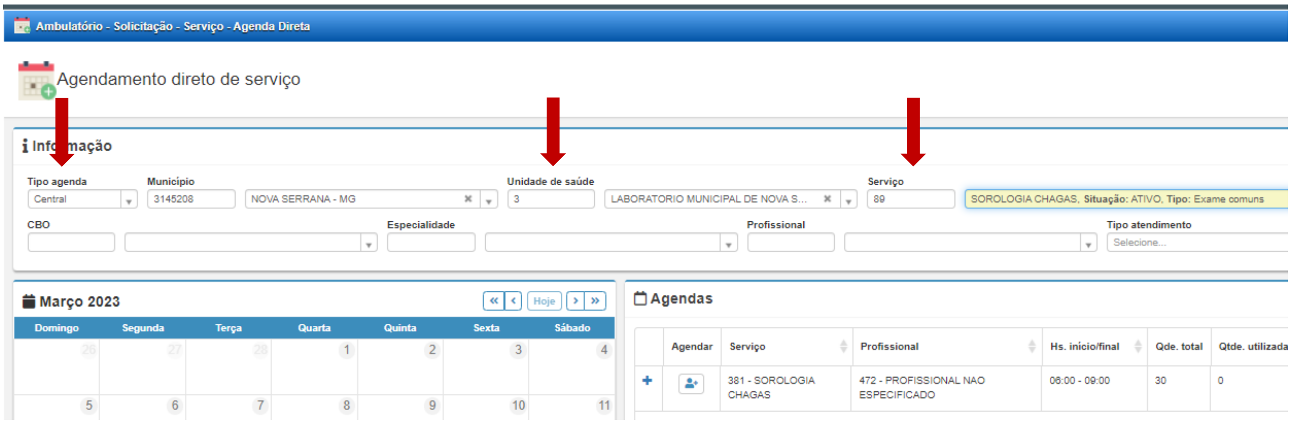

1.3 Selecionar a data do agendamento e clicar em agendar (lembrar do fluxo da rota – Espaçamento de 2 dias).

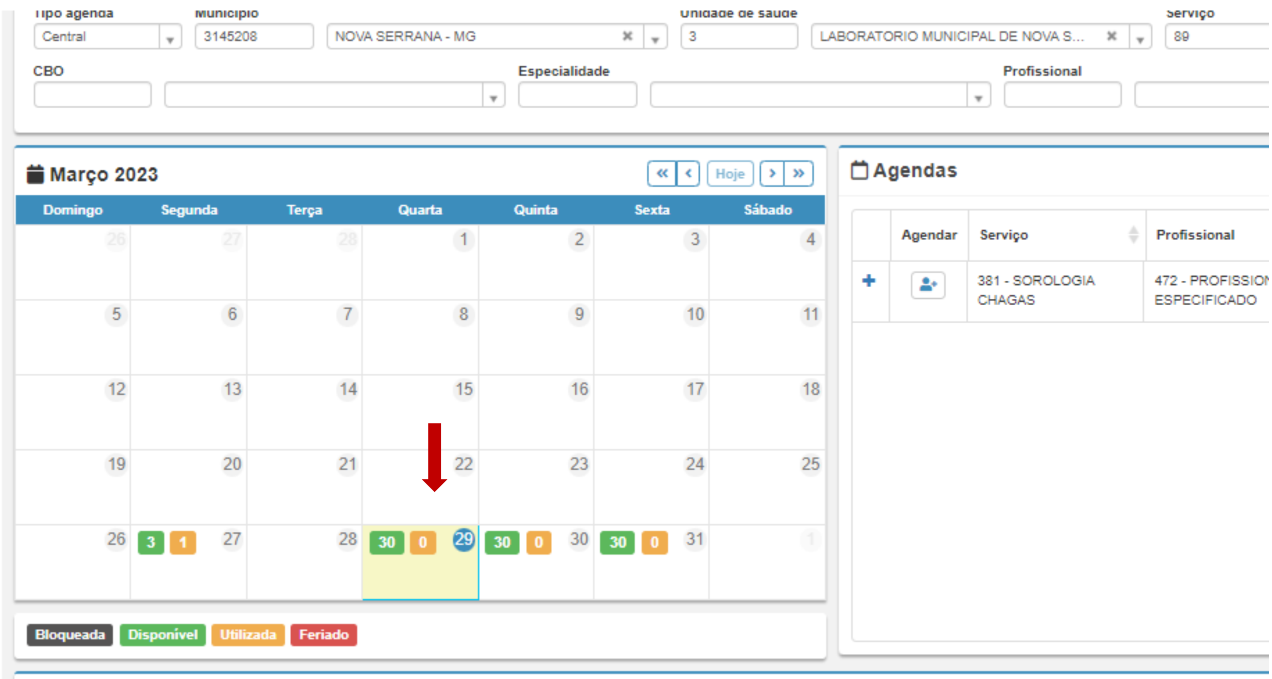

1.4 Selecionar o procedimento (Pesquisa de anticorpos IGG *Antitrypanosoma cruzi*.) e clicar em agendar

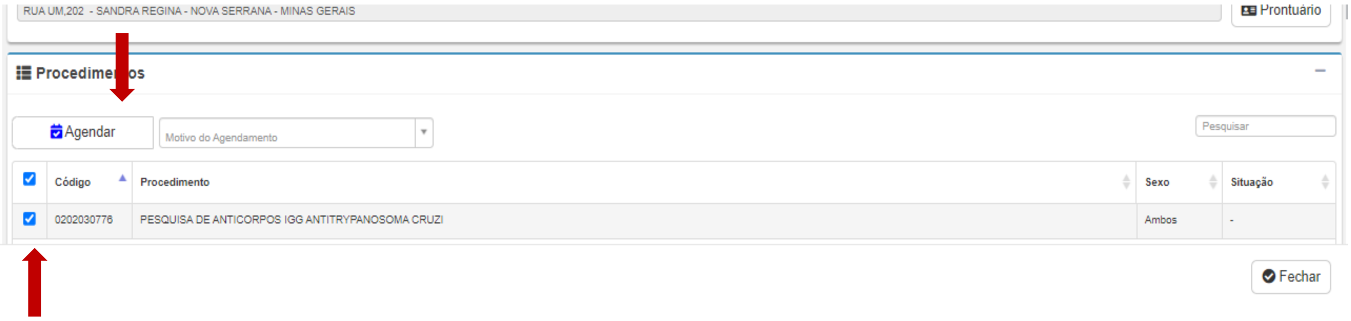

1.5 Clicar em ok após aparecer a tela de confirmação do agendamento

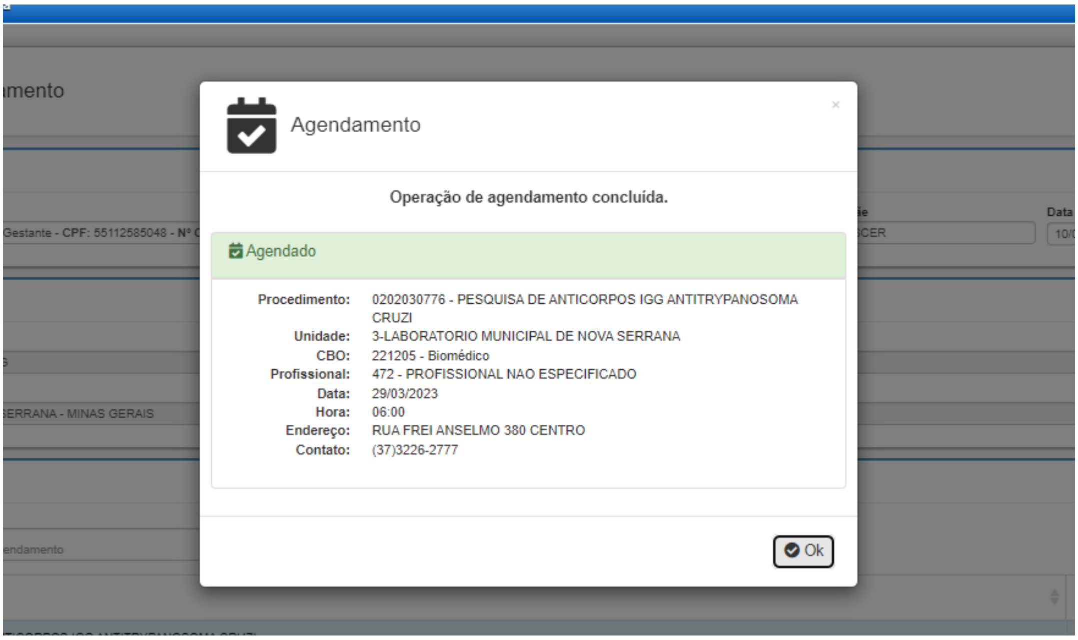

2. Reter o pedido do exame e a Ficha de Encaminhamento de Amostra da FUNED.

3. Entregar somente a confirmação de agendamento ao paciente e prestar orientações gerais.

4. Orientar o paciente quanto ao tempo médio do resultado do exame e tranquilizá-lo informando que não há urgência quanto ao resultado

5. Encaminhar o pedido do exame e a Ficha de Encaminhamento de Amostra da FUNED ao laboratório via rota.

LABORATÓRIO

1. Monitorar comparecimento do paciente para a realização do exame.

2. Informar a ESF de origem em caso de absenteísmo.

3. Realizar a coleta e cadastrar amostra no GAL (consultar POP Específico).

4. Enviar amostra a FUNED conforma fluxo do município.

VIGILANCIA EPIDEMIÓLOGICA

1. Monitorar os resultados de exames no GAL

2. Comunicar as ESFs os resultados das sorologias

3. Prestar orientações de notificação em caso de positividade

ANEXO 1 – MODELO DE SOLICITAÇÃO DE EXAME LABORATORIAL

PEDIDO DE EXAME LABORATORIAL

Paciente: ____

Idade________________ sexo [M] [F]

SOLICITO EXAMES:

- [ ] Hemograma completo
- [ ] Glicose em jejum
- [ ] Lipidograma
- [ ] HbA1c
- [ ] Hepatograma
- [ ] Insulina
- [ ] Uréia
- [ ] Creatinina
- [ ] Ácido úrico
- [ ] Triglicerídeos
- [ ] LDL-C
- [ ] HDL-C
- [ ] Colesterol Total
- [ ] Colesterol não HDL-C
- [ ] Tgo
- [ ] Cálcio
- [ ] Psa Livre
- [ ] Tgp
- [ ] Bilirrubina Total e Frações
- [ ] Fosfatase Alcalina
- [ ] GGT
- [ ] Tranferrina
- [ ] Ferro
- [ ] Ferritina
- [ ] Vitamina B12
- [ ] Vitamina B12
- [ ] Vitamina D
- [ ] Magnésio
- [ ] Potássio
- [ ] Fósforo
- [ ] TSH
- [ ] T3 e T4 Livre
- [ ] Sódio
- [ ] Sorologia pra Chagas (2 métodos)
- [ ] Outros____

Data____/____/_______

Assinatura o carimbo
do médico

ANEXO 2 – FICHA DE ENCAMINHAMENTO DE AMOSTRAS – DOENÇA DE CHAGAS CRONICA

Disponivel no link: http://www.funed.mg.gov.br/2018/10/vigilancia-saude/manuais-formularios-fichas-termos-de-coleta-de-amostras/

FUNED Fundação Ezequiel Dias

FUNDAÇÃO EZEQUIEL DIAS
INSTITUTO OCTÁVIO MAGALHÃES
Divisão de Epidemiologia e Controle de Doenças
Serviço de Doenças Parasitárias
Rua Conde Pereira Carneiro, 80 Belo Horizonte – MG
CEP: 30510-010 Tel: (31) 3314 4661 / 4663

I O M INSTITUTO OCTÁVIO MAGALHÃES

FICHA DE ENCAMINHAMENTO DE AMOSTRAS
EXAMES de DOENÇA DE CHAGAS CRÔNICA

PROCEDÊNCIA

* Instituição:			*Telefone/fax:
* Endereço da Instituição:			
* Nome do paciente:			*CNES:
* Nome da mãe:			
* Data de nascimento: ____/____/________	* Idade:	* CPF:	* Gênero: ☐ Masculino ☐ Feminino
* Responsável pelo envio:			

DADOS CLÍNICOS DO PACIENTE

Data dos 1º sintomas:___/___/____ Data de coleta: _____/_____/_______

Sinais e Sintomas:

☐ Assintomático ☐ Comprometimento Digestivo (megacólon, megaesôfago, etc)

☐ Chagoma de inoculação/ Sinal de Romanã ☐ Comprometimento Cardíaco (cardiomiopatia, sinais de ICC, etc)

☐ Febre Persistente ☐ Astenia

☐ Edema de face/ membros ☐ Adenopatia

☐ Outros_______________

EXAMES ANTERIORES

Sorologia: Data do exame: ___/____/____

☐ Imunofluorescência Indireta, IgM	☐ Reagente título:_____	☐ Não reagente
☐ Imunofluorescência Indireta, IgG	☐ Reagente título:_____	☐ Não reagente
☐ Ensaio Imunoenzimático Indireto	☐ Reagente	☐ Não reagente
☐ Reação de Hemaglutição Indireta	☐ Reagente	☐ Não reagente
☐ Reação de Quimioluminescência Indireta	☐ Reagente	☐ Não reagente

Parasitológico: Data do exame: ___/____/____

☐ Positivo ☐ Negativo

HISTÓRIA PRÉVIA DO PACIENTE

PROCEDIMENTO OPERACIONAL PADRÃO – POP Nº 2<br>CADASTRAMENTO DA AMOSTRA NO GERENCIADOR DE AMBIENTE LABORATORIAL (GAL)			
Data de Emissão<br>__/__/___	Data de Vigência<br>__/__/___ a __/__/___	Próxima Revisão<br>__/__/___	Versão nº 1
**Atividade:** Cadastramento da amostra no Gerenciador de Ambiente Laboratorial (GAL)<br>**Executante:** Laboratório municipal			
**Resultado esperado:** Padronizar o cadastramento de amostras no gerenciador de ambiente laboratorial de sorologia da doença de Chagas Crônica no município de Nova Serrana			
**Materiais Necessários:** Computador, internet e impressora.			

1. ACESSO AO GAL

1. Acessar o sistema GAL pelo link: https://gal.funed.mg.gov.br/, utilizando o navegador Mozilla Firefox;

2. Inserir o usuário e senha;

3. Selecionar o "MÓDULO BIOLOGIA MÉDICA" e o laboratório "GRS Divinópolis". Clicar em "Entrar". Abrirá uma tela de validação com números e letras. Digitar e clicar em ok.

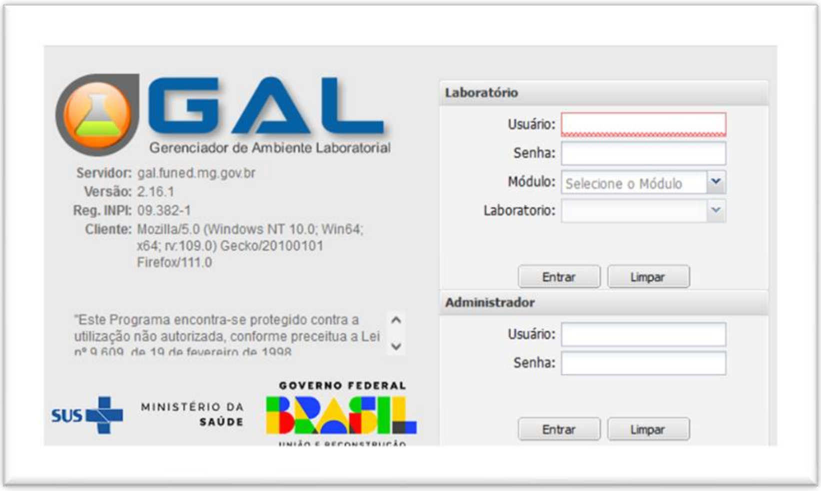

4. Abrirá a tela inicial. Selecionar a aba "BIOLOGIA MÉDICA", em seguida a aba "ENTRADA" e após "REQUISIÇÃO". Clicar em "REQUISIÇÃO" e posteriormente, clicar na aba superior da tela em "INCLUIR".

5. Abrirá a tela de digitar os dados da amostra que será enviada para a FUNED. Esta tela chama "INCLUIR REQUISIÇÃO". Ela é composta por 5 blocos de informações: 1) Requisição; 2) Paciente; 3) Informações Clínicas; 4) Notificação SINAN; 5) Amostras.

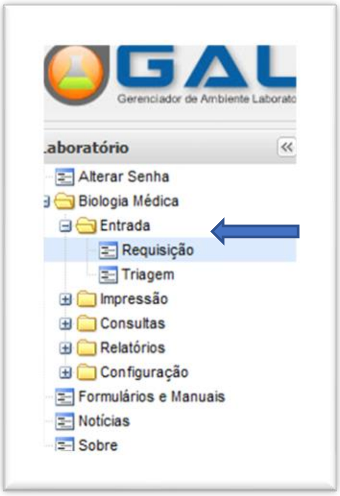

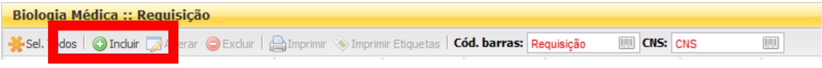

2. INCLUIR REQUISIÇÃO

1. Inserir dados do requisitante:

   1.1 Digitar o número do CNES da Unidade de Saúde solicitante ou o nome da Unidade de Saúde que os outros campos serão preenchidos;

   1.2 Digitar o nome do profissional de saúde e número de registro (CAMPOS OBRIGATÓRIOS). A solicitação pode ser realizada por qualquer profissional de saúde, não é obrigatório que seja pelo profissional médico.

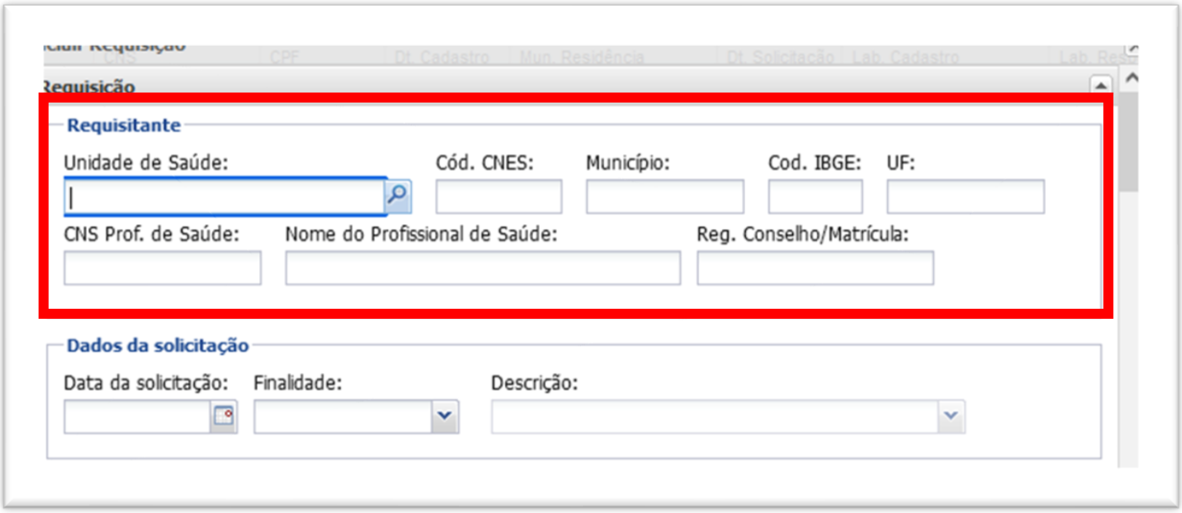

2. Inserir dados da solicitação:

   2.1 Digitar data da solicitação a qual deverá ser a mesma data do cadastro da amostra (CAMPO OBRIGATÓRIO);

2.2 Na finalidade colocar “INVESTIGAÇÃO” e na descrição “DOENÇA DE CHAGAS”.

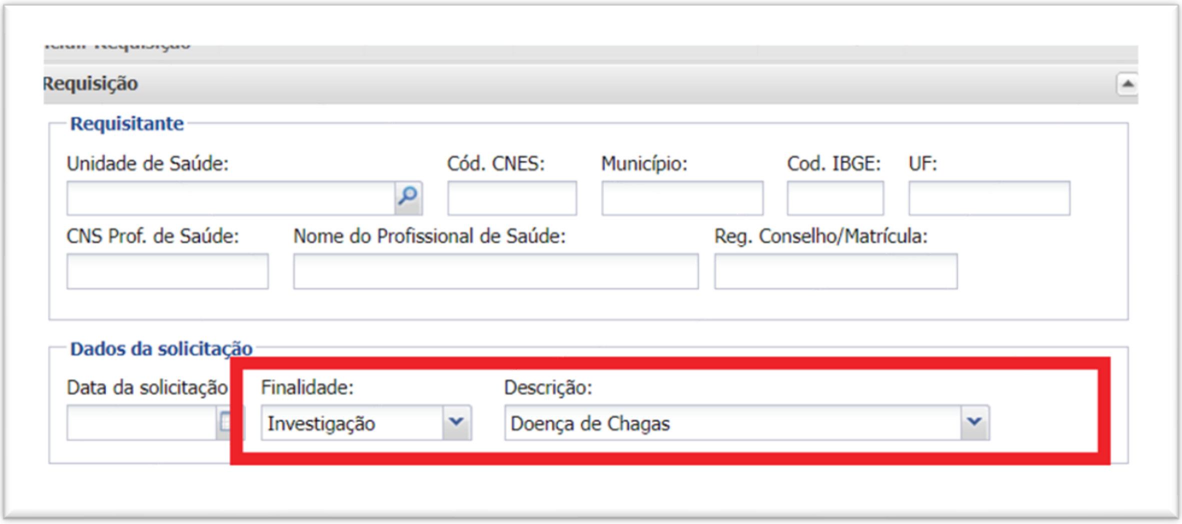

3. DADOS DO PACIENTE

1. Informar o CPF ou Cartão Nacional de Saúde (CNS) do paciente para o preenchimento automático dos campos de identificação pessoal.

OBS: O nome do paciente no cadastro no GAL tem que estar igual ao nome descrito no formulário padrão da FUNED para envio de amostra para exames de suspeita de Doença de Chagas Crônica “FICHA DE ENCAMINHAMENTO DE AMOSTRAS EXAMES de DOENÇA DE CHAGAS CRÔNICA”.

2. Após digitar o endereço do paciente

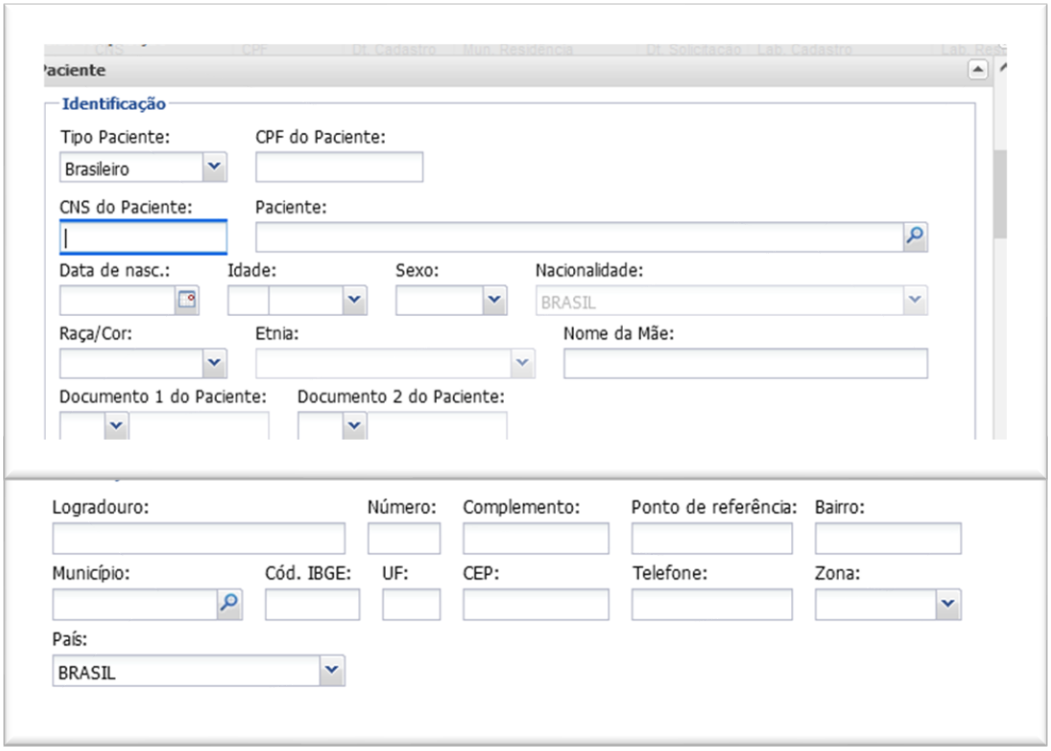

3. INFORMAÇÕES CLÍNICAS

1. - No campo “AGRAVO/DOENÇA”, inserir “Doença de Chagas crônica”.
2. - Os campos referentes a “Dados clínicos gerais” serão bloqueados para preenchimento. Seguir para as informações sobre “DETALHES DO CASO”. Informar no campo “ CASO” que é investigação de um caso “Suspeito”.

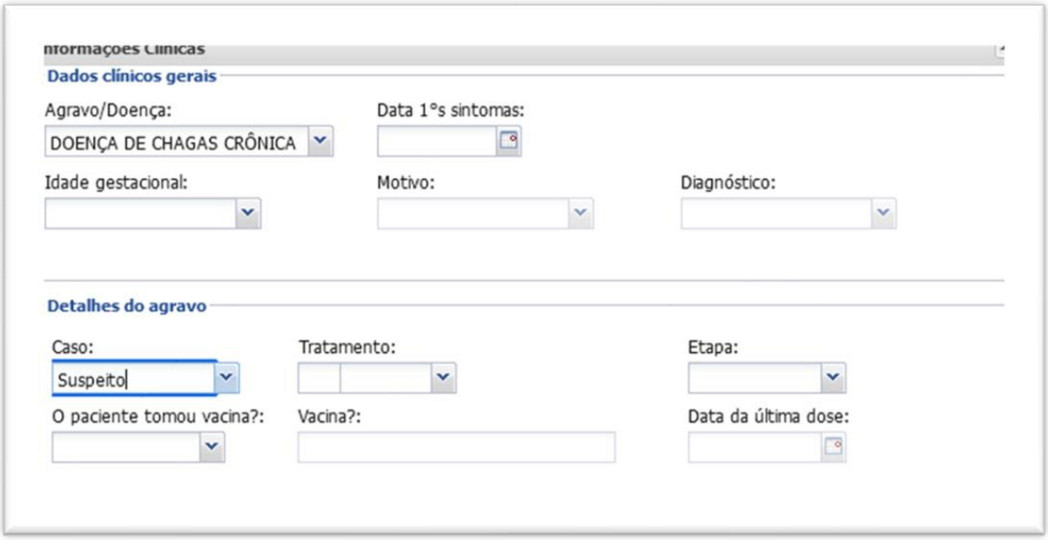

4. NOTIFICAÇÃO NO SINAN

1. - Deixá-lo em branco, considerando que só são notificados casos de Doença de Chagas crônica

5. AMOSTRAS

1. - No campo “AMOSTRAS”, deverão ser descritas todas as amostras encaminhadas do paciente.
2. - No campo “NOVA AMOSTRA” informar “Soro”, na caixa “AMOSTRA” informar “1”.
3. - Informar data da coleta (CAMPO OBRIGATÓRIO). Após clicar em “INCLUIR”

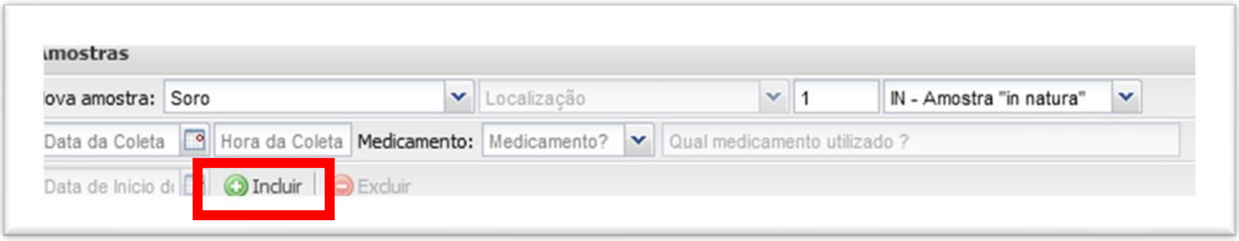

4. - Será adicionado os dados do material que será enviado para a FUNED.

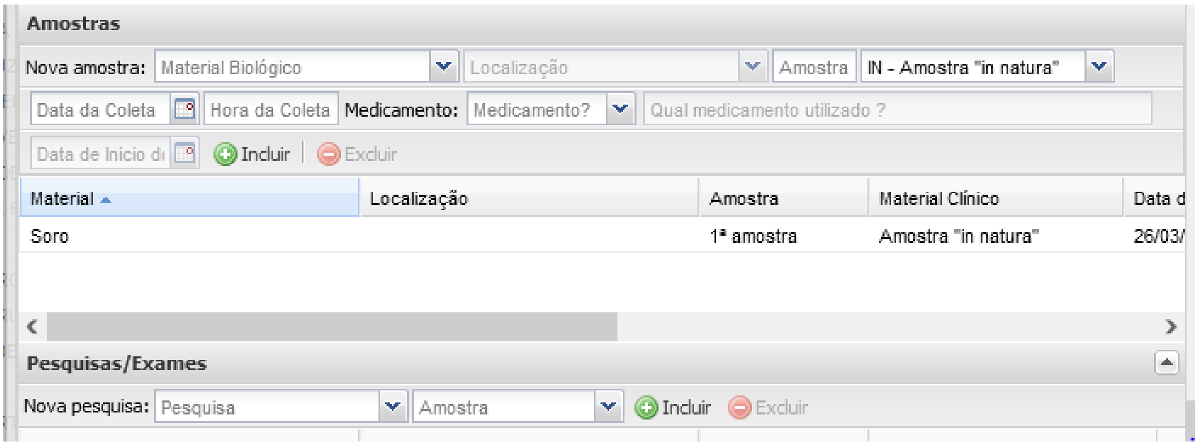

5. No campo "PESQUISA/EXAMES", selecionar o agravo referente à amostra enviada no campo "NOVA PESQUISA". Selecionar "Chagas crônica sorologia". E na aba "Amostra" clicar na seta e aparecerá o preenchimento só clicar em cima e clicar em "INCLUIR".

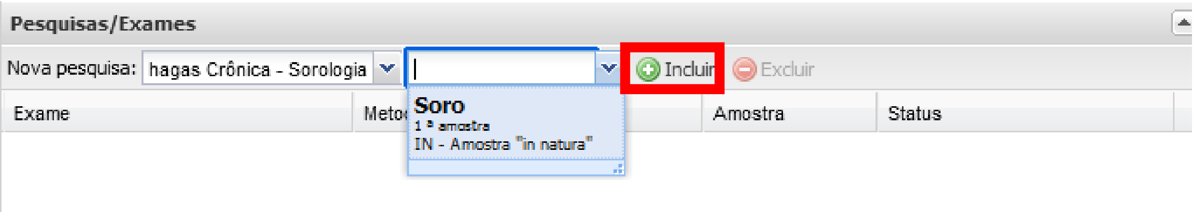

6. A amostra já foi incluída no GAL só clicar em "Salvar

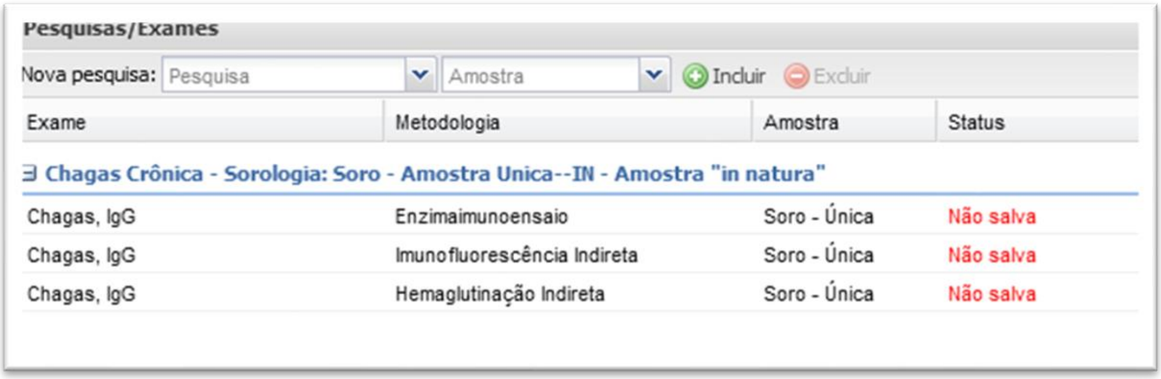

7. Após finalizar o cadastro a requisição deverá ser enviada para triagem. Retorne a aba inicial. Clicar em "TRIAGEM". Selecione a requisição que você quer enviar para a triagem na FUNED. Após clicar em "ENCAMINHAR PARA REDE".

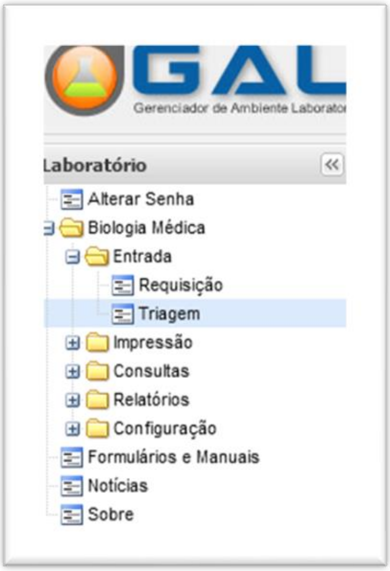

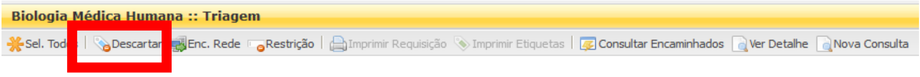

6. CONSULTAR SOLICITAÇÕES ENCAMINHADAS

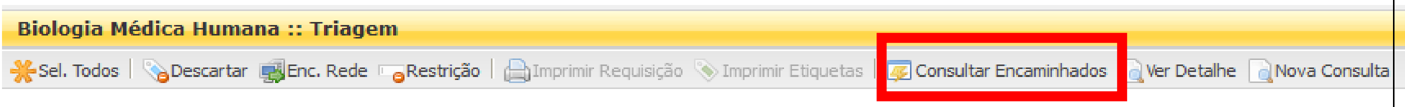

1. - Em “TRIAGEM”, clicar na aba “CONSULTAR ENCAMINHADOS” e siga os passos abaixo:
2. Digite o período em que a triagem foi realizada
3. Digite o laboratório de destino (Fundação Ezequiel Dias)
4. Clicar em imprimir

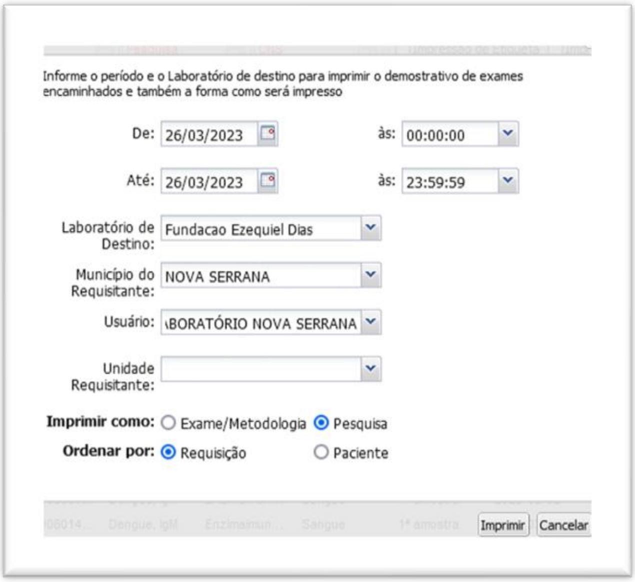

5. - Posteriormente, o GAL vai gerar um PROTOCOLO que apresenta todos os pacientes cadastrados, e que foram triados, assim como o exame que vai ser realizado, a metodologia e o tipo de amostra encaminhada. Imprimir e enviar junto com o soro o impresso da consulta de encaminhados + ficha padrão FUNED de envio de amostra para exames de suspeita de Doença de Chagas Crônica "FICHA DE ENCAMINHAMENTO DE AMOSTRAS EXAMES de DOENÇA DE CHAGAS CRÔNICA" (disponível em http://www.funed.mg.gov.br/2018/10/vigilancia-saude/manuais-formularios-fichas-termos-de-coleta-de-amostras/).

Município Requisitante: NOVA SERRANA
Origem: GRS - Divinopolis
Destino: Fundacao Ezequiel Dias
Período: 10/03/2023 às 00:00:00 **até** 12/03/2023 às 23:59:59

Requisição	Paciente	Exame	Metodologia	Material	Amostra		Usuário	Data	Unidade Requisitante	Município Requisitante	Lab. Cadastro
[illegible]	[illegible]	[illegible]	[illegible]	Swab	1ª amostra	000311414330	LABORATÓRIO NOVA SERRANA	10/03/2023 06:25:20	HOSPITAL SAO JOSE DE NOVA SERRANA	NOVA SERRANA	GRS - Divinopolis
[illegible]	[illegible]	[illegible]	[illegible]	Swab	1ª amostra	000311414330	LABORATÓRIO NOVA SERRANA	10/03/2023 06:25:20	HOSPITAL SAO JOSE DE NOVA SERRANA	NOVA SERRANA	GRS - Divinopolis
[illegible]	[illegible]	[illegible]	[illegible]	Swab	1ª amostra	000311414332	LABORATÓRIO NOVA SERRANA	10/03/2023 06:25:20	HOSPITAL SAO JOSE DE NOVA SERRANA	NOVA SERRANA	GRS - Divinopolis
[illegible]	[illegible]	[illegible]	[illegible]	Swab	1ª amostra	000311414332	LABORATÓRIO NOVA SERRANA	10/03/2023 06:25:20	HOSPITAL SAO JOSE DE NOVA SERRANA	NOVA SERRANA	GRS - Divinopolis
[illegible]	[illegible]	[illegible]	[illegible]	Swab	1ª amostra	000311414333	LABORATÓRIO NOVA SERRANA	10/03/2023 06:25:20	HOSPITAL SAO JOSE DE NOVA SERRANA	NOVA SERRANA	GRS - Divinopolis
[illegible]	[illegible]	[illegible]	[illegible]	Swab	1ª amostra	000311414333	LABORATÓRIO NOVA SERRANA	10/03/2023 06:25:20	HOSPITAL SAO JOSE DE NOVA SERRANA	NOVA SERRANA	GRS - Divinopolis

Recebido por:______________________________ **em** ____/____/____ **as** ____**h**____**m.**

PROCEDIMENTO OPERACIONAL PADRÃO – POP Nº 4<br>ACOMPANHAMENTO MULTIPROFISSIONAL DO TRATAMENTO ANTIPARASIÁRIO COM BENZONIDAZOL EM PACIENTES COM DOENÇA DE CHAGAS			
Data de Emissão<br>__/__/___	Data de Vigência<br>__/__/___ a __/__/___	Próxima Revisão<br>__/__/___	Versão nº 1

**Atividade:** Acompanhamento do Tratamento Antiparasitário com Benzonidazol em Pacientes com Doença de Chagas<br>**Executante:** Médico, Enfermeiro, ACS e Recepção da ESF.
**Resultado esperado:** Padronizar o acompanhamento de pacientes com doença de chagas em tratamento com benzonidazol no município de Nova Serrana.
**Materiais Necessários:** Ficha de acompanhamento Multiprofissional, computador com acesso à internet e ao prontuário eletrônico, carimbo do profissional e caneta.
**Referências:** Brasil. Ministério da Saúde, 2018. BRASIL. Protocolo Clínico e Diretrizes Terapêuticas Doença de Chagas. Brasília: Ministério da Saúde.

RECEPÇÃO DA ESF

1. Receber da farmácia a informação de que o paciente já está com o medicamento em mãos.
2. Informar ao ACS de referência a necessidade de realizar a visita domiciliar.
3. Informar ao médico prescritor que o paciente já se encontra com medicamento.

AGENTE COMUNITÁRIO DE SAÚDE

1. Realizar visita domiciliar de 5 a 7 dias após a dispensação do medicamento.
2. Verificar se o paciente iniciou o tratamento e se tem alguma dúvida.
3. Agendar uma consulta médica para aproximadamente 30 dias após o início do tratamento.
4. Realizar visita domiciliar de 15 e 18 dias após início do tratamento, informar sobre a data da consulta médica e lembrar o paciente sobre a necessidade da realização dos exames de monitoramento.
5. Verificar se o paciente permanece em uso adequado do medicamento ou tem alguma queixa.
6. Agendar uma consulta de enfermagem para o 45º dia de tratamento.
7. Realizar uma visita domiciliar 35 a 38 dias após início do tratamento, informar sobre a data da consulta de enfermagem e lembrar o paciente sobre a necessidade da realização dos exames de monitoramento.
8. Verificar se o paciente mantém o uso regular do medicamento ou apresenta alguma queixa.
9. Registrar na ficha de acompanhamento multiprofissional todas as visitas.

MÉDICO:

1. Providenciar dois pedidos dos exames laboratoriais para o monitoramento (após a informação da recepção):
   1.1 TGO, TGP, Creatinina, Hemograma com plaquetas e EAS, com a observação ‘Realizar em: __/__/__’.
   1.2 Data para a realização do 1º exame: 3 semanas após o início do tratamento (apresentar resultados na 1ª consulta, após 30 dias do início do tratamento).
   1.3 Data para a realização do 2º exame: 6 semanas após o início do tratamento (apresentar resultados na 2ª consulta, após 60 dias de tratamento).
2. Realizar uma consulta médica com aproximadamente 30 dias de tratamento.
3. Avaliar os exames de monitoramento, realizar exame físico completo e registrar no Cartão do BZN informações relevantes e o resultado dos exames.
4. Registrar na ficha de acompanhamento multiprofissional bem como no prontuário eletrônico.
5. Realizar uma consulta médica com aproximadamente 60 dias de tratamento.
6. Avaliar os exames de monitoramento, realizar exame físico completo e registrar no Cartão do BZN informações relevantes e o resultado dos exames.
7. Registrar na ficha de acompanhamento multiprofissional bem como no prontuário eletrônico.
8. Fazer o relatório médico de finalização do tratamento com BZN.

ENFERMEIRO:

1. Realizar uma consulta de enfermagem com aproximadamente 45 dias de tratamento.
2. Avaliar a adesão ao tratamento e realizar exame físico completo, com atenção para pele e parestesias.
3. Reforçar com o paciente a necessidade de realizar exames de monitoramento.
4. Registrar na ficha de acompanhamento multiprofissional, no cartão do BZN bem como no prontuário eletrônico.

OBS: EM CASO DE TRATAMENTOS SUPERIORES A 60 DIAS, MANTER CONSULTA MÉDICA COM 70 DIAS E AO FINAL DO TRATAMENTO (80 DIAS)

ANEXO A – FICHA DE ACOMPANHAMENTO MULTIPROFISSIONAL DO PACIENTE EM USO DE BENZONIDAZOL

FICHA DE ACOMPANHAMENTO DO PACIENTE EM TRATAMENTO COM BENZONIDAZOL

**PACIENTE:**		**DN.:** ____/_____/____
**INICIO DO TRATAMENTO:**	**DIAS DE TRATAMENTO:**	**OBSERVAÇÕES**<br>**(Detalhar qualquer informação/intercorrência neste campo)**
**UNIDADE DE REFERÊNCIA:**		
**1ª VISITA ACS** Data ___/____/_____<br>(5º ao 7º dia do início do tratamento)	Aderiu ao tratamento ( ) Sim ( ) Não - Detalhar<br>Alguma Queixa? ( ) Sim – Detalhar ( ) Não	
**2ª VISITA ACS** Data ___/____/_____<br>(15 a 18 dias do início do tratamento)	Aderiu ao tratamento ( ) Sim ( ) Não - Detalhar<br>Alguma Queixa? ( ) Sim – Detalhar ( ) Não	
**CONSULTA MÉDICA**<br>Data ___/____/_____<br>(30 dias do início do tratamento)	Permanece em uso do medicamento? ( ) Sim ( ) Não - Detalhar<br>Apresentou exames de monitoramento? ( ) Sim - Detalhar ( ) Não<br>Apresentou reações adversas? ( ) Sim - Detalhar ( ) Não.	
**3ª VISITA ACS** Data ___/____/_____<br>(35 a 38 dias do início do tratamento)	Aderiu ao tratamento ( ) Sim ( ) Não - Detalhar<br>Alguma Queixa? ( ) Sim – Detalhar ( ) Não	
**CONSULTA DE ENFERMAGEM**<br>Data ___/____/_____<br>(45 dias do início do tratamento)	Permanece em uso do medicamento? ( ) Sim ( ) Não - Detalhar<br>Alguma Queixa? ( ) Sim – Detalhar ( ) Não<br>Realizar Exame físico e detalhar	
**CONSULTA MÉDICA**<br>Data ___/____/_____<br>(60º dias do início tratamento)	Permanece em uso do medicamento? ( ) Sim ( ) Não – Detalhar<br>Apresentou exames de monitoramento? ( ) Sim - Detalhar ( ) Não<br>Apresentou reações adversas? ( ) Sim - Detalhar ( ) Não.	
**AVALIAÇÃO FINAL - MÉDICO**<br>Data ___/____/_____	O paciente finalizou o tratamento?<br>( ) Sim ( ) Não - Abandono ( ) Não - Reação Adversa<br>Quantos dias de tratamento o paciente realizou? _________	

PROCEDIMENTO OPERACIONAL PADRÃO – POP Nº 3<br>PRESCRIÇÃO E DISPENSAÇÃO DO BENZONIDAZOL			
Data de Emissão<br>__/__/___	Data de Vigência<br>__/__/___ a __/__/___	Próxima Revisão<br>__/__/___	Versão nº 1

**Atividade:** Prescrição, Solicitação e Dispensação do Benzonidazol e Acompanhamento do Tratamento de Pacientes com Doença de Chagas.<br>**Executante:** Médico e Farmacêutico.
**Resultado esperado:** Padronizar a solicitação do Benzonidazol para tratamento de pacientes com doença de Chagas no município de Nova Serrana
**Materiais Necessários:** Caneta, formulário de solicitação do Benzonidazol, prescrição médica, resultado da sorologia para doença de Chagas, carimbo do médico, ficha de notificação do E-SUS Notifica, computador com acesso à internet, impressora, papel.
**Referências:** Brasil. Ministério da Saúde, 2018. BRASIL. Protocolo Clínico e Diretrizes Terapêuticas Doença de Chagas. Brasília: Ministério da Saúde.

MÉDICOS

1. Preencher todos os campos do Formulário de Solicitação do Benzonidazol para o Tratamento de Pacientes com Doença de Chagas (Anexo A)
   1.1. Identificação profissional e identificação do paciente.

Figura 1 – Identificação do profissional e do paciente

GOVERNO DO ESTADO DE MINAS GERAIS
SECRETARIA DE ESTADO DE SAÚDE

ANEXO - FORMULÁRIO DE SOLICITAÇÃO DE MEDICAMENTOS PARA TRATAMENTO DA DOENÇA DE CHAGAS
Para uso pelo Município/Estabelecimento de saúde.

DADOS DO MÉDICO			
Nome:		CPF:	
CRM:		CNS:	
Telefone fixo:		Telefone celular:	

DADOS DO ESTABELECIMENTO DE SAÚDE SOLICITANTE			
Nome do estabelecimento:		CNES:	
Endereço:		CEP:	
Município:		Telefone:	

DADOS DO PACIENTE								
Nome:								
RG:		CPF:		CNS:				
Data de nascimento:	______/____/______	Idade:		Sexo:	Feminino	( )	Masculino	( )
Nome da mãe:								
Endereço domiciliar:								
Município:				CEP:				
Telefone fixo:				Telefone celular:				

1.2. Descrever brevemente a história clínica do paciente com dados sobre a forma clínica, local provável de infecção, exames realizados para confirmar o diagnóstico da doença de chagas e o laudo do eletrocardiograma.

Figura 2 – Antecedentes Clínicos

ANTECEDENTES CLÍNICOS (Descreva brevemente a história clínica do paciente com dados sobre forma clínica, local provável de infecção, exames realizados para confirmar o diagnóstico da doença de Chagas e laudo do eletrocardiograma)

1.3. Registrar qual a indicação do tratamento e medicamento prescrito, conforme Protocolo Clínico e Diretrizes Terapêuticas.

Figura 3 – Indicação do tratamento antiparasitário

INDICAÇÃO DE TRATAMENTO ATUAL		
	( )	Forma aguda da doença de Chagas
	( )	Forma indeterminada da doença de Chagas
	( )	Forma crônica cardíaca leve
	( )	Forma crônica digestiva leve
	( )	Paciente HIV+ com reativação
	( )	Paciente chagásico submetido a transplante ou terapia imunossupressiva
	( )	Paciente receptor de órgão de doador soropositivo para doença de Chagas
	( )	Acidente com material possivelmente contaminado
	( )	Protocolo de pesquisa clínica aprovado pelo Ministério da Saúde
	( )	Outros (especificar):

DADOS DO(S) MEDICAMENTO(S)					
( )	Benznidazol 100 mg comprimido	( )	Benznidazol 12,5 mg comprimido	( )	Nifurtimox 120 mg comprimido

1.4. Calcular a dose total prescrita (em mg) e o número de comprimidos. Datar e assinar o formulário.

Figura 4 – Cálculo da dose total

Dose total prescrita (mg):			
Número de comprimidos):			
Observações:			
ASSINATURA DO RESPONSÁVEL PELO PREENCHIMENTO			
DATA:		LOCAL:	
ASSINATURA DO RESPONSÁVEL PELO PREENCHIMENTO			

2. Anexar cópia do resultado positivo da Sorologia para a Doença de Chagas com duas metodologias diferentes.
3. Anexar cópia da ficha de notificação do E-SUS Notifica.
4. Prescrever o medicamento em receituário comum, em duas vias, com identificação do paciente, nome genérico do medicamento, posologia completa (consultar quadro 1 e Anexo B).

Quadro 1 – Regime posológico do tratamento antiparasitário com Benzonidazol.

**Posologia em Adultos:** 5 mg/kg/dia em uma a três doses, por 60 dias.
Para pacientes com mais de 60kg: O número de dias de tratamento pode ser estendido (até 80 dias)
**Dose máxima:** 300 mg/dia
Exemplo, uma pessoa com 70 kg poderá utilizar benzonidazol 300 mg/dia por 70 dias

5. Entregar a documentação ao paciente e encaminhá-lo à farmácia da Policlínica para dar entrada no processo de solicitação do medicamento.

FARMACÊUTICOS

1ª Dispensação

1. Conferir toda a documentação do paciente. Digitalizar a documentação e devolver ao paciente Receita e exames.
2. Encaminhar a documentação digitalizada para a Regional de Saúde de Divinópolis através do e-mail cesaf.div@saude.mg.gov.br.
3. Aguardar retorno do núcleo de assistência farmacêutica sobre a retirada do medicamento.
4. Solicitar carro para retirada do medicamento na CAF/SRS/Divinópolis.
5. Realizar o aceite da distribuição no SIGAF.
6. Comunicar ao paciente a chegada do medicamento na farmácia e agendar o atendimento. Caso não consiga contato com o paciente, solicitar a ESF busca ativa.
7. Orientar o paciente que leve a seguinte documentação: Receita, Documento com foto, CPF e comprovante de residência.
8. Registrar no SIGAF a dispensação do medicamento suficiente para 30 dias de tratamento e agendar a data de retorno para a retirada do restante do medicamento.
9. Dispensar o medicamento ao paciente e orientar sobre cuidados da administração e armazenamento, sinais de alerta de reação adversa e exames de monitoramento.
10. Solicitar que o paciente assine o recibo de dispensação em duas vias.
11. Fornecer o "Cartão do Benzonidazol" (Anexo C) e preencher as orientações de modo de uso junto com o paciente, realizando a orientação.
12. Orientar sobre a necessidade de apresentação do cartão em qualquer atendimento de saúde.
13. Orientar o paciente a comparecer a ESF de referência para acompanhamento multiprofissional do uso do medicamento.
14. Informar a ESF de referência que o paciente já está com o medicamento em mãos.

2ª Dispensação

1. Registrar no SIGAF a dispensação do medicamento suficiente para 30 dias de tratamento e para o caso de tratamentos com duração superior a este período, agendar novo retorno.
2. Dispensar o medicamento ao paciente e realizar uma consulta farmacêutica de monitoramento.
3. Registrar no sistema 'Crescer' todos os dados coletados no atendimento, conforme o passo a passo da figura 5.
4. Informar a ESF qualquer informação coletada na consulta farmacêutica que exija acompanhamento da equipe da ESF.
5. Registrar no cartão do BZN as informações necessárias.
6. Solicitar que o paciente assine o recibo de dispensação em duas vias.
7. Orientar o paciente sobre o modo de uso do medicamento.
8. . Orientar o paciente a manter o acompanhamento multiprofissional do uso do medicamento na ESF de referência.
9. Informar a ESF de referência que o paciente já está com o medicamento em mãos.

Figura 5: Registro de atentimento no sistema ‘Crescer’

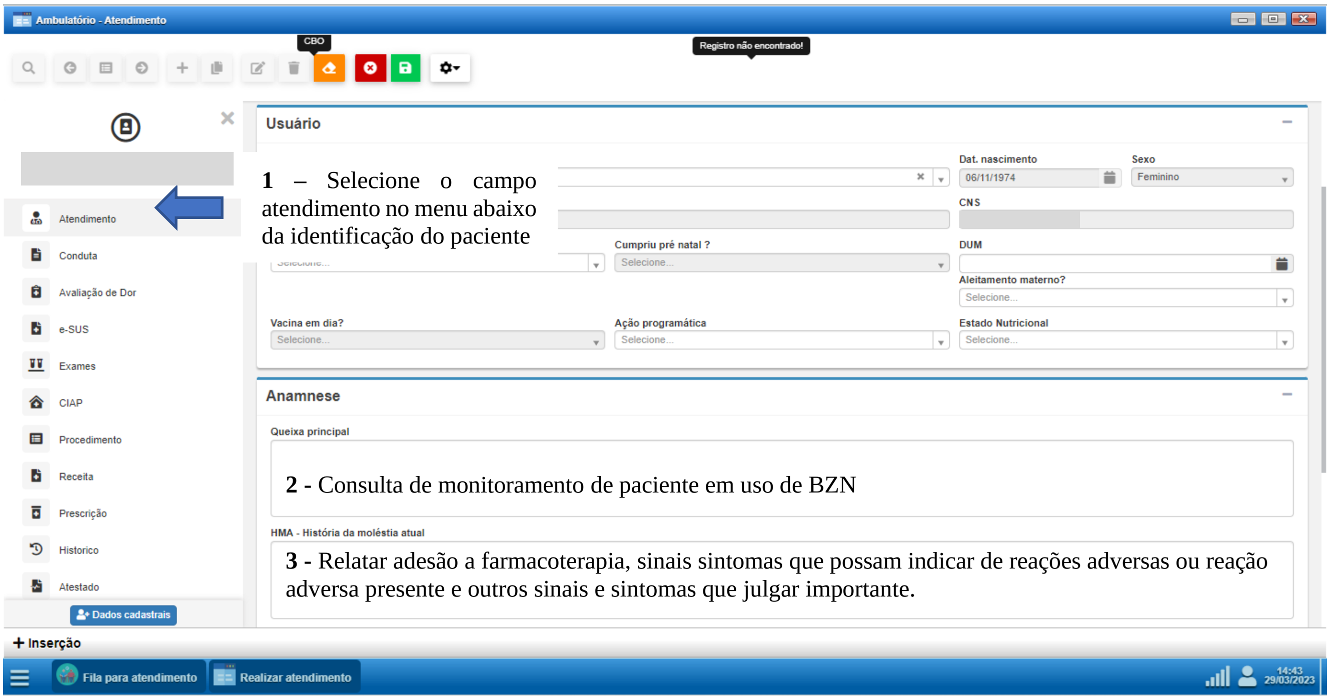

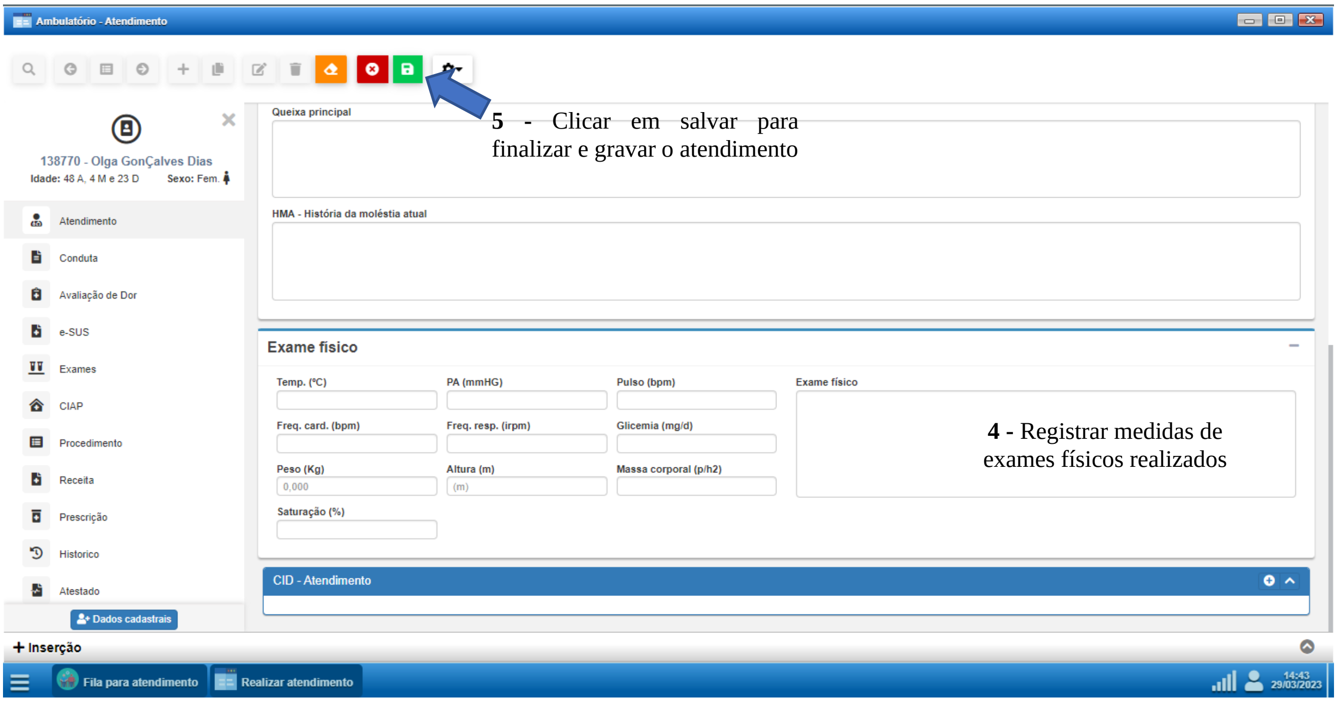

ANEXO A – MODELO DA FICHA DE SOLICITAÇÃO DE BENZONIDAZOL PARA TRATAMENTO DE PACIENTES COM DOENÇA DE CHAGAS

(https://drive.google.com/file/d/1vz9R6HaCw--wOnnMaPe4D52-5_6Qmove/view)

GOVERNO DO ESTADO DE MINAS GERAIS
SECRETARIA DE ESTADO DE SAÚDE

ANEXO - FORMULÁRIO DE SOLICITAÇÃO DE MEDICAMENTOS PARA TRATAMENTO DA DOENÇA DE CHAGAS
Para uso pelo Município/Estabelecimento de saúde.

**DADOS DO MÉDICO**			
Nome:		CPF:	
CRM:		CNS:	
Telefone fixo:		Telefone celular:	
**DADOS DO ESTABELECIMENTO DE SAÚDE SOLICITANTE**			
Nome do estabelecimento:		CNES:	
Endereço:		CEP:	
Município:		Telefone:	

**DADOS DO PACIENTE**								
Nome:								
RG:		CPF:		CNS:				
Data de nascimento:	_____/____/_______	Idade:		Sexo:	Feminino	( )	Masculino	( )
Nome da mãe:								
Endereço domiciliar:								
Município:				CEP:				
Telefone fixo:				Telefone celular:				

ANTECEDENTES CLÍNICOS (Descreva brevemente a história clínica do paciente com dados sobre forma clínica, local provável de infecção, exames realizados para confirmar o diagnóstico da doença de Chagas e laudo do eletrocardiograma)

INDICAÇÃO DE TRATAMENTO ATUAL

	( )	Forma aguda da doença de Chagas
	( )	Forma indeterminada da doença de Chagas
	( )	Forma crônica cardíaca leve
	( )	Forma crônica digestiva leve
	( )	Paciente HIV+ com reativação
	( )	Paciente chagásico submetido a transplante ou terapia imunossupressiva
	( )	Paciente receptor de órgão de doador soropositivo para doença de Chagas
	( )	Acidente com material possivelmente contaminado
	( )	Protocolo de pesquisa clínica aprovado pelo Ministério da Saúde
	( )	Outros (especificar):

DADOS DO(S) MEDICAMENTO(S)

( )	Benznidazol 100 mg comprimido	( )	Benznidazol 12,5 mg comprimido	( )	Nifurtimox 120 mg comprimido
Em caso de solicitação de Nifurtimox descreve o motivo:					

Dose total prescrita (mg):			
Número de comprimidos):			
Observações:			
**ASSINATURA DO RESPONSÁVEL PELO PREENCHIMENTO**			
DATA:		LOCAL:	

____
ASSINATURA DO RESPONSÁVEL PELO PREENCHIMENTO

ANEXO B – MODELO DE RECEITUÁRIO

RECEITUÁRIO

Paciente: José da Silva

Uso oral

Benzonidazol 100mg ____________________ 180 comprimidos

Tomar 2 comprimidos pela manhã e 1 comprimido a noite

Durante 60 dias

Carimbo e Assinatura do Médico

ANEXO C – CARTÃO DE USO DO BENZONIDAZOL

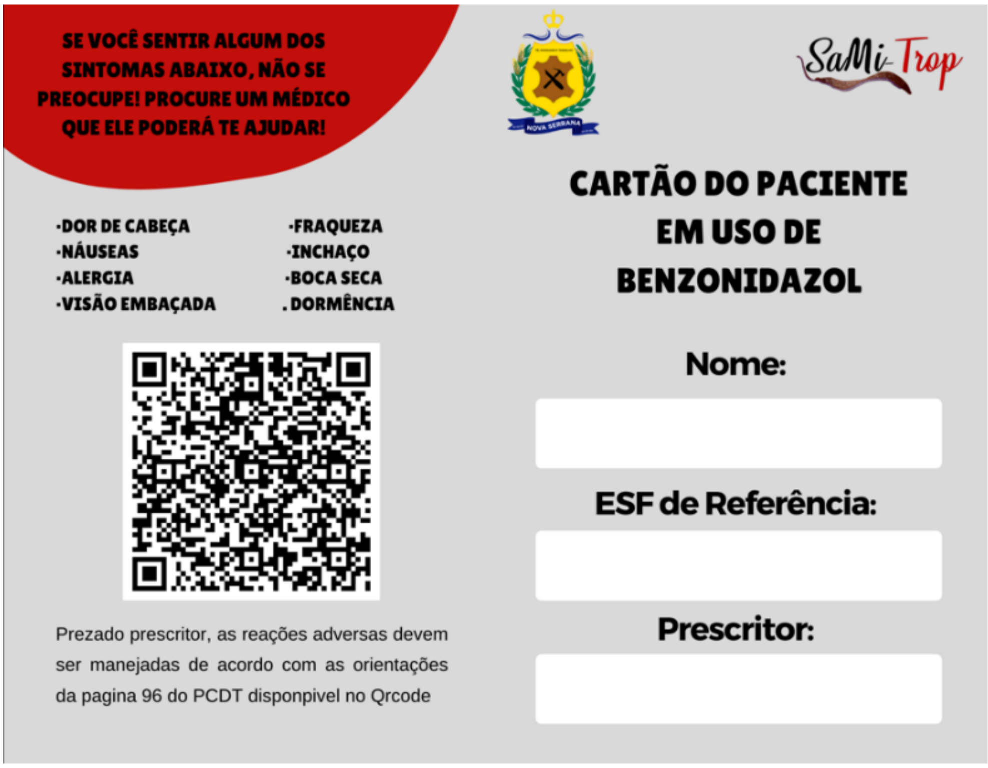

SE VOCÊ SENTIR ALGUM DOS SINTOMAS ABAIXO, NÃO SE PREOCUPE! PROCURE UM MÉDICO QUE ELE PODERÁ TE AJUDAR!

- DOR DE CABEÇA
- NÁUSEAS
- ALERGIA
- VISÃO EMBAÇADA
- FRAQUEZA
- INCHAÇO
- BOCA SECA
- DORMÊNCIA

Prezado prescritor, as reações adversas devem ser manejadas de acordo com as orientações da pagina 96 do PCDT disponpivel no Qrcode

SaMi-Trop

CARTÃO DO PACIENTE EM USO DE BENZONIDAZOL

Nome:

ESF de Referência:

Prescritor:

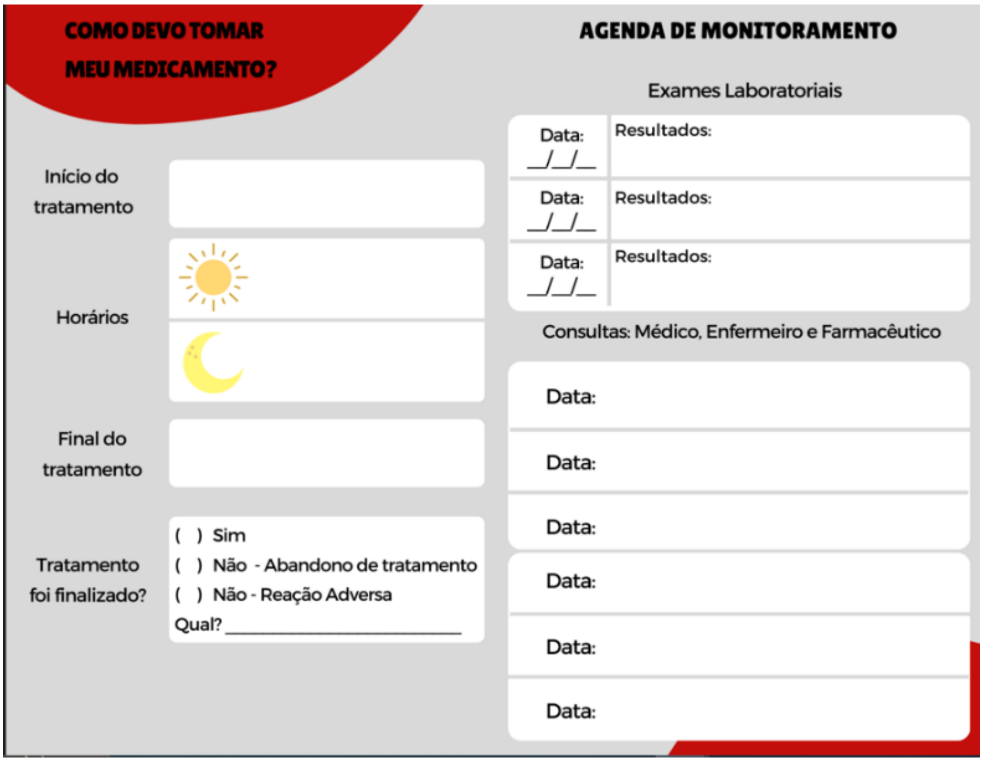

COMO DEVO TOMAR MEU MEDICAMENTO?

Início do tratamento

Horários

Final do tratamento

Tratamento foi finalizado?
( ) Sim
( ) Não - Abandono de tratamento
( ) Não - Reação Adversa
Qual? ______________________

AGENDA DE MONITORAMENTO

Exames Laboratoriais

Data: __/__/__	Resultados:
Data: __/__/__	Resultados:
Data: __/__/__	Resultados:

Consultas: Médico, Enfermeiro e Farmacêutico

Data:

Data:

Data:

Data:

Data:

Data:

PROCEDIMENTO OPERACIONAL PADRÃO – POP Nº 3<br>PRESCRIÇÃO E DISPENSAÇÃO DO BENZONIDAZOL			
Data de Emissão<br><br>__/__/___	Data de Vigência<br><br>__/__/___ a __/__/___	Próxima Revisão<br><br>__/__/___	Versão nº 1

**Atividade:** Prescrição, Solicitação e Dispensação do Benzonidazol e Acompanhamento do Tratamento de Pacientes com Doença de Chagas.<br>**Executante:** Médico e Farmacêutico.
**Resultado esperado:** Padronizar a solicitação do Benzonidazol para tratamento de pacientes com doença de Chagas no município C
**Materiais Necessários:** Caneta, formulário de solicitação do Benzonidazol, prescrição médica, resultado da sorologia para doença de Chagas, carimbo do médico, ficha de notificação do E-SUS Notifica, computador com acesso à internet, impressora, papel.
**Referências:** Brasil. Ministério da Saúde, 2018. BRASIL. Protocolo Clínico e Diretrizes Terapêuticas Doença de Chagas. Brasília: Ministério da Saúde.

MÉDICOS

1. Preencher todos os campos do Formulário de Solicitação do Benzonidazol para o Tratamento de Pacientes com Doença de Chagas (Anexo A)
   1.1. Identificação profissional e identificação do paciente.

Figura 1 – Identificação do profissional e do paciente

GOVERNO DO ESTADO DE MINAS GERAIS
SECRETARIA DE ESTADO DE SAÚDE

ANEXO - FORMULÁRIO DE SOLICITAÇÃO DE MEDICAMENTOS PARA TRATAMENTO DA DOENÇA DE CHAGAS
Para uso pelo Município/Estabelecimento de saúde.

DADOS DO MÉDICO			
Nome:		CPF:	
CRM:		CNS:	
Telefone fixo:		Telefone celular:	

DADOS DO ESTABELECIMENTO DE SAÚDE SOLICITANTE			
Nome do estabelecimento:		CNES:	
Endereço:		CEP:	
Município:		Telefone:	

DADOS DO PACIENTE								
Nome:								
RG:		CPF:		CNS:				
Data de nascimento:	______/____/______	Idade:		Sexo:	Feminino	( )	Masculino	( )
Nome da mãe:								
Endereço domiciliar:								
Município:				CEP:				
Telefone fixo:				Telefone celular:				

1.2. Descrever brevemente a história clínica do paciente com dados sobre a forma clínica, local provável de infecção, exames realizados para confirmar o diagnóstico da doença de chagas e o laudo do eletrocardiograma.

Figura 2 – Antecedentes Clínicos

ANTECEDENTES CLÍNICOS (Descreva brevemente a história clínica do paciente com dados sobre forma clínica, local provável de infecção, exames realizados para confirmar o diagnóstico da doença de Chagas e laudo do eletrocardiograma)

1.3. Registrar qual a indicação do tratamento e medicamento prescrito, conforme Protocolo Clínico e Diretrizes Terapêuticas.

Figura 3 – Indicação do tratamento antiparasitário

INDICAÇÃO DE TRATAMENTO ATUAL		
	( )	Forma aguda da doença de Chagas
	( )	Forma indeterminada da doença de Chagas
	( )	Forma crônica cardíaca leve
	( )	Forma crônica digestiva leve
	( )	Paciente HIV+ com reativação
	( )	Paciente chagásico submetido a transplante ou terapia imunossupressiva
	( )	Paciente receptor de órgão de doador soropositivo para doença de Chagas
	( )	Acidente com material possivelmente contaminado
	( )	Protocolo de pesquisa clínica aprovado pelo Ministério da Saúde
	( )	Outros (especificar):

DADOS DO(S) MEDICAMENTO(S)					
( )	Benznidazol 100 mg comprimido	( )	Benznidazol 12,5 mg comprimido	( )	Nifurtimox 120 mg comprimido

1.4. Calcular a dose total prescrita (em mg) e o número de comprimidos. Datar e assinar o formulário.

Figura 4 – Cálculo da dose total

Dose total prescrita (mg):			
Número de comprimidos):			
Observações:			
ASSINATURA DO RESPONSÁVEL PELO PREENCHIMENTO			
DATA:		LOCAL:	
ASSINATURA DO RESPONSÁVEL PELO PREENCHIMENTO			

2. Anexar cópia do resultado positivo da Sorologia para a Doença de Chagas com duas metodologias diferentes.
3. Anexar cópia da ficha de notificação do E-SUS Notifica.
4. Prescrever o medicamento em receituário comum, em duas vias, com identificação do paciente, nome genérico do medicamento, posologia completa (consultar quadro 1 e Anexo B).

Quadro 1 – Regime posológico do tratamento antiparasitário com Benzonidazol.

**Posologia em Adultos:** 5 mg/kg/dia em uma a três doses, por 60 dias.
Para pacientes com mais de 60kg: O número de dias de tratamento pode ser estendido (até 80 dias)
**Dose máxima:** 300 mg/dia
Exemplo, uma pessoa com 70 kg poderá utilizar benzonidazol 300 mg/dia por 70 dias

5. Entregar a documentação ao paciente e encaminhá-lo à farmácia da Policlínica para dar entrada no processo de solicitação do medicamento.

FARMACÊUTICOS

1ª Dispensação

1. Conferir toda a documentação do paciente. Digitalizar a documentação e devolver ao paciente Receita e exames.
2. Encaminhar a documentação digitalizada para a Regional de Saúde de Divinópolis através do e-mail cesaf.div@saude.mg.gov.br.
3. Aguardar retorno do núcleo de assistência farmacêutica sobre a retirada do medicamento.
4. Solicitar carro para retirada do medicamento na CAF/SRS/Divinópolis.
5. Realizar o aceite da distribuição no SIGAF.
6. Comunicar ao paciente a chegada do medicamento na farmácia e agendar o atendimento. Caso não consiga contato com o paciente, solicitar a ESF busca ativa.
7. Orientar o paciente que leve a seguinte documentação: Receita, Documento com foto, CPF e comprovante de residência.
8. Registrar no SIGAF a dispensação do medicamento suficiente para 30 dias de tratamento e agendar a data de retorno para a retirada do restante do medicamento.
9. Dispensar o medicamento ao paciente e orientar sobre cuidados da administração e armazenamento, sinais de alerta de reação adversa e exames de monitoramento.
10. Solicitar que o paciente assine o recibo de dispensação em duas vias.
11. Fornecer o "Cartão do Benzonidazol" (Anexo C) e preencher as orientações de modo de uso junto com o paciente, realizando a orientação.
12. Orientar sobre a necessidade de apresentação do cartão em qualquer atendimento de saúde.
13. Orientar o paciente a comparecer a ESF de referência para acompanhamento multiprofissional do uso do medicamento.
14. Informar a ESF de referência que o paciente já está com o medicamento em mãos.

2ª Dispensação

1. Registrar no SIGAF a dispensação do medicamento suficiente para 30 dias de tratamento e para o caso de tratamentos com duração superior a este período, agendar novo retorno.
2. Dispensar o medicamento ao paciente e realizar uma consulta farmacêutica de monitoramento.
3. Registrar no sistema 'Crescer' todos os dados coletados no atendimento, conforme o passo a passo da figura 5.
4. Informar a ESF qualquer informação coletada na consulta farmacêutica que exija acompanhamento da equipe da ESF.
5. Registrar no cartão do BZN as informações necessárias.
6. Solicitar que o paciente assine o recibo de dispensação em duas vias.
7. Orientar o paciente sobre o modo de uso do medicamento.
8. . Orientar o paciente a manter o acompanhamento multiprofissional do uso do medicamento na ESF de referência.
9. Informar a ESF de referência que o paciente já está com o medicamento em mãos.

Figura 5: Registro de atentimento no sistema ‘Crescer’

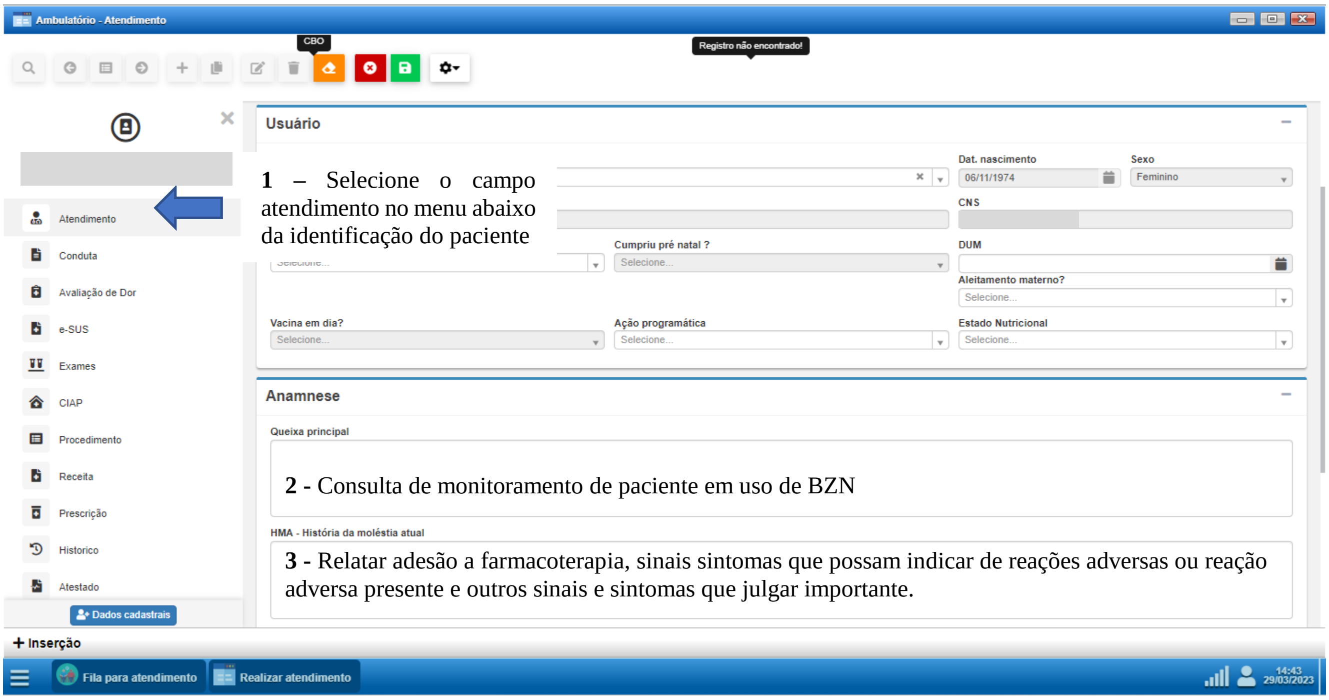

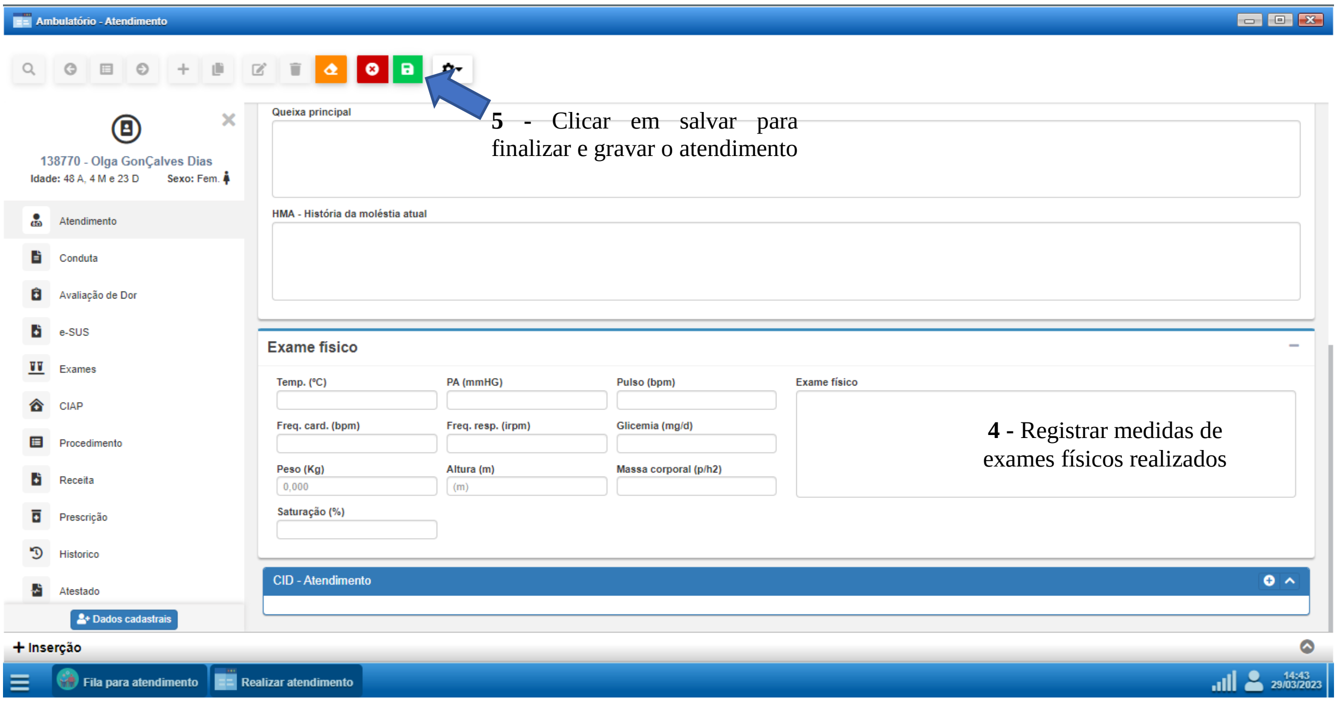

ANEXO A – MODELO DA FICHA DE SOLICITAÇÃO DE BENZONIDAZOL PARA TRATAMENTO DE PACIENTES COM DOENÇA DE CHAGAS

(https://drive.google.com/file/d/1vz9R6HaCw--wOnnMaPe4D52-5_6Qmove/view)

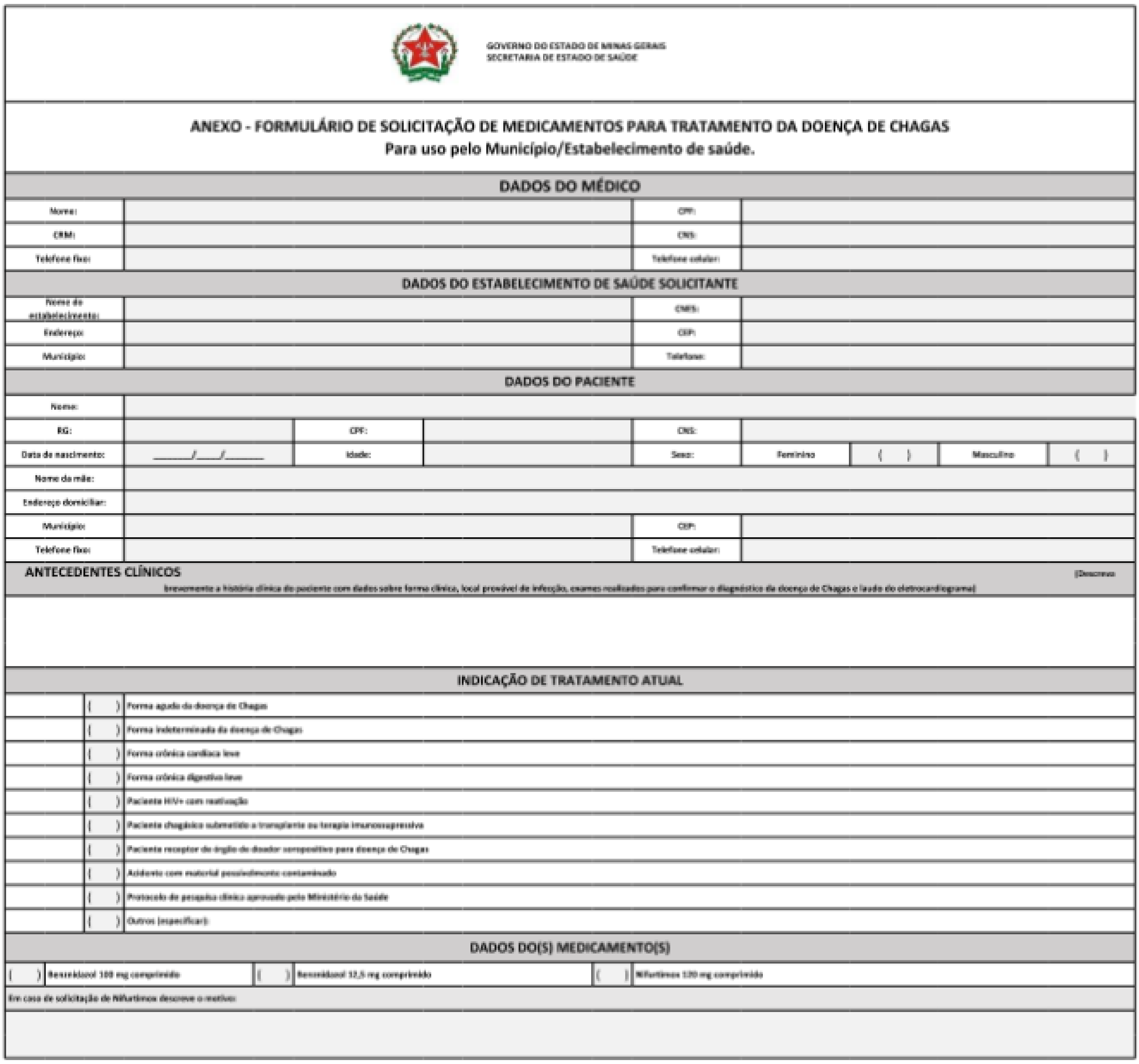

GOVERNO DO ESTADO DE MINAS GERAIS
SECRETARIA DE ESTADO DE SAÚDE

ANEXO - FORMULÁRIO DE SOLICITAÇÃO DE MEDICAMENTOS PARA TRATAMENTO DA DOENÇA DE CHAGAS
Para uso pelo Município/Estabelecimento de saúde.

DADOS DO MÉDICO

Nome:		CPF:	
CRM:		CNS:	
Telefone fixo:		Telefone celular:	

DADOS DO ESTABELECIMENTO DE SAÚDE SOLICITANTE

Nome do estabelecimento:		CNES:	
Endereço:		CEP:	
Município:		Telefone:	

DADOS DO PACIENTE

Nome:									
RG:		CPF:		CNS:					
Data de nascimento:	_____/____/_____	Idade:		Sexo:	Feminino	( )	Masculino	( )	
Nome da mãe:									
Endereço domiciliar:									
Município:				CEP:					
Telefone fixo:				Telefone celular:					

ANTECEDENTES CLÍNICOS (Descreva brevemente a história clínica do paciente com dados sobre forma clínica, local provável de infecção, exames realizados para confirmar o diagnóstico da doença de Chagas e laudo do eletrocardiograma)

INDICAÇÃO DE TRATAMENTO ATUAL

	( )	Forma aguda da doença de Chagas
	( )	Forma indeterminada da doença de Chagas
	( )	Forma crônica cardíaca leve
	( )	Forma crônica digestiva leve
	( )	Paciente HIV+ com reativação
	( )	Paciente chagásico submetido a transplante ou terapia imunossupressiva
	( )	Paciente receptor de órgão de doador soropositivo para doença de Chagas
	( )	Acidente com material possivelmente contaminado
	( )	Protocolo de pesquisa clínica aprovado pelo Ministério da Saúde
	( )	Outros (especificar):

DADOS DO(S) MEDICAMENTO(S)

( )	Benznidazol 100 mg comprimido	( )	Benznidazol 12,5 mg comprimido	( )	Nifurtimox 120 mg comprimido

Em caso de solicitação de Nifurtimox descreva o motivo:

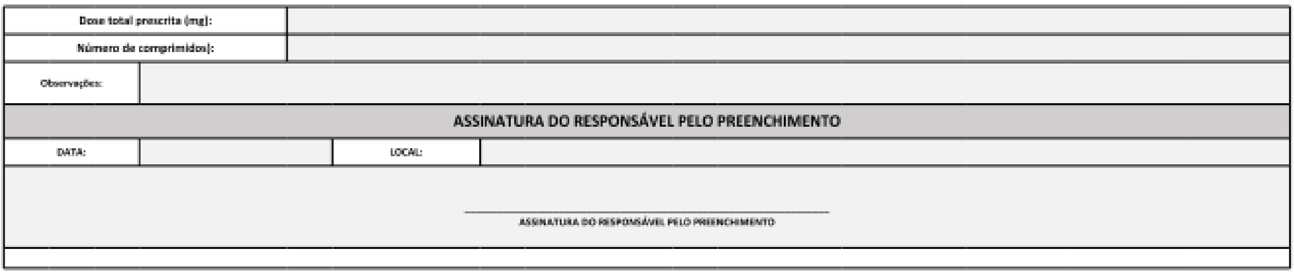

Dose total prescrita (mg):	
Número de comprimidos):	
Observações:	

ASSINATURA DO RESPONSÁVEL PELO PREENCHIMENTO

DATA:		LOCAL:	

____
ASSINATURA DO RESPONSÁVEL PELO PREENCHIMENTO

IMPLEMENTA SaMi-Trop

ANEXO B – MODELO DE RECEITUÁRIO

RECEITUÁRIO

Paciente: José da Silva

Uso oral

Benzonidazol 100mg ____________________ 180 comprimidos

Tomar 2 comprimidos pela manhã e 1 comprimido a noite

Durante 60 dias

____

Carimbo e Assinatura do Médico

ANEXO C – CARTÃO DE USO DO BENZONIDAZOL

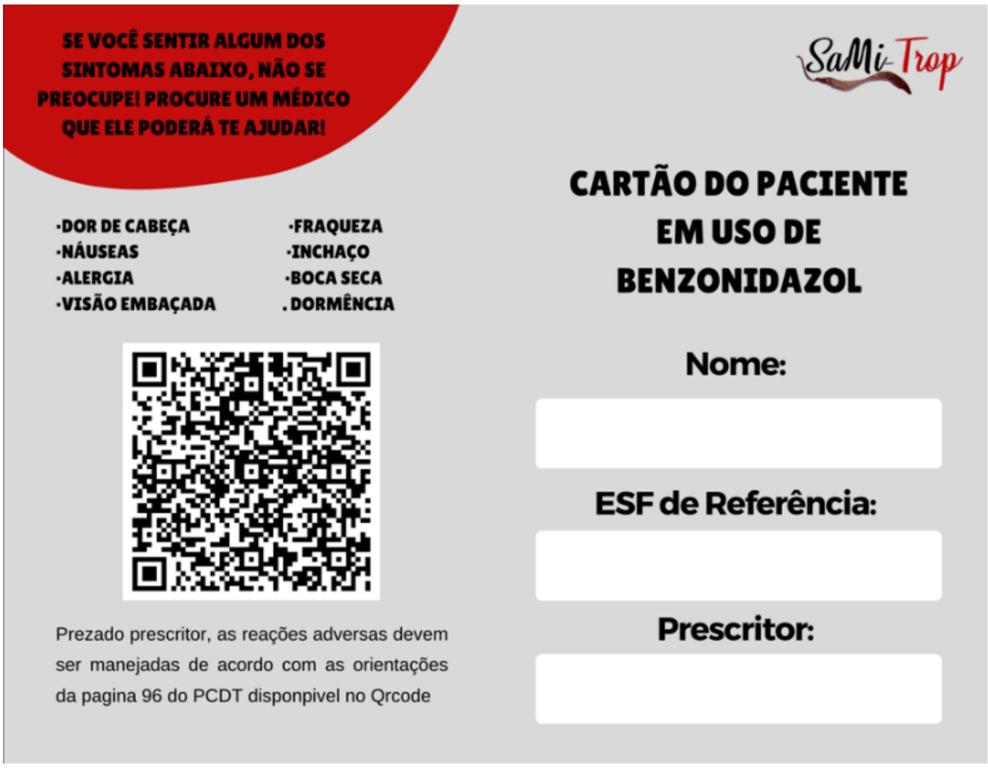

SE VOCÊ SENTIR ALGUM DOS SINTOMAS ABAIXO, NÃO SE PREOCUPE! PROCURE UM MÉDICO QUE ELE PODERÁ TE AJUDAR!

SaMi-Trop

CARTÃO DO PACIENTE EM USO DE BENZONIDAZOL

- DOR DE CABEÇA
- NÁUSEAS
- ALERGIA
- VISÃO EMBAÇADA
- FRAQUEZA
- INCHAÇO
- BOCA SECA
- DORMÊNCIA

Nome:

ESF de Referência:

Prescritor:

Prezado prescritor, as reações adversas devem ser manejadas de acordo com as orientações da pagina 96 do PCDT disponpivel no Qrcode

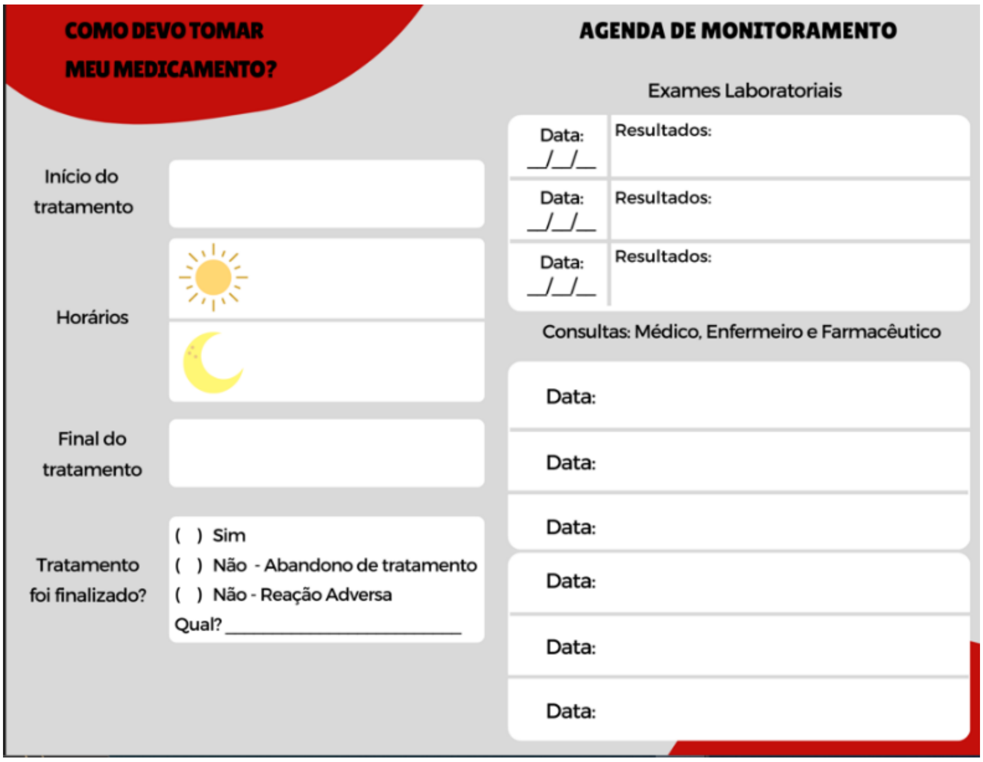

COMO DEVO TOMAR MEU MEDICAMENTO?

Início do tratamento

Horários

Final do tratamento

Tratamento foi finalizado?
( ) Sim
( ) Não - Abandono de tratamento
( ) Não - Reação Adversa
Qual? ______________________

AGENDA DE MONITORAMENTO

Exames Laboratoriais

Data: _/_/_	Resultados:
Data: _/_/_	Resultados:
Data: _/_/_	Resultados:

Consultas: Médico, Enfermeiro e Farmacêutico

Data:

Data:

Data:

Data:

Data:

Data:

ORIENTAÇÕES PARA VIGILÂNCIA ENTOMOLÓGICA DA DOENÇA DE CHAGAS

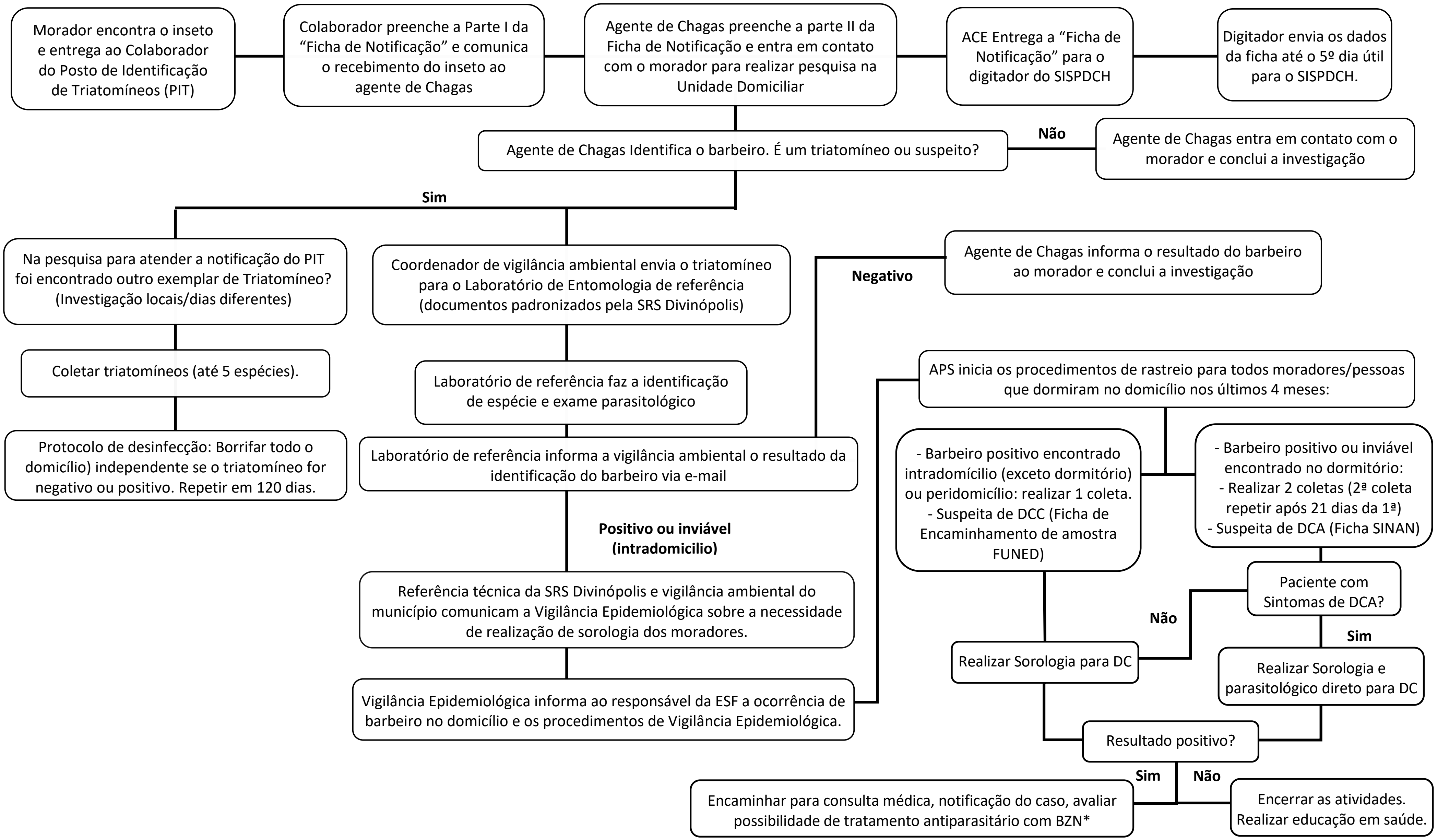

*Consultar fluxo da APS

Nº DO PIT:	LOCALIDADE	CATEGORIA	RG	COLABORADOR VOLUNTARIO	PROFISSÃO	ENDEREÇO	SUBSTITUTO
9580	NOVA SERRANA	CIDADE	29	KATIUSCIA F. FERREIRA	ENFERMEIRA	SMS 2112 N.PIT 9580	IDALIA CARNEIRO
9631	NOVAIS	POVOADO	1	JOANA REGINA S. AZEVEDO	ACS	FNS 23 N. PIT 9631	ENFERMEIRA TÁSSIA
9632	MORRO DO CHAPÉU	FAZENDA	2	LIBÉRIO F. FERNANDES	LAVRADOR	FNS 32 N. PIT 9632	ELENICE APARECIDA
9633	AREIAS	POVOADO	3	ORCEIA FREITAS FERNANDES	ACS	FNS 03 N.PIT 9633	ENFERMEIRA TÁSSIA
9634	BOA VISTA	POVOADO	6	FERNANDO DIVINO MARTINS	TÉC. ENF	P. SAUDE 82 N. PIT 9634	ENFERMEIRA CAMILA PACHECO
9635	CASINHA DO CAMPO	FAZENDA	9	JOÃO BATISTA DE FARIA	LAVRADOR	FNS 24 N. PIT 9635	NÃO TEM
9636	ÁGUA ESPRAIADA	FAZENDA	11	ADILMA DE OLIVEIRA SANTOS	DO LAR	FNS 51 N. PIT 9636	NÃO TEM
9637	MOREIRAS	POVOADO	13	ELAINE MARIA DOS SANTOS	ACS	FNS 218 N. PIT 9637	ENFERMEIRA CAMILA GONDIM
9638	GAMAS	POVOADO	15	CAMILA BRUNA GONDIM GOMIDES	ENFERMEIRO	FNS 299 N.PIT 9638	ENFERMEIRA CAMILA GONDIM
9639	BARRETOS	POVOADO	17	SOLIANE CONCEIÇÃO PEREIRA	RECEPCIONISTA	FNS 239 N. PIT 9639	AUX. ENF. VALÉRIA
9640	RIPAS	POVOADO	18	JOÃO MAXIMO DE ANDRADE	AUX. ENF.	P. SAUDE 123 N. PIT 9640	ENFERMEIRA CAMILA GONDIM
9641	FAZENDA PARANÁ	FAZENDA	89	ADRIANA SILVA TORRES	ACS	FNS 92 - N. DO PIT 9982	ENFERMEIRA LARISSA
9642	HENRIQUE II	FAZENDA	22	FABIO JOSE FARIA DA SILVA	LAVRADOR	FNS 20 N.PIT 9642	NÃO TEM
9642	CAPÃO	POVOADO	71	ROZIMEIRE RIBEIRO DA SILVA SANTOS	ACS	FNS 604 N.PIT 9986	ENFERMEIRA ANDREIA
9985	CIDADE NOVA	CIDADE	98	EDNA PEREIRA DA SILVA	RECEPCIONISTA	FNS 15 N. PIT 9985	ENFERMEIRO LUCAS

FICHA DE IDENTIFICAÇÃO DE FATOR DE RISCO PARA DOENÇA DE CHAGAS

**Identificação da Família:**		**Unidade de Referência:**					**Data:**
	**Membro da família**	Mora/morou em locais onde tem barbeiro da Doença de Chagas ou já morou próximo a estes locais.	Mora ou morou em casas de taipa, sapê, pau-a-pique, madeira ou barro.	Recebeu transfusão de sangue antes de 1992	Foi picado pelo barbeiro da doença de Chagas (Chupão, Fincão, Chupança)	Têm familiares ou mora/já morou com alguém que tenha diagnóstico de doença de Chagas.	Mora/morou em região hiper endêmicas (Norte de Minas Gerais e Vale do Jequitinhonha, norte e nordeste do país, e América Latina) *
**1**							
**2**							
**3**							
**4**							
**5**							
**6**							
**7**							
**8**							
**9**							
**10**							
**11**							
**12**							
**Responsável pelo fornecimento das informações:**		**Responsável pelo preenchimento da ficha:**					

NORTE DE MINAS

BERIZAL
BOCAIÚVA
BOTUMIRIM
CAPITÃO ENÉAS
CATUTI
CLARO DOS POÇÕES
CORAÇÃO DE JESUS
CRISTÁLIA
CURRAL DE DENTRO
ENGENHEIRO NAVARRO
ESPINOSA
FRANCISCO DUMONT
FRANCISCO SÁ
FRUTA DE LEITE
GAMELEIRAS
GLAUCILÂNDIA
GRÃO MOGOL
GUARACIAMA
INDAIABIRA
ITACAMBIRA
JAÍBA
JANAÚBA
JEQUITAÍ
JOAQUIM FELÍCIO
JOSENÓPOLIS
JURAMENTO
LAGOA DOS PATOS
MAMONAS
MATIAS CARDOSO
MATO VERDE
MIRABELA
MONTE AZUL
MONTES CLAROS
MONTEZUMA
NINHEIRA
NOVA PORTEIRINHA
NOVORIZONTE
OLHOS-D'ÁGUA
PADRE CARVALHO
PAI PEDRO
PORTEIRINHA
RIACHO DOS MACHADOS
RIO PARDO DE MINAS
RUBELITA
SALINAS
SANTA CRUZ DE SALINAS
SANTO ANTÔNIO DO RETIRO
SÃO JOÃO DA LAGOA
SÃO JOÃO DO PACUÍ
SÃO JOÃO DO PARAÍSO
SERRANÓPOLIS DE MINAS
TAIOBEIRAS
VARGEM GRANDE DO RIO PARDO
VERDELÂNDIA

VALE DO JEQUITINHONHA

ALVORADA DE MINAS
ANGELÂNDIA
ARICANDUVA
CAPELINHA
CARBONITA
COLUNA
COUTO MAGALHÃES DE MINAS
DATAS, DIAMANTINA
FELÍCIO DOS SANTOS
GOUVEIA
ITAMARANDIBA
LEME DO PRADO
MINAS NOVAS
PRESIDENTE KUBISTSCHEK
RIO VERMELHO
SÃO GONÇALO DO RIO PRETO
SENADOR MODESTINO GONÇALVES
SERRA AZUL DE MINAS
SERRO
TURMALINA
VEREDINHA
ARAÇUAÍ
BERILO
CARAÍ
CHAPADA DO NORTE
COMERCINHO
CORONEL MURTA
FRANCISCO BADARÓ
ITAOBIM
ITINGA
JENIPAPO DE MINAS
JOSÉ GONÇALVES DE MINAS
MEDINA
PADRE PARAÍSO
PONTO DOS VOLANTES
VIRGEM DA LAPA
ALMENARA, BANDEIRA
CACHOEIRA DO PAJEÚ
DIVISÓPOLIS
FELISBURGO
JACINTO
JEQUITINHONHA
JOAÍMA
JORDÂNIA
MATA VERDE
PALMÓPOLIS
PEDRA AZUL
RIO DO PRADO
RUBIM, SALTO DA DIVISA
SANTA MARIA DO SALTO
SANTO ANTONIO DO JACINTO

AMÉRICA LATINA

PRINCIPALMENTE, ARGENTINA, BOLÍVIA, COLÔMBIA, GUIANAS, PERU E VENEZUELA

IMPLEMENTA SaMi-Trop

CASO SUSPEITO DE CHAGAS CRÔNICA

- Mora/morou em locais onde tem barbeiro da Doença de Chagas ou já morou próximo a estes locais.
- Mora ou morou em casas de taipa, sapê, pau-a-pique, madeira ou barro.
- Recebeu transfusão de sangue antes de 1992.
- Foi picado pelo barbeiro da doença de Chagas (Chupão, Fincão, Chupança).
- Têm familiares ou mora/já morou com alguém que tenha diagnóstico de doença de Chagas.
- Mora/morou em região hiper endêmicas (Norte de Minas Gerais e Vale do Jequitinhonha, norte e nordeste do país, Argentina, Bolívia, Colômbia, Guianas, Peru e Venezuela).

CRITÉRIOS PARA RASTREIO: AO MENOS UMA RESPOSTA AFIRMATIVA

Médico/Enfermeiro solicita sorologia para DC por 2 métodos distintos (IGg – Eliza, IFI, HAI) e preenche a Ficha de Encaminhamento de Amostra (FUNED) e encaminha o paciente para a marcação do exame na recepção da ESF.

Recepção da ESF retém o pedido do exame e Ficha de Encaminhamento de Amostra (FUNED) e encaminha ao laboratório e entrega somente a confirmação de agendamento ao paciente (Agendar somente 2 dias após a data da marcação em função do fluxo da rota)

Paciente compareceu para a coleta do exame na data agendada?

Não: Laboratório comunica ausência do paciente a ESF de origem. ESF realiza Busca Ativa.

Sim: Laboratório cadastra amostra no GAL e envia a amostra à FUNED

Vigilância monitora os resultados no GAL e informa os resultados para a ESF de origem

Se resultado positivo, ESF agenda consulta e notifica o caso (Ficha de Notificação de DC Crônica)

Na avaliação médica:
- Investigar sintomas de forma digestiva.
- Solicitar ECG e RX de tórax

Megacólon e Megaesôfago. Sinais: acalasia, amiloidose, sarcoidose, neurofibromatose, gastroenterite eosinofílica, coinfecção por *H. pylori*, refluxo, constipação e tumores.
SE SOROLOGIA POSITIVA + SINTOMAS: ENCAMINHAR PARA O GASTRO

ECG sem alterações

Forma Indeterminada - Elegível para tratamento com Benzonidazol (BZN).

Prescrição do medicamento: receituário comum + formulário de solicitação do medicamento + cópia da notificação no SINAN + cópia do resultado da sorologia.

Encaminhar o paciente até a farmácia da policlínica para a entrada no processo de solicitação do medicamento.

Farmácia da Policlínica encaminha a documentação para a SRS Divinópolis para liberação do medicamento.

Farmácia da Policlínica informa ao paciente que o medicamento já está disponível e realiza a dispensação para 30 dias de tratamento e fornece o 'Cartão do Benzonidazol'.

Acompanhamento multidisciplinar do tratamento farmacológico:* consulta médica + monitoramento de exames laboratoriais (Hemograma e Função Hepática e renal) + visitas do ACS + consulta farmacêutica + consulta enfermagem.

Relatório médico de finalização do tratamento com BZN

Acompanhamento anual na APS (consulta médica + ECG)

ECG com alterações menores

Solicitar ECO Via regulação para avaliação da elegibilidade para tratamento antiparasitário.

Elegível para tratamento antiparasitário? (Elegível = Fração de Ejeção > 40%, sem insuficiência cardíaca e arritmias graves)

Sim: Prescrição do medicamento (ver acima)

Não: Encaminhar para acompanhamento com cardiologista via regulação

ECG com alterações maiores

Encaminhar para acompanhamento com cardiologista via regulação com ficha de encaminhamento com histórico detalhado do caso.

Regulação informa ao ESF sobre a consulta e a ESF comunica ao paciente.

Paciente passa por avaliação cardiológica e é contra referenciado para APS com Plano Cuidado compartilhado na Atenção Primária e Policlínica de acordo com a complexidade do caso.

LEGENDA:
- Rastreio
- Vigilância
- Seguimento Atenção Primária
- Seguimento na Policlínica

*Em caso de Reação Adversa, consultar a conduta no Qrcode no Cartão do BZN
