# Supplementary material for: Implementation Science in the Development of a Care Pathway for Chronic Chagas Disease: An Experience from a Municipality in Minas Gerais
Source: Rev Soc Bras Med Trop. 2026 Feb 9;59:e0381-2025. doi: 10.1590/0037-8682-0381-2025 (PMC12892926; doi:10.1590/0037-8682-0381-2025)
Supplement: Supplementary material 2 [file 1678-9849-rsbmt-59-e0381-2025-md2.pdf]

CHAGAS TEM TRATAMENTO

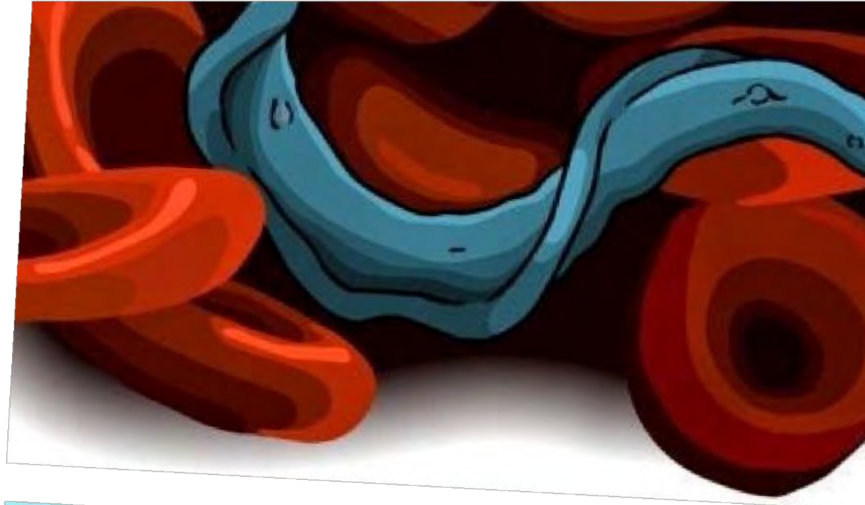

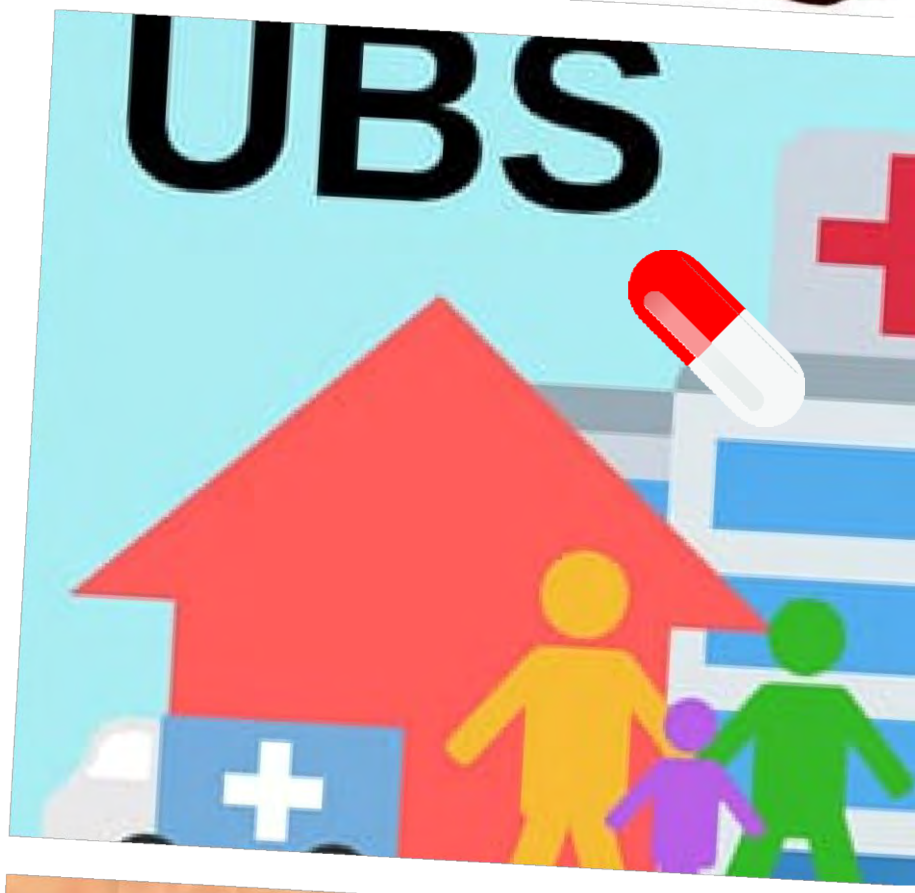

O tratamento é disponibilizado pelo SUS.

Se não tratada a doença, pode afetar gravemente o coração, esôfago e intestino.

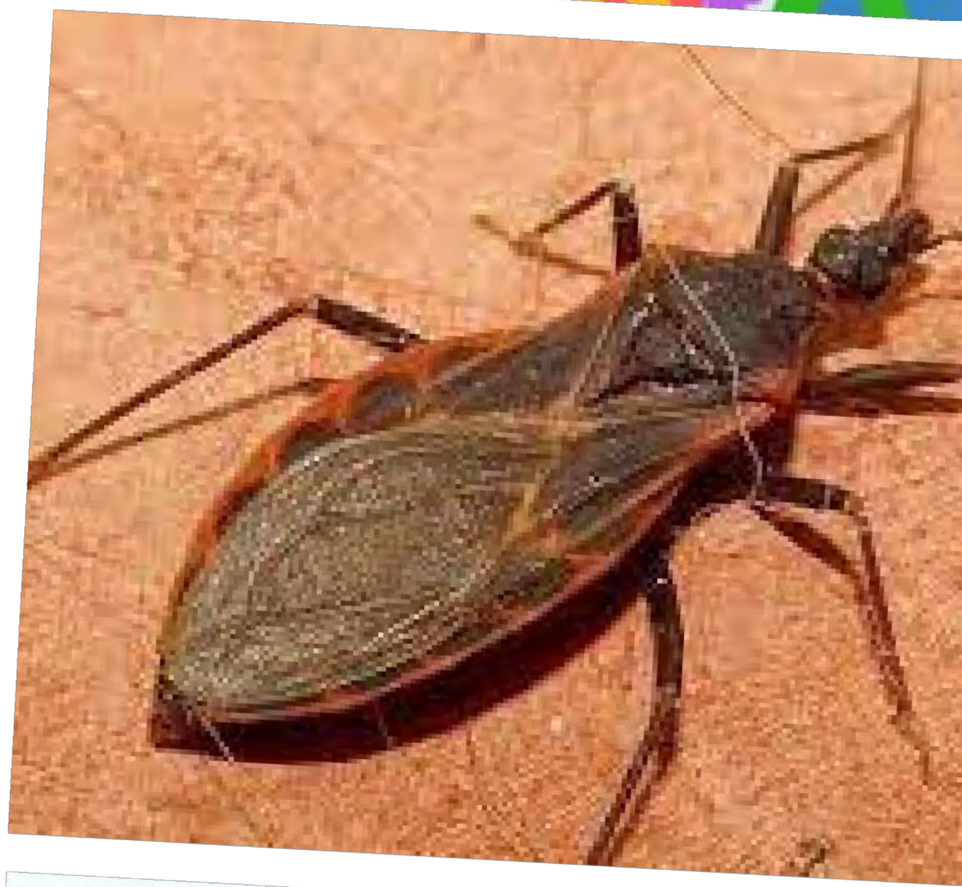

O tratamento deve ser iniciado o mais rápido possível. Não deixe para depois!

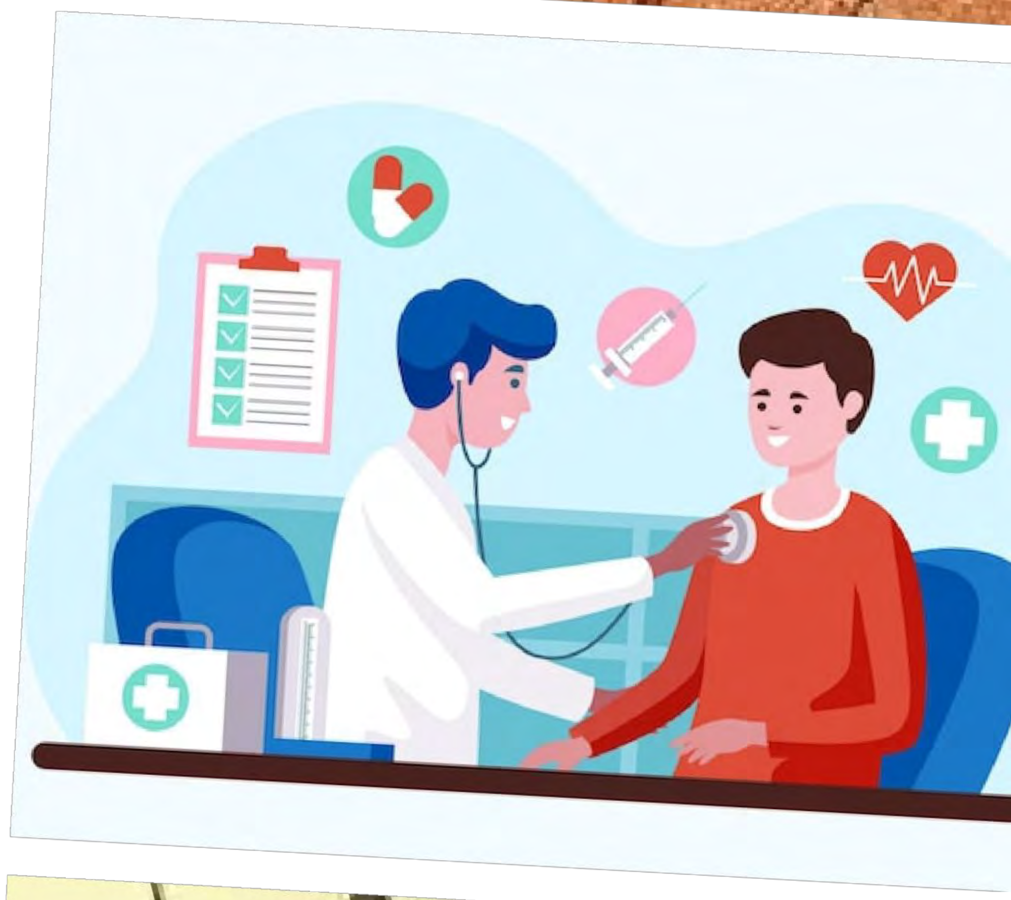

Procure agora um médico!

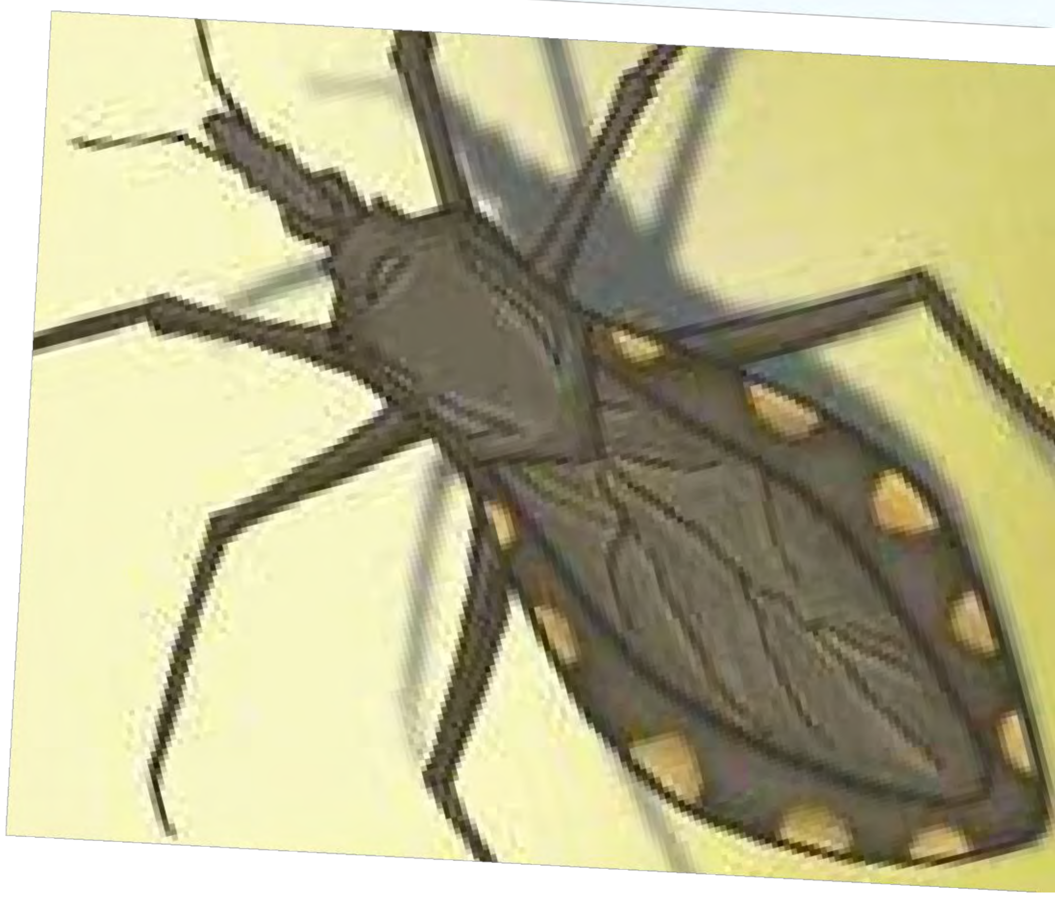

Não interrompa o tratamento. Em caso de efeito colateral, procure um médico.

Unimontes
Universidade Estadual de Montes Claros

DOENÇA DE CHAGAS

FATORES DE RISCO

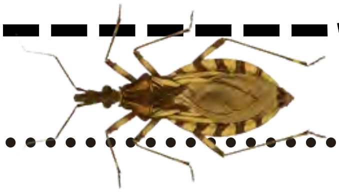

- [ ] Mora/morou em locais onde tem barbeiro ou próximo a locais onde tem.
- [ ] Mora/morou em casa de madeira, pau a pique, estuque, taipa, sapê ou de barro.
- [ ] Foi picado pelo barbeiro (chupão, chupança, fincão).
- [ ] Recebeu transfusão de sangue antes de 1992.
- [ ] Alguém na sua família tem Chagas.
- [ ] Mora em região endêmica.

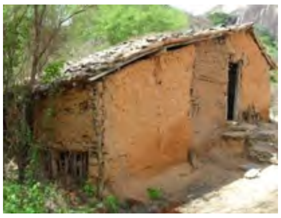
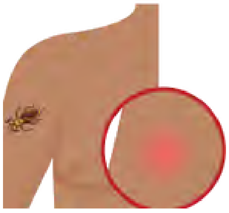
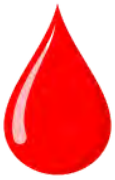
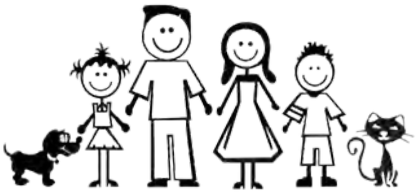
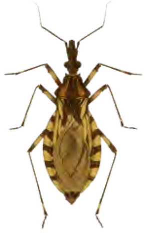

Se você marcou alguma das opções, procure uma Unidade de Saúde!!!

SaMi-Trop

IMPLEMENTA SaMi-Trop

SaMi-Trop · Unimontes – Universidade Estadual de Montes Claros · IMPLEMENTA SaMi-Trop

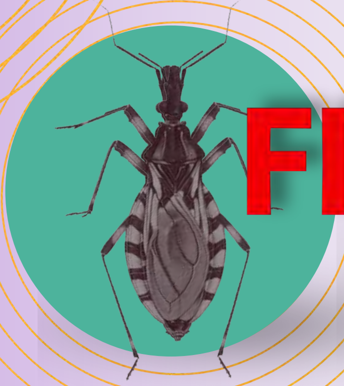

FIQUE ATENTO aos SINTOMAS

FASE AGUDA

- Dor de Cabeça
- Inchaço no rosto
- Febre
- Fraqueza
- Dor no corpo
- Vermelhidão na Pele

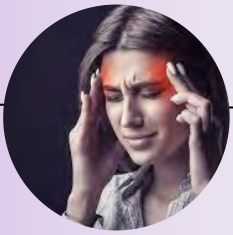
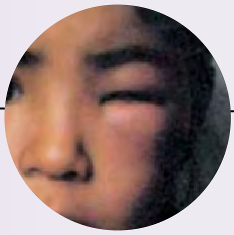
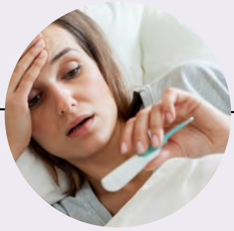
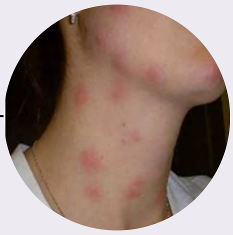

FASE CRÔNICA

- Problemas cardiacos
- Batimentos cardíacos irregulares
- Lesão parecida com um furúnculo
- Inchaço na Barriga
- Problemas digestivos

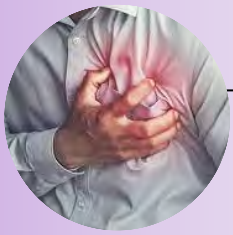
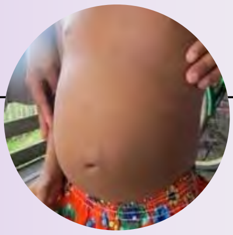
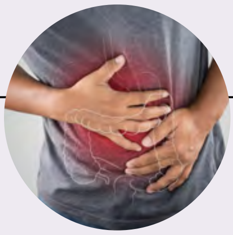
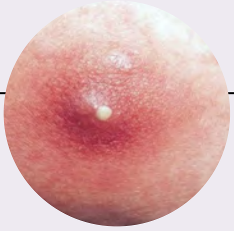

CASO APRESENTE ALGUM SINTOMA, PROCURE UM POSTO DE SAÚDE OU UM HOSPITAL PROXÍMO

CHAGAS TEM TRATAMENTO

Unimontes
Universidade Estadual de Montes Claros

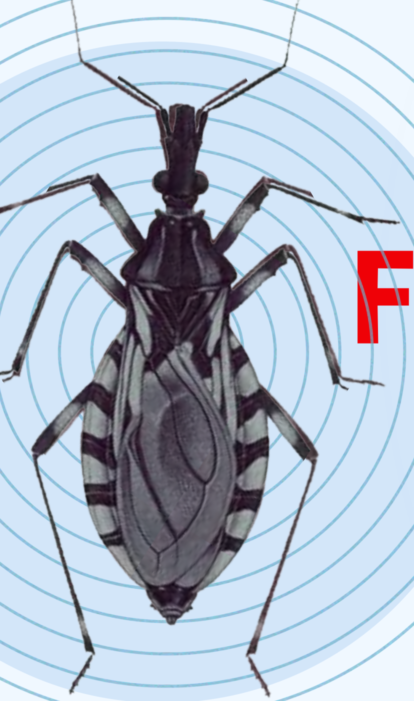

FIQUE ATENTO aos SINTOMAS

CASO APRESENTE ALGUM SINTOMA, PROCURE UM POSTO DE SAÚDE OU UM HOSPITAL PROXÍMO !!!

Fase aguda

- Febre persistente
- Dor de cabeça
- Inchaço no rosto
- Fraqueza
- Dor no corpo
- Vermelhidão na pele

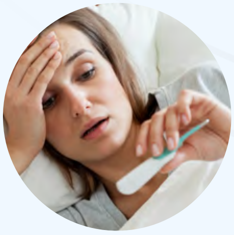
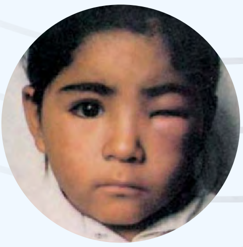
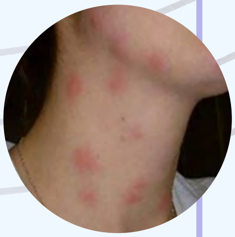
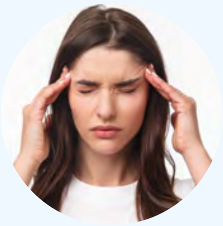
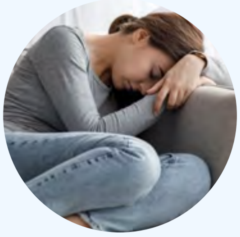

Fase crônica

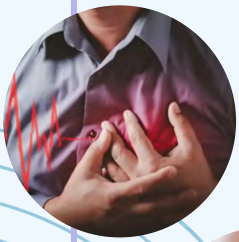
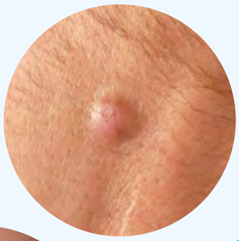
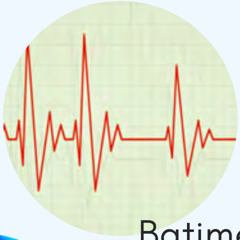
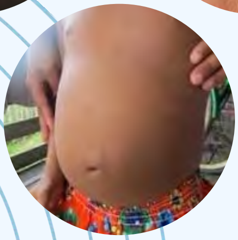
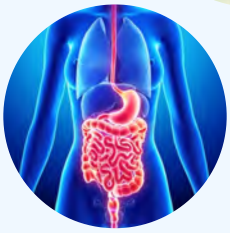

- Problemas digestivos
- Inchaço na Barriga
- Batimentos cardíacos irregulares
- Lesão parecida com um furúnculo
- Problemas cardiacos

CHAGAS TEM TRATAMENTO

SaMi-Trop
IMPLEMENTA SaMi-Trop

FIQUE ATENTO

aos SINTOMAS

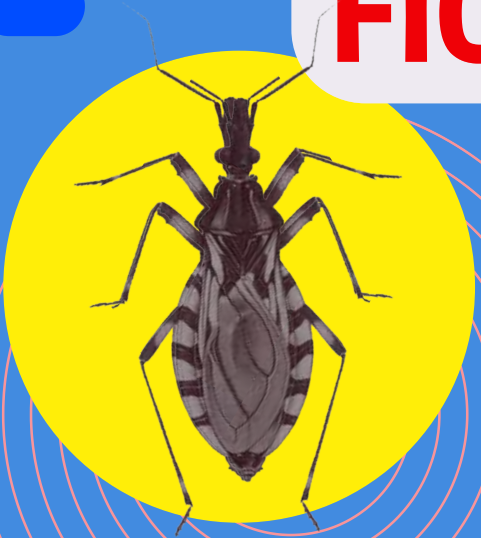

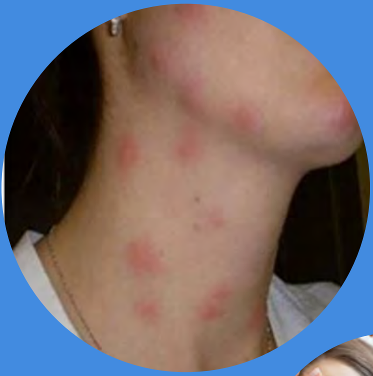

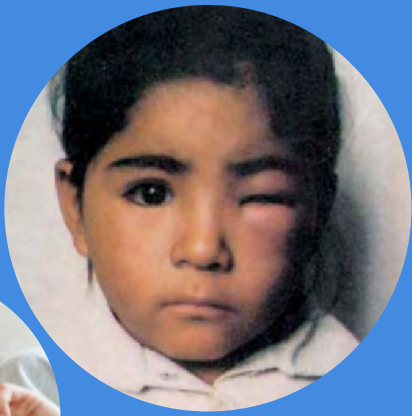

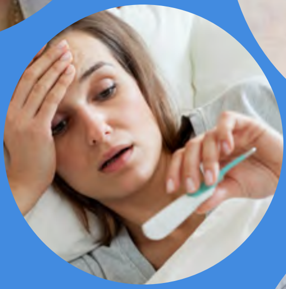

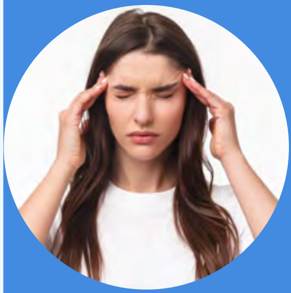

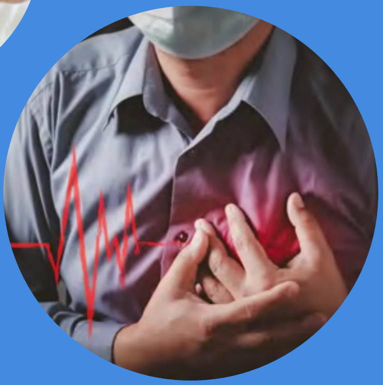

FASE AGUDA

- FEBRE PERSISTENTE (MAIS DE 7 DIAS)
- DOR DE CABEÇA
- FRAQUEZA INTENSA
- INCHAÇO NO ROSTO E PERNAS
- MANCHAS VERMELHAS NA PELE

FASE CRÔNICA

- DISTÚRBIOS CARDÍACOS;
- DOR ABDOMINAL;
- CONSTIPAÇÃO;
- DIFICULDADE PARA ENGOLIR;
- PERTURBAÇÕES DIGESTIVAS.

ÓRGÃOS MAIS AFETADOS

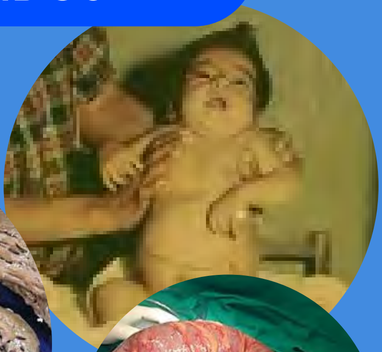

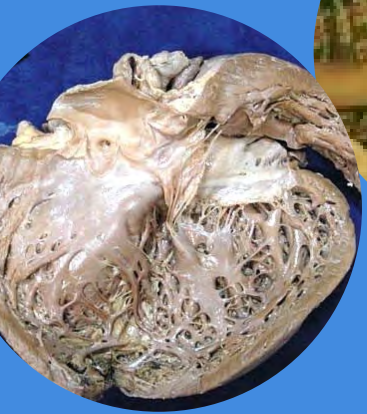

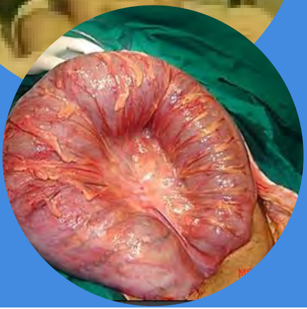

SUS

Unimontes
Universidade Estadual de Montes Claros

PREFEITURA DE FRANCISCO DUMONT
Trabalho é nossa marca

LAFEPE BENZNIDAZOL - 12,5 mg

benznidazol **COMPRIMIDOS**

FÓRMULA: Cada comprimido contém:

benznidazol... 100 mg

excipiente adequado q.s.p. .... 1 comprimido

USO PEDIÁTRICO - USO ORAL

VENDA SOB PRESCRIÇÃO MÉDICA

TODO MEDICAMENTO DEVE SER MANTIDO FORA DO ALCANCE DAS CRIANÇAS

INFORMAÇÕES AO PACIENTE, INDICAÇÕES, CONTRA-INDICAÇÕES E PRECAUÇÕES: VIDE BULA

CONSERVAR EM TEMPERATURA AMBIENTE (ENTRE 15º E 30ºC) PROTEGER DA LUZ E DA UMIDADE

ATENDIMENTO AO CONSUMIDOR DDG(Fone/Fax): 0800.081.1121

Contém: 240 comprimidos

MS: 1.0183.0145.000-0

Unimontes
Universidade Estadual de Montes Claros

SaMi-Trop

FATORES DE RISCO PARA A DOENÇA DE CHAGAS

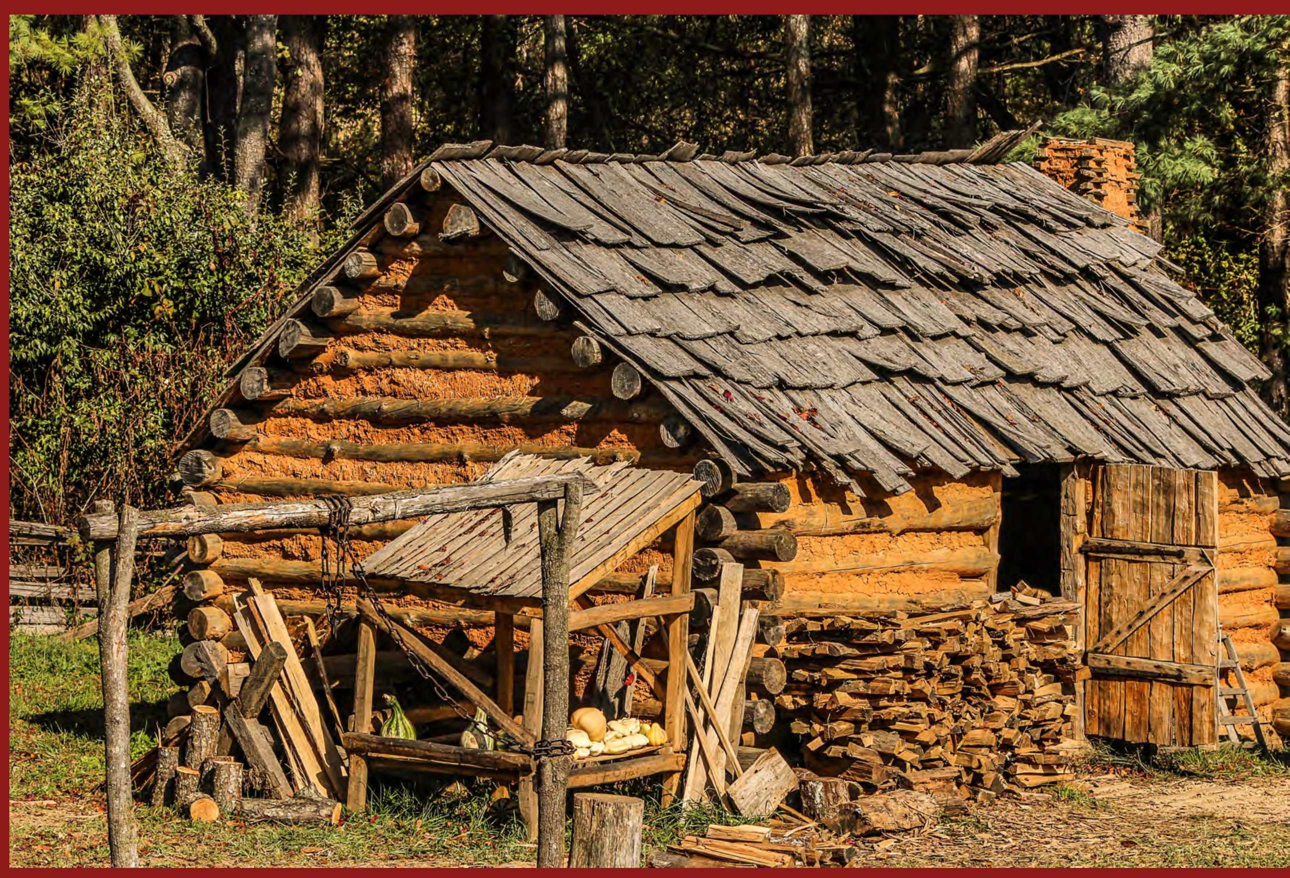

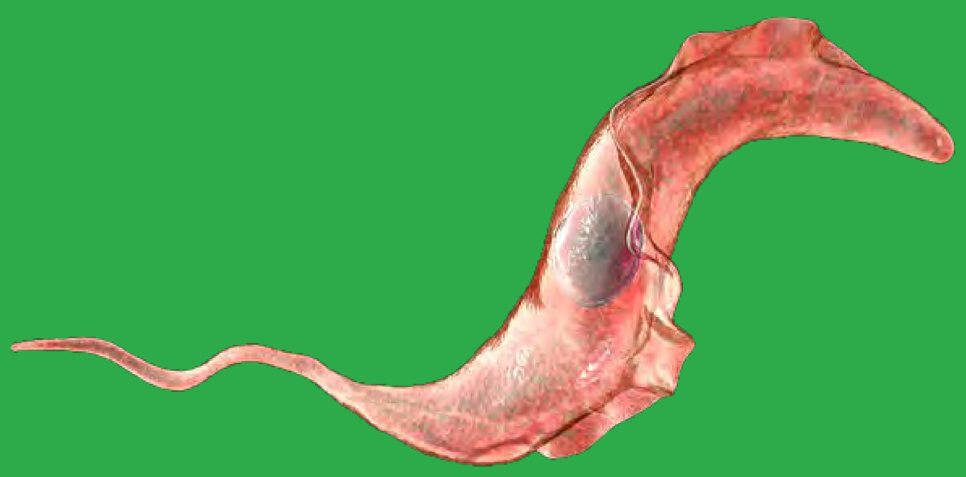

1. Ter residido na infância ou residir em área com relato de presença do barbeiro com registro de infecção pelo **T. cruzi.**

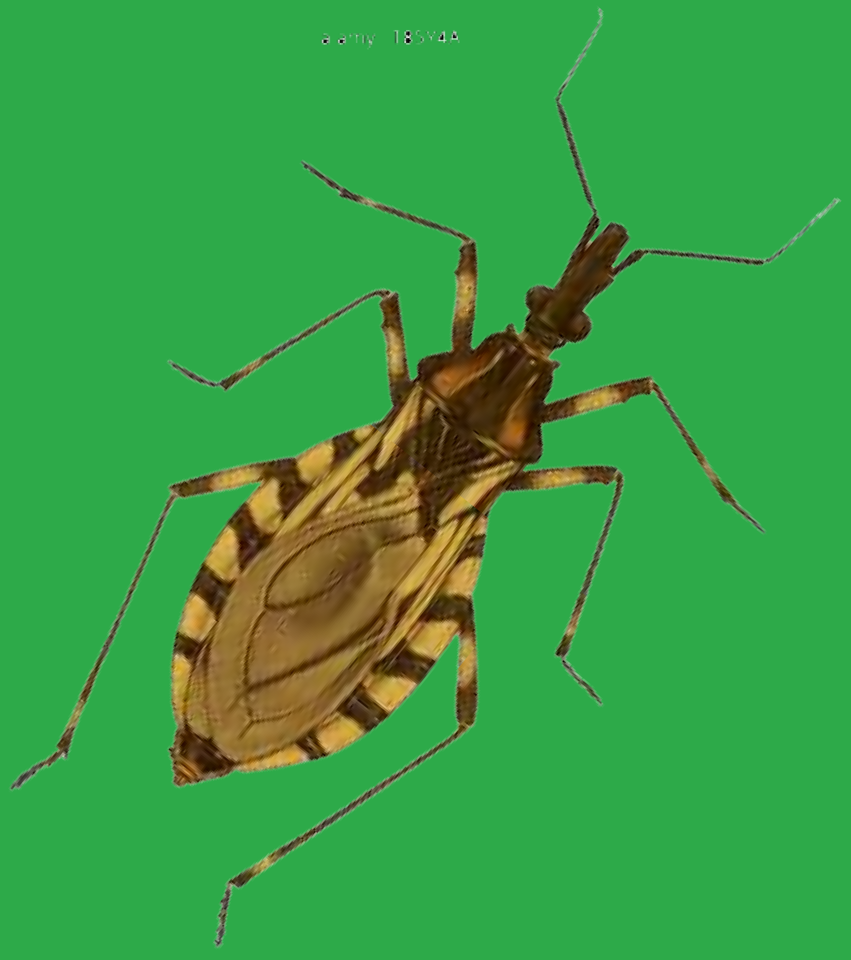

2. Ter residido ou residir em habitação onde possa ter ocorrido o convívio com o vetor (barbeiro).

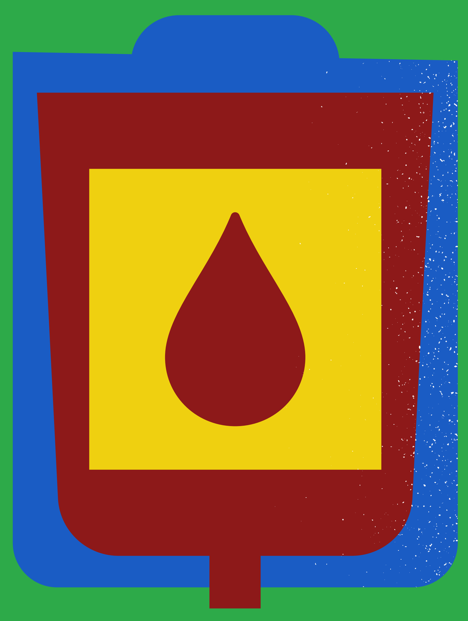

3. Morar ou ter vindo de área com registro de transmissão ativa ou com histórico no passado de ocorrência de transmissão.

4. Ter realizado transfusão de sangue antes de 1992.

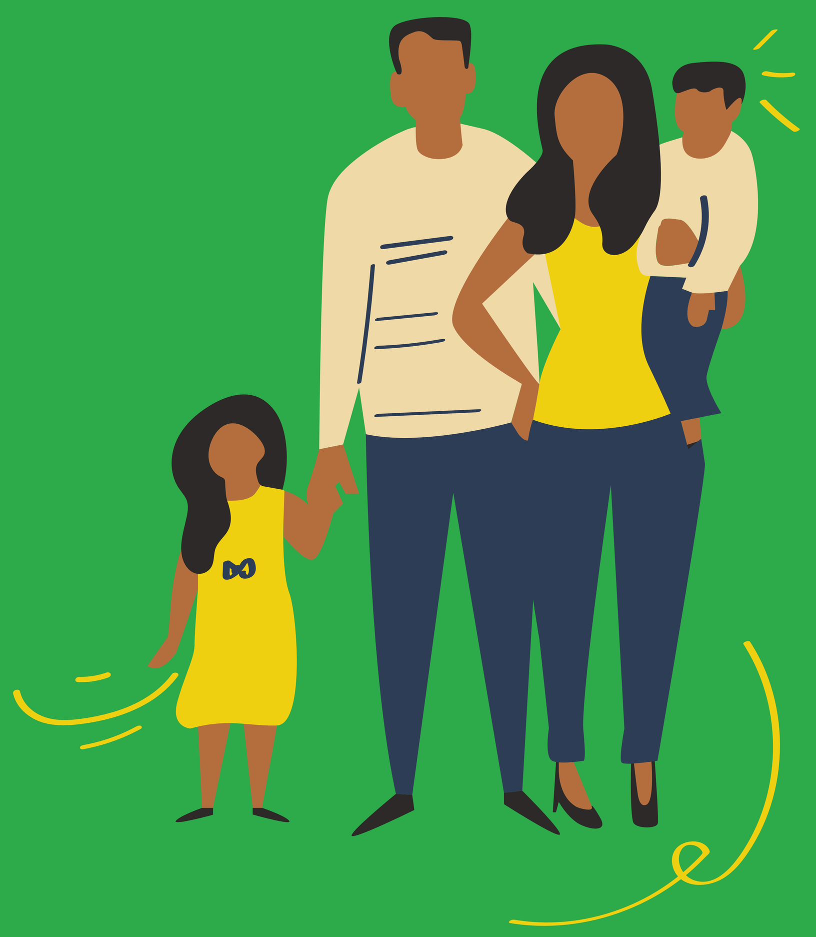

5. Ter convívio frequente com pessoas ou familiares que tenham o diagnóstico para Doença de Chagas, especialmente filhos com mãe comprovada com a infecção.

TRATAMENTO

O tratamento para a doença de chagas é feito com o uso do medicamento Benznidazol.

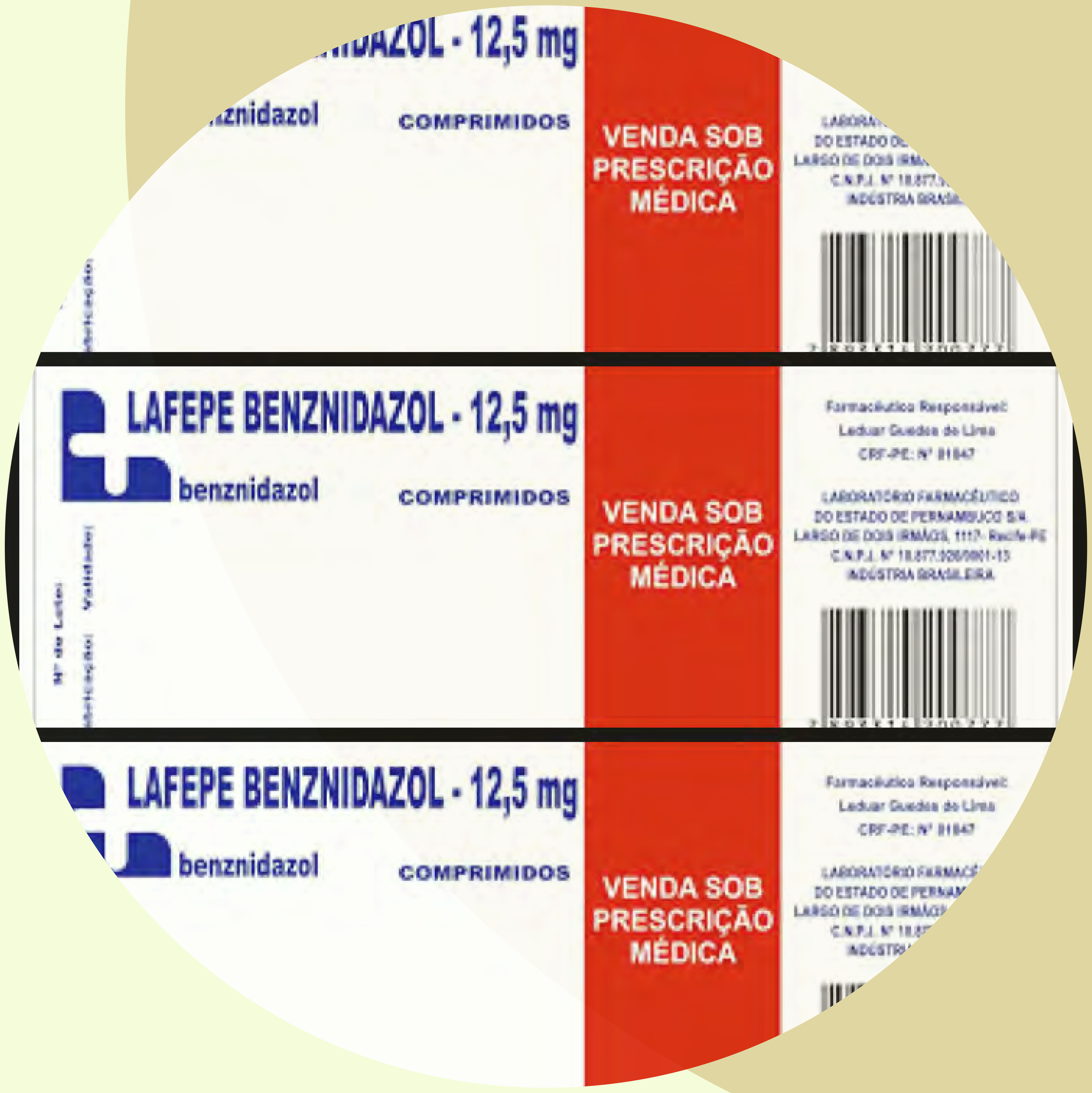

Ele deve ser indicado por um médico, após a confirmação do diagnóstico.

É importante que o tratamento não seja interrompido, os efeitos colaterais da medicação são temporários.

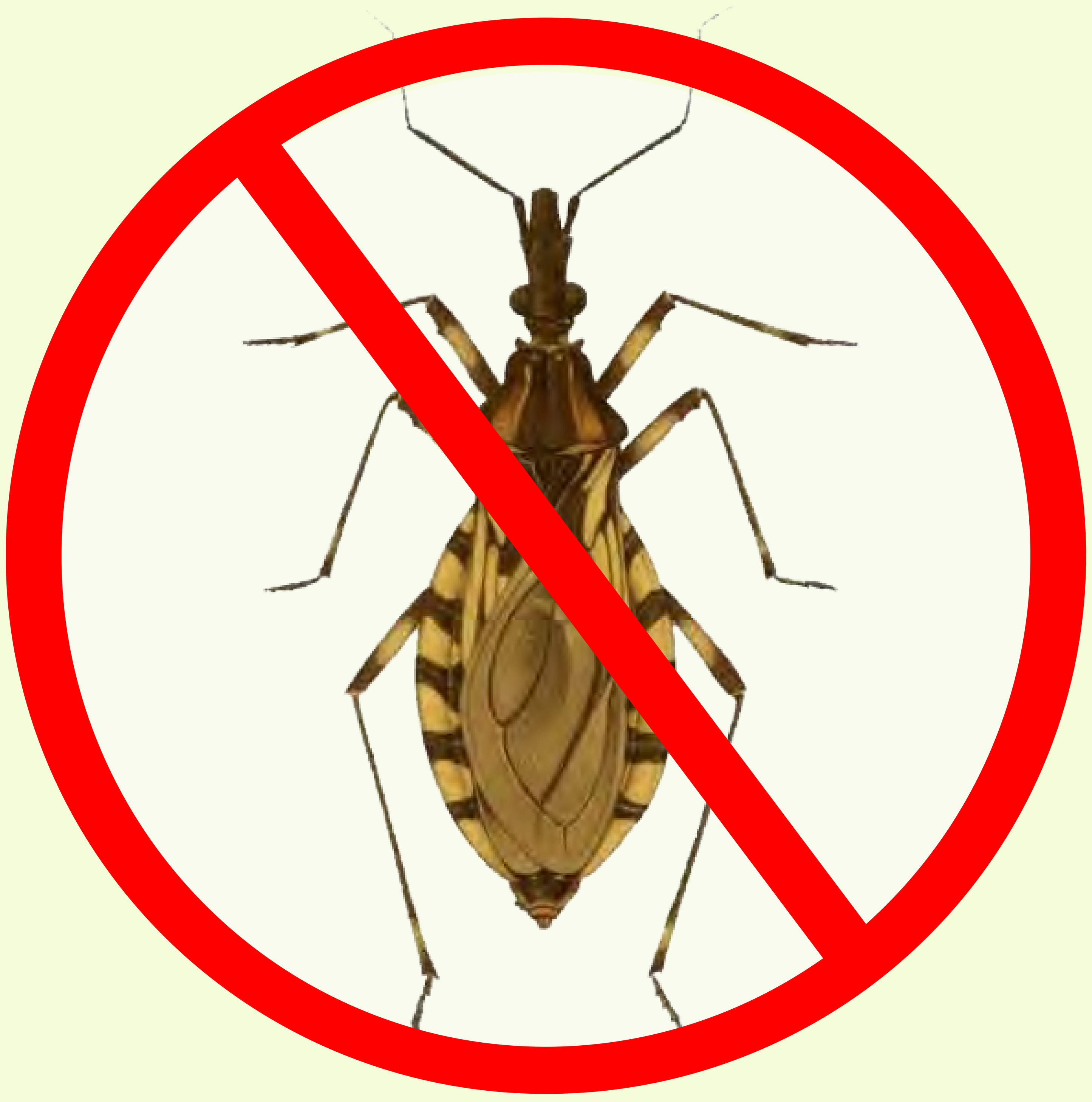

Prevenção é o melhor remédio!

A melhor forma de prevenção é impedir a proliferação do inseto transmissor, o "Barbeiro".

Para isso:

- Evite o acúmulo de madeira, telhas e entulho no quintal.
- Mantenha galinheiros e criatórios afastados de casa.
- Tape frestas e buracos na parede.

Unimontes
Universidade Estadual de Montes Claros

IMPLEMENTA SaMi-Trop

Como ocorre a transmissão da Doença de Chagas?

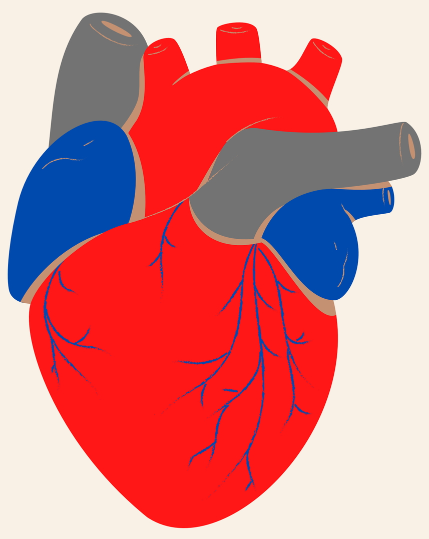

As principais formas de transmissão da doença de Chagas são:

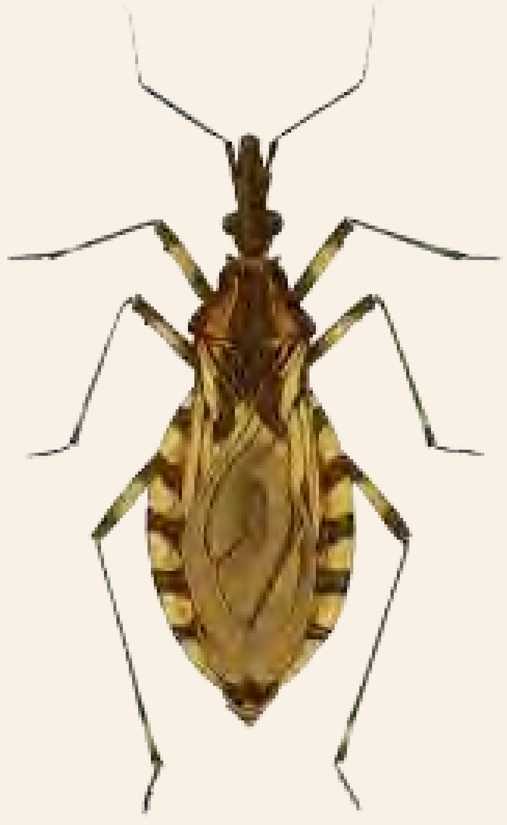

· **Vetorial:** contato com fezes de triatomíneos infectados após o repasto/alimentação sanguínea. A ingestão de sangue no momento do repasto sanguíneo estimula a defecação e, dessa forma, o contato com as fezes.

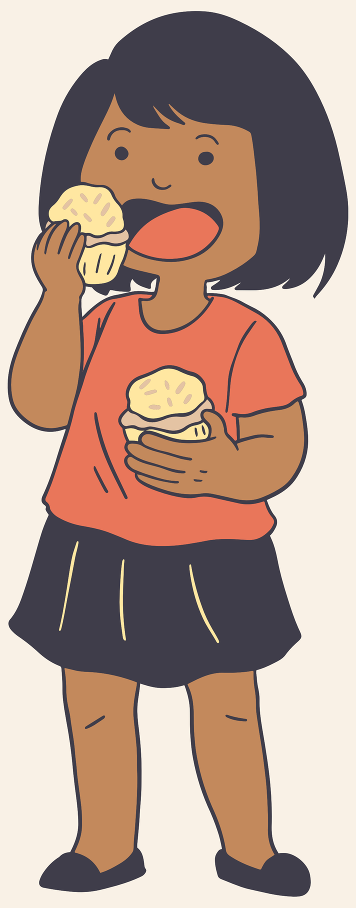

· **Oral:** ingestão de alimentos contaminados com parasitos provenientes de triatomíneos infectados ou suas excretas.

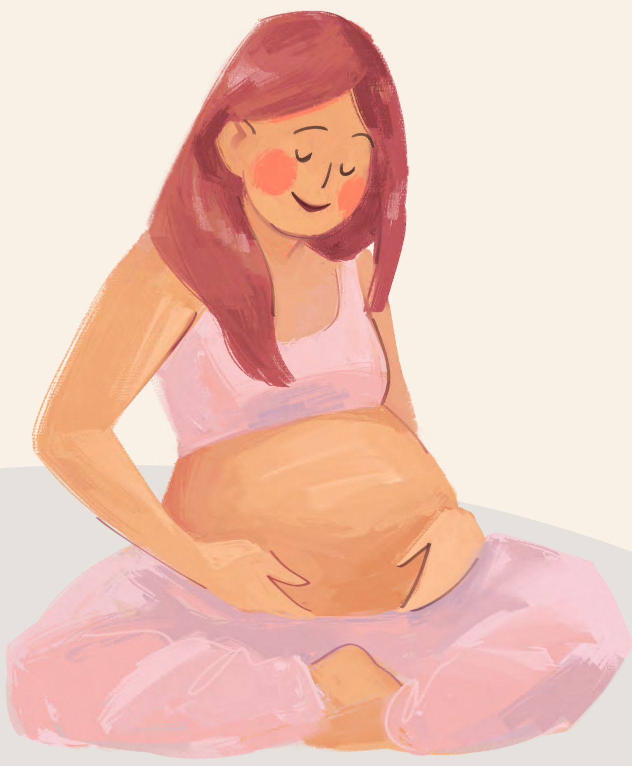

· **Vertical:** ocorre pela passagem de parasitos de mulheres infectadas por T. cruzi para seus bebês durante a gravidez ou o parto.

· **Transfusão de sangue ou transplante de órgãos** de doadores infectados a receptores sadios.

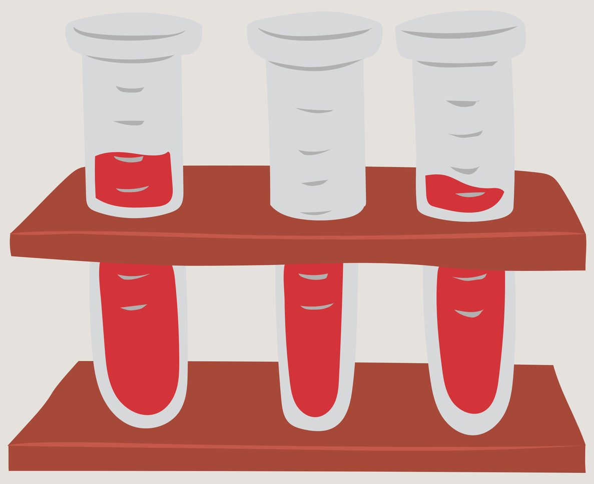

· **Acidental:** pelo contato da pele ferida ou de mucosas com material contaminado durante manipulação em laboratório ou na manipulação de caça.

IMPLEMENTA SaMi-Trop

Unimontes
Universidade Estadual de Montes Claros

VOCÊ JÁ VIU ALGUM DESTES INSETOS?

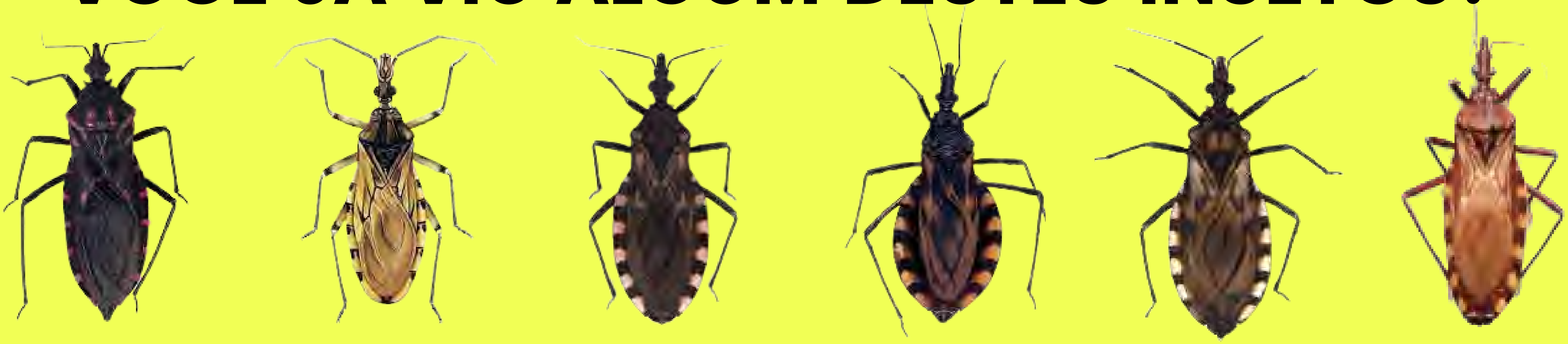

Eles são conhecidos como barbeiro, chupão ou bicudo e podem transmitir a doença de Chagas.

O que fazer quando encontrá-los?

Não os mate!

Capture o barbeiro vivo.

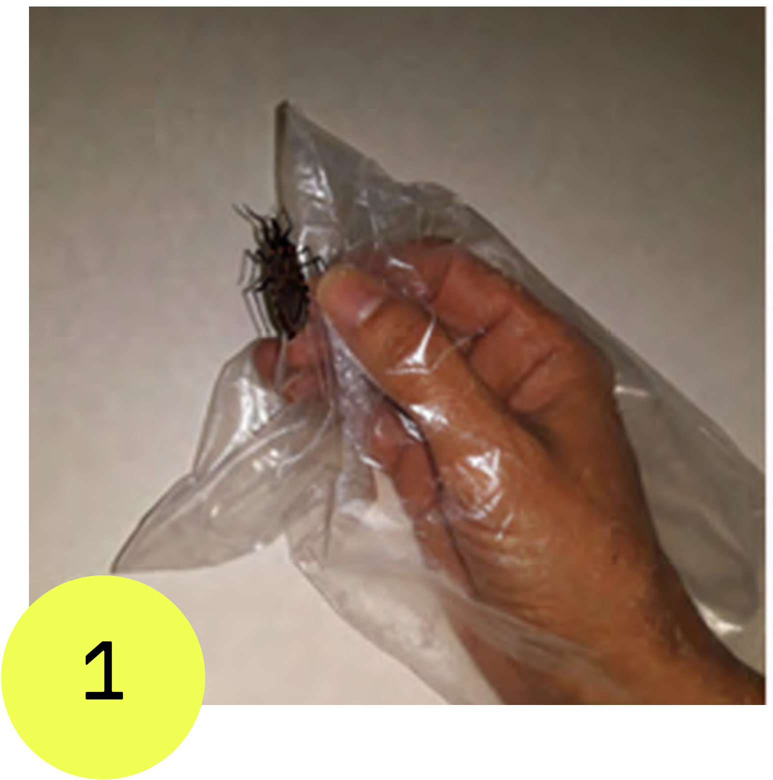

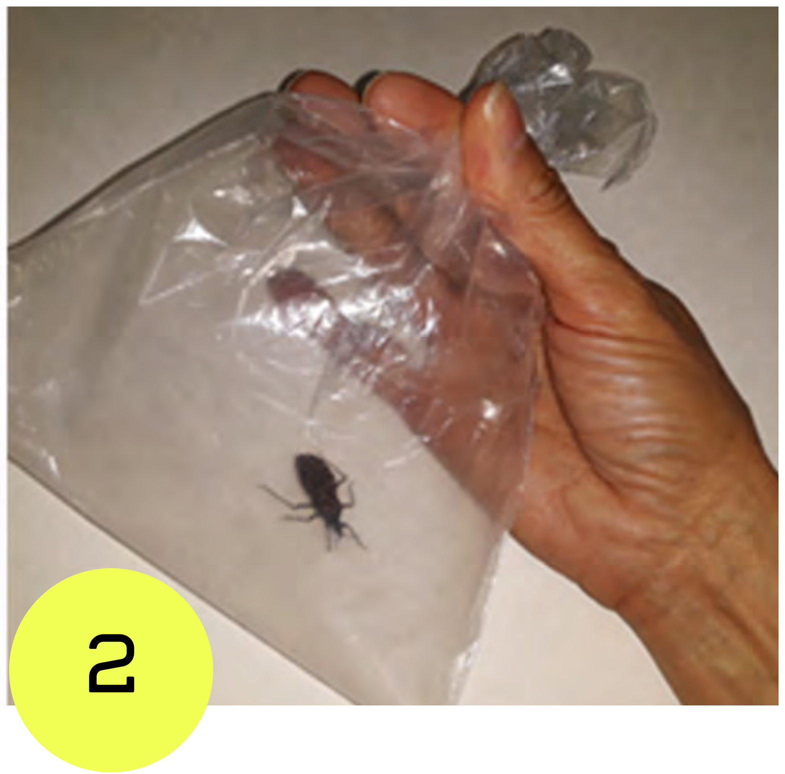

Entregue o barbeiro vivo no
Posto de Informação de Triatomíneos (PIT) ou na
Unidade Básica de Saúde mais próxima da sua casa.

IMPLEMENTA SaMi-Trop
