# Supplementary material for: Implementation Science in the Development of a Care Pathway for Chronic Chagas Disease: An Experience from a Municipality in Minas Gerais
Source: Rev Soc Bras Med Trop. 2026 Feb 9;59:e0381-2025. doi: 10.1590/0037-8682-0381-2025 (PMC12892926; doi:10.1590/0037-8682-0381-2025)
Supplement: Supplementary material 2 [file 1678-9849-rsbmt-59-e0381-2025-md3.pdf]

DOENÇA DE CHAGAS

O QUE É?

É uma doença infecciosa causada pelo *Trypanosoma cruzi.* Ela é transmitida por meio do contato com as fezes do BARBEIRO transmissor da doença ou da ingestão de alimentos contaminados, principalmente açaí e caldo de cana.

VOCE JÁ VIU ALGUM DESTES BARBEIROS?

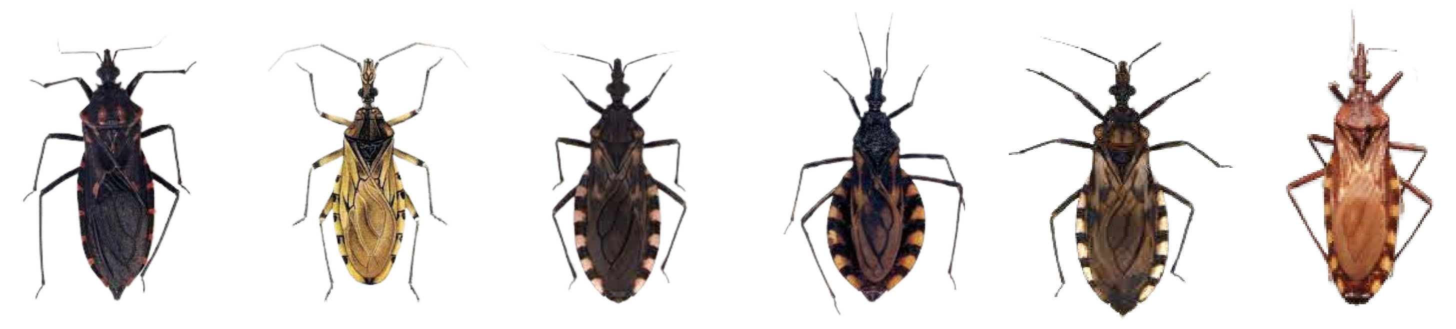

O QUE FAZER SE ENCONTRÁ-LOS?

Capture o barbeiro vivo e entregue no PIT ou no PSF mais próximo de sua casa!

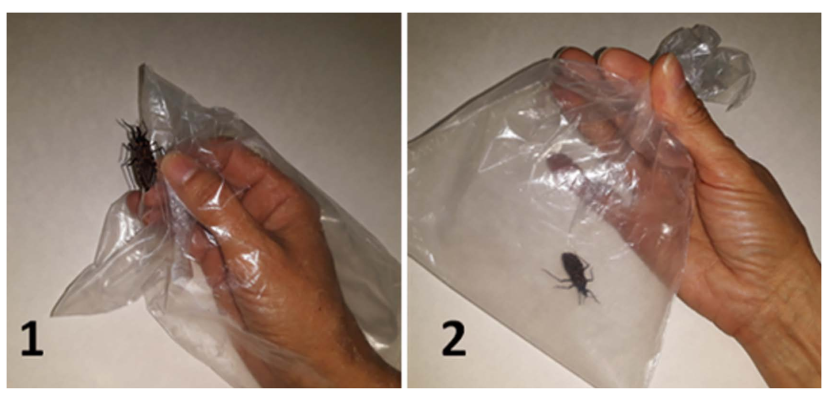

Não mate o barbeiro!

O QUE ELA PODE CAUSAR?

Os pacientes podem ter problemas em órgãos do corpo como:

Coração

Estômago

Intestino

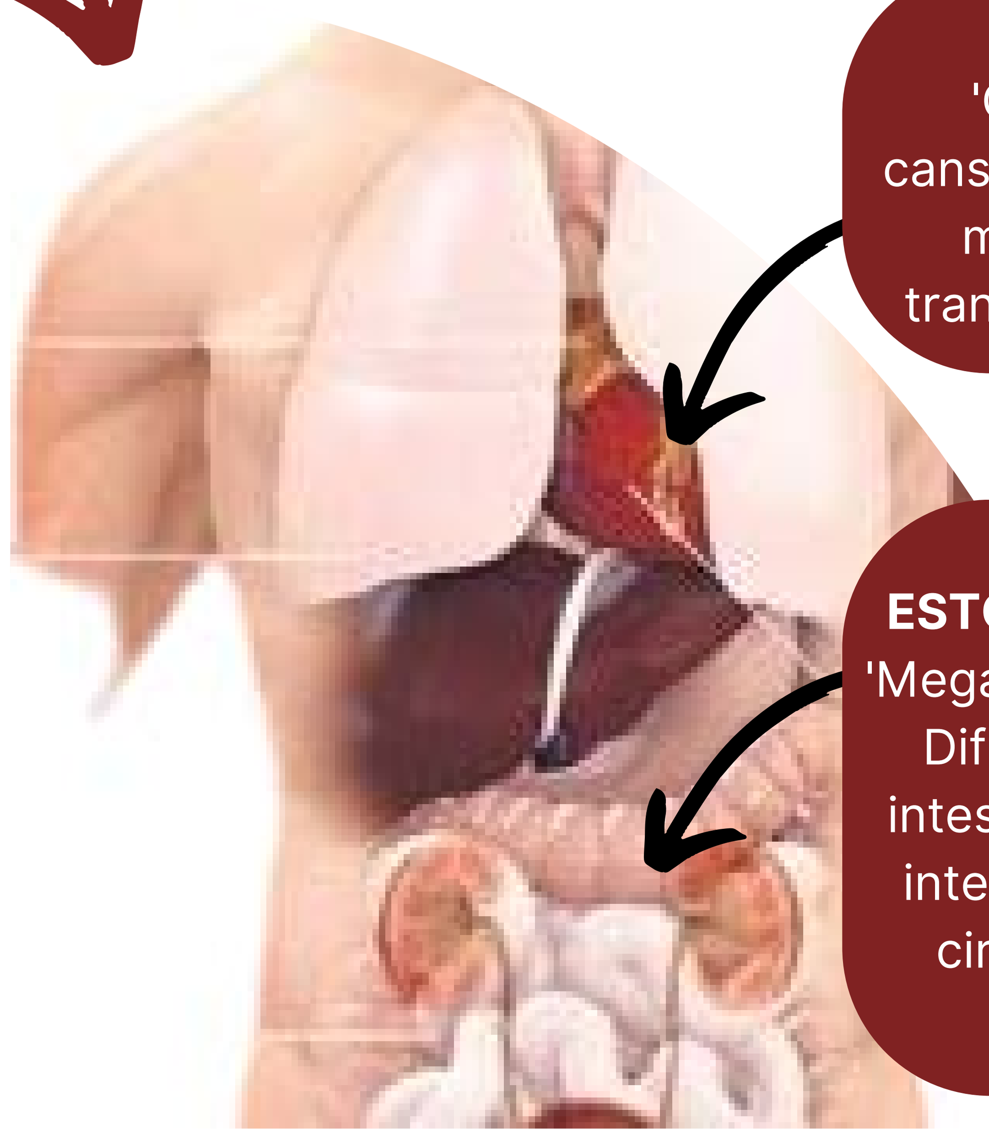

CORAÇÃO
'Coração Inchado'
cansaço, arritmias, uso de marca-passo e até transplante de coração.

ESTÔMAGO E INTESTINO
'Megacolon e Megaesôfago'
Dificuldade pra engolir, intestino preso, obstrução intestinal e até a retirada cirúrgica de partes do intestino.

COMO SABER SE EU TENHO A DOENÇA?

Exame de sangue - Sorologia para Doença de Chagas que é oferecido gratuitamente através do SUS.

QUANDO EU DEVO PROCURAR UMA UNIDADE DE SAÚDE?

- ✓ Mora/morou em locais onde tem barbeiro da Doença de Chagas.
- ✓ Mora ou morou em casas sapê, pau-a-pique, madeira ou barro.
- ✓ Recebeu transfusão de sangue antes de 1992.
- ✓ Foi picado pelo barbeiro da doença de Chagas (Chupão, Fincão).
- ✓ Têm familiares ou mora/já morou com alguém que tenha diagnóstico de doença de Chagas.
- ✓ Mora/morou em região hiper endêmicas (Norte de Minas Gerais e Vale do Jequitinhonha, norte e nordeste do Brasil e Bolívia).
- ⚠ Exames de eletrocardiograma com alterações também podem indicar a realização do teste para a Doença de Chagas.

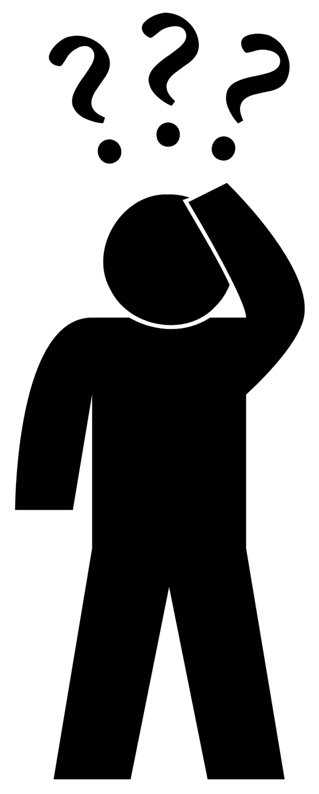

RESULTADO POSITIVO. E AGORA?

Fique calmo!

A Doença de Chagas tem tratamento!

O medicamentos é gratuito no SUS e é usado por 2 meses.

Não é necessário uso contínuo de nenhum medicamento.

Apenas consultas médicas periódicas para acompanhamento.

Quanto mais rápido o tratamento for iniciado, maior a chance de sucesso!

UFSJ CCO
Campus Centro-Oeste Dona Lindu

IMPLEMENTA SaMi-Trop

VOCÊ SABE O QUE É A DOENÇA DE CHAGAS?

É uma doença infecciosa causada pelo *Trypanosoma cruzi*. Ela é transmitida através do contato com as fezes do barbeiro transmissor da doença ou da ingestão de alimentos contaminados (principalmente açai e caldo de cana).

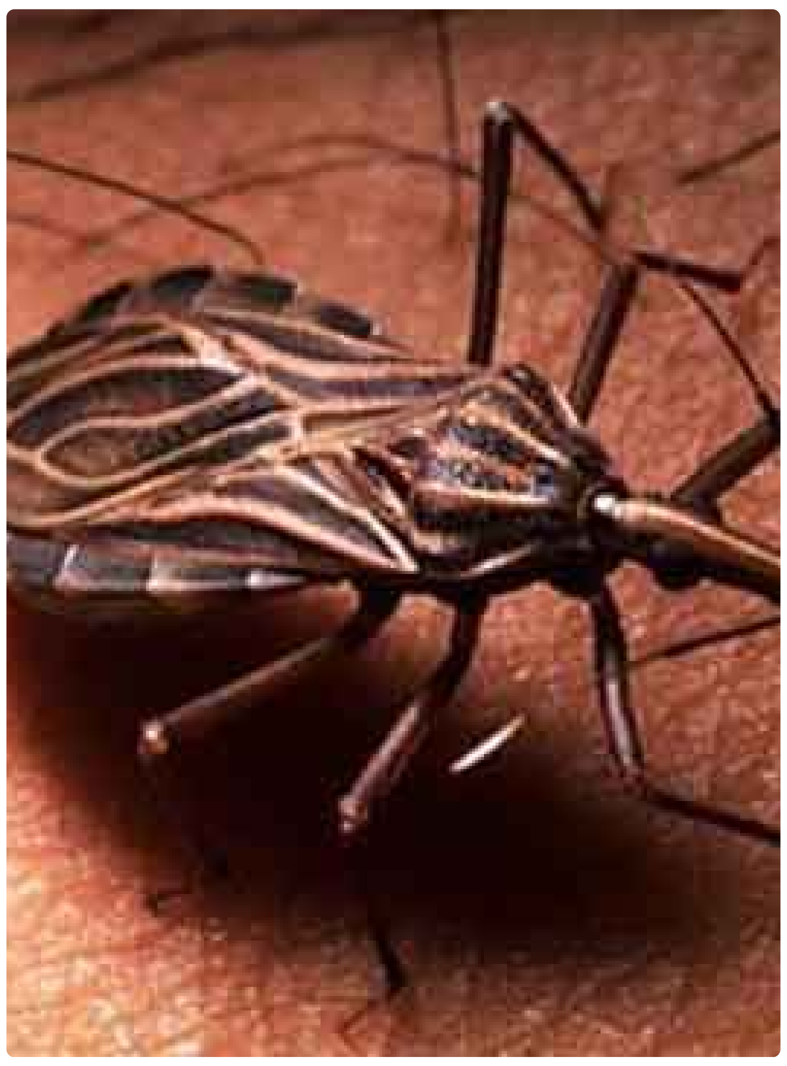

O QUE ELA PODE CAUSAR?

Se não identificada e tratada no momento certo, os pacientes podem ter problemas no estômago, intestino e coração. Casos mais graves podem necessitar de marca-passo e até mesmo transplantes de coração.

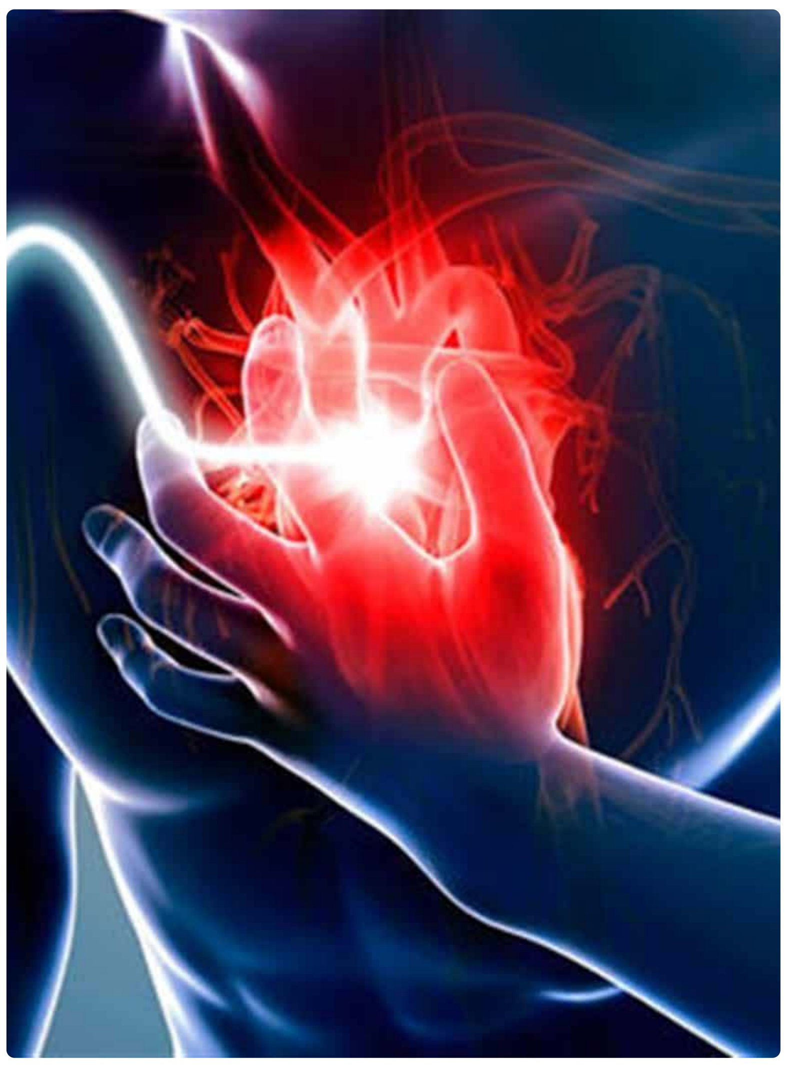

COMO SABER SE EU TENHO A DOENÇA?

O diagnóstico é feito através de um simples exame de sangue. O tratamento é realizado com medicamentos, por aproximadamente 2 meses. Quanto mais rápido o tratamento for iniciado, maior a chance de sucesso!

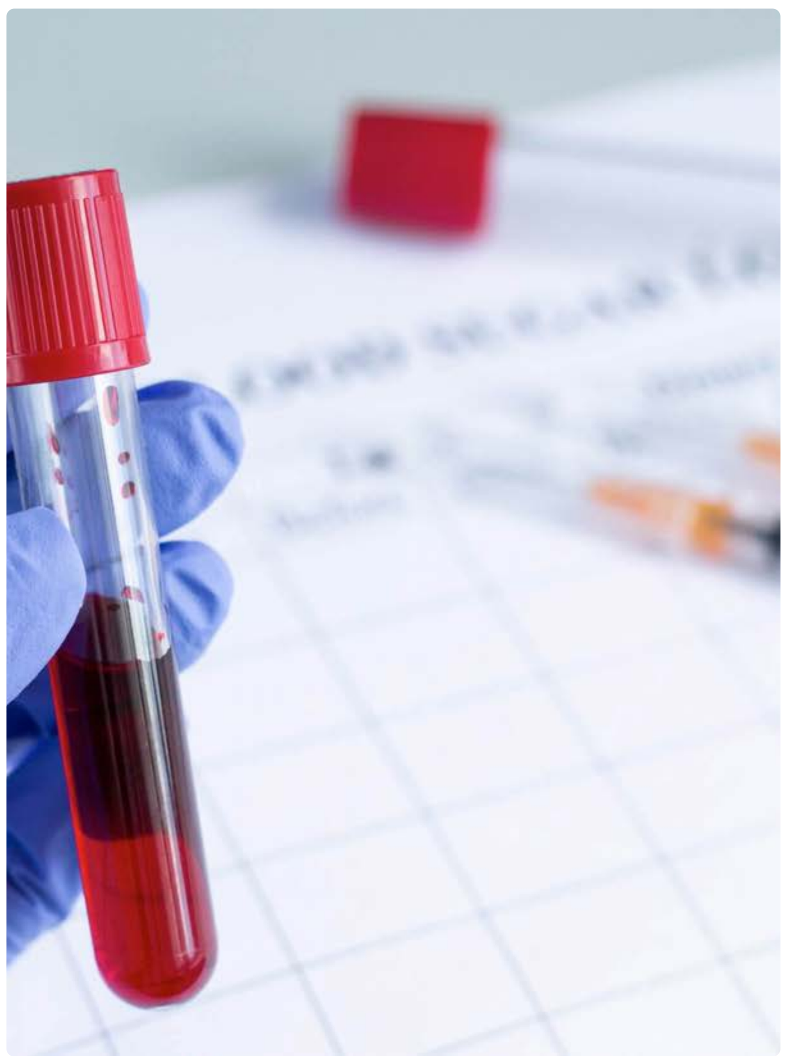

Se você responder sim para uma das perguntas abaixo, procure sua unidade de saúde.
Todos os procedimentos são gratuitos através SUS.

- ✓ Mora/morou em locais onde tem barbeiro da Doença de Chagas.
- ✓ Mora ou morou em casas sapê, pau-a-pique, madeira ou barro.
- ✓ Recebeu transfusão de sangue antes de 1992.
- ✓ Foi picado pelo barbeiro da doença de Chagas (Chupão, Fincão).
- ✓ Têm familiares ou mora/já morou com alguém que tenha diagnóstico de doença de Chagas.
- ✓ Mora/morou em região hiper endêmicas (Norte de Minas Gerais e Vale do Jequitinhonha, norte e nordeste do Brasil e Bolívia).

⚠ Exames de eletrocardiograma com alterações também podem indicar a realização do teste para a Doença de Chagas. Converse na sua Unidade de Saúde.

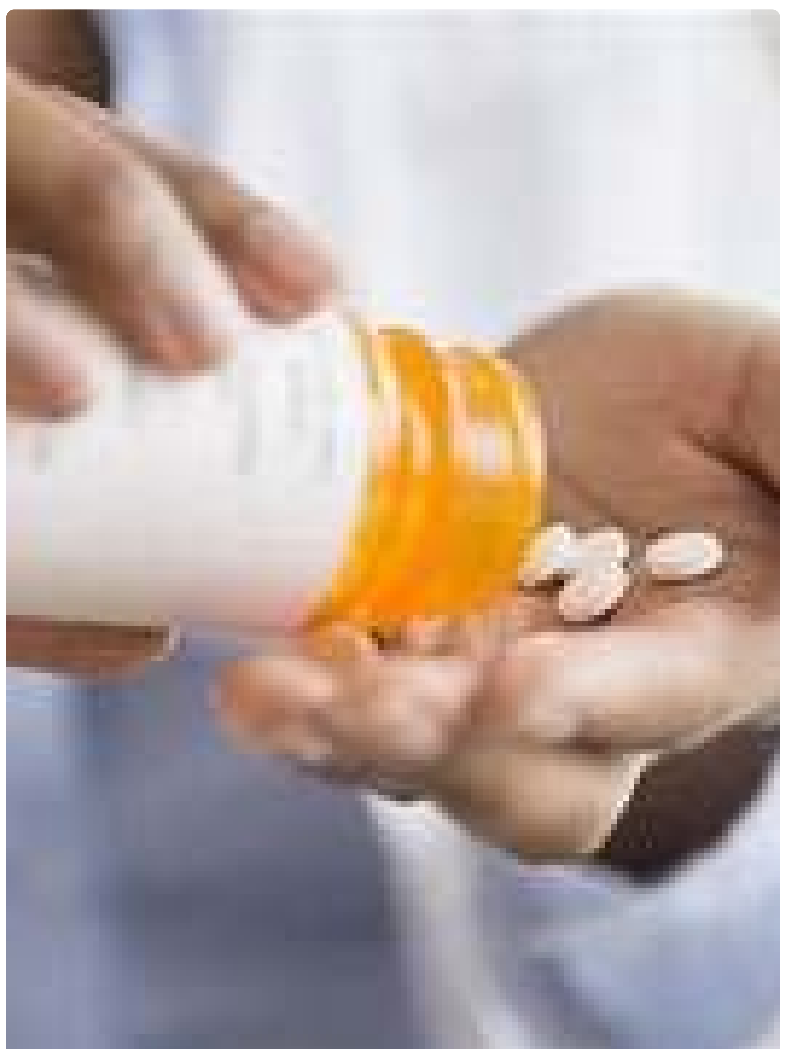

IMPLEMENTA SaMi Trop

UFSJ
